# A Modular Approach to Atropisomeric Bisphosphines of Diversified Electronic Density on Phosphorus Atoms

**DOI:** 10.3390/molecules27175504

**Published:** 2022-08-27

**Authors:** Oleg M. Demchuk, Aleksandra Martyna, Mateusz Kwaśnik, Katarzyna Szwaczko, Dorota Strzelecka, Barbara Mirosław, Kazimierz Michał Pietrusiewicz, Zofia Urbanczyk-Lipkowska

**Affiliations:** 1 Institute of Biological Sciences, Faculty of Science and Health, The John Paul II Catholic University of Lublin, Konstantynów 1J/4.03, 20-708 Lublin, Poland; 2 Faculty of Chemistry, Maria Curie-Sklodowska University in Lublin, Gliniana 33, 20-031 Lublin, Poland; 3 Institute of Organic Chemistry of the Polish Academy of Sciences, Kasprzaka 44/52, 01-224 Warsaw, Poland

**Keywords:** axially chiral biaryls, atropisomers, chiral bisphosphines, *C_2_*-symmetry, CP-bond formation, enantiomer separation, BIMOP, MeO-BIPHEP, BIPHEMP, TetraPheMP, BIMAP, BIClP

## Abstract

The series of *C_2_*-symmetric biaryl core-based non-racemic bisphosphines possessing substituents of different electronic properties: both EDG and EWG were obtained in a short sequence of good yielding transformations, started from commercial 1,3-dimethyl-2-nitrobenzene. Several different approaches leading to the desirable ligands were practically evaluated. Notably, the synthesis of the entire series of ligands could be performed with the utilization of a single early-stage precursor DIDAB (6,6′-diiodo-2,2′,4,4′-tetramethylbiphenyl-3,3′-diamine), which could be easily obtained in enantiomerically pure form. The obtained compounds at concentrations of 50 and 200 µM showed various biological activity against normal human dermal fibroblast, ranging from inactivity through time-dependent action and ending up with high toxicity.

## 1. Introduction

**Figure 1:**
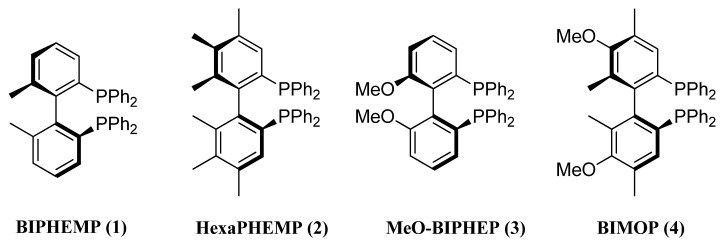
Atropisomeric biaryl bisphosphines.

*C_2_*-symmetrical biaryls are key structural motifs in a number of biologically active natural products and drugs [1,2,3,4,5,6,7,8,9]. The axially chiral biaryl framework is also an essential element in a variety of privileged ligands in asymmetric catalysis, which, as chiral bisphosphines as well as monophosphines, are widely used in asymmetric transformations such as hydrogenation, hydrosilylation, hydrocyanation, isomerization, etc. [10,11,12,13,14]. Over the last decades, the development of novel axially chiral ligands attracted significant attention. Following success of a well-known **BINAP** [15,16,17], other *C_2_*-symmetric chiral biaryl bisphosphine ligands were developed. Among the others, the transition metal complexes of such ligands such as **BIPHEMP (1)** [18,19,20], **HexaPHEMP** (**2**) [21], **MeO-BIPHEP (3)** [22,23,24], **BIMOP (4)** [25,26], and some of their systematic structural variations (Figure 1), were used as chiral catalysts of special properties.

Despite the fact that atropisomeric bisphosphines are important ligands used in many asymmetric reactions [13,27,28,29], practical methods of their preparation still remain challenging. The classical synthesis of the novel axially chiral bisphosphine ligands usually involves aryl–aryl coupling (Ullmann coupling) of different kinds of aryl backbones such as naphthyl, phenyl or heteroaryl, and then resolution via crystallization followed by deoxygenation of the P = O group [30,31,32,33,34]. In more rare cases, an introduction of -P(III)R_2_ to the chiral non-racemic binaphthyl backbone could also been explored [35,36,37,38]. Other strategies for the synthesis of chiral biaryl backbone include direct atroposelective biaryl coupling [39,40], as well as atroposelective aryl ring formation by cycloaddition reaction, and resolution and desymetrization methods etc. [1,41,42,43,44,45,46]. Although new developments in the synthesis of chiral biaryl skeleton are significant, some of them suffer from certain restrictions such as narrow substrate scope, low efficiency and inefficient stereocontrol.

Herein, we describe an efficient strategy allowing access to axially chiral biaryls bearing phosphorus functionalities and diversified substituents at 5,5′-positions. Such phosphines are known to be potent bidentate ligands for transition metals to be used in different types of catalytic asymmetric transformations. The particular design of the ligands implies that the complexes derived from them will adopt the same stereometry, but the electronic properties of the transition metal will depend on the substituent introduced in ligand core [28]. The ligands were obtained in a short sequence of good-yielding transformations, which started from commercial 1,3-dimethyl-2-nitrobenzene and leading through the formation of a single universal 5,5′-diamine precursor **DIDAB** (6,6′-diiodo-2,2′,4,4′-tetramethylbiphenyl-3,3′-diamine, **5**). We also report the preparation of a new *C_2_*-symmetrical **BIMOP** (**4a**) related ligand [26] with dimethylamine group at 5,5′-positions, hereafter named **BIMAP** (**4b**), and its successful optical resolution carried out with the use of the chiral cyclopalladated derivative and/or by the resolution of the isomers using HPLC with a chiral stationary phase column. The classical approach to the new chiral non-racemic electron-deficient ligand **BIClP** (**4c**) with chlorine atoms at 5,5′-positions is reported as well. Our approach was also optimised to obtain the highly known and efficient ligands **TetraPHEMP** (**4d**) [18] and BIMOP [26].

## 2. Results and Discussions

### 2.1. Synthesis of Racemic Precursors

**Scheme 1:**
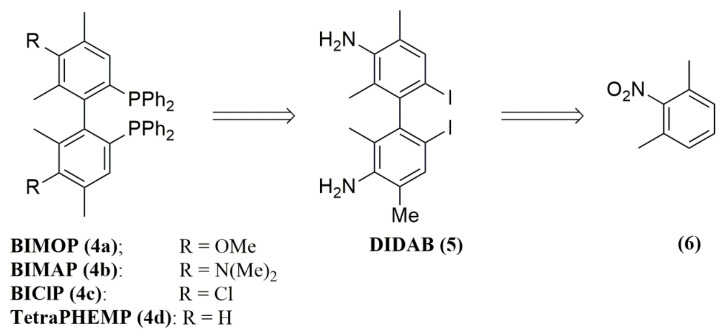
Retrosynthetic analysis of the biphenyl bisphosphines.

An efficient route to a series of desired atropisomeric *C_2_*-symmetrical phosphines is based on the utilisation of a single chiral precursor possessing such function group which could be easily converted to several others, and that at the same time allows the enantioseparation of racemic compound. Thus, the diamine-substituted diiododiaminobiaryl **DIDAB** (**5**) (Scheme 1) was selected for this purpose.

The most direct route to **DIDAB** would involve iodation of a commercially available 2-nitroxylene, followed by the Ullmann coupling reaction, and then a reduction in the nitro group. The series of ligands represented by **BIMOP**, **BIMAP**, **BIClP**, and **TetraPheMP** could be obtained subsequently from the racemic or resolved as **DIDAB** in few steps.

**Scheme 2:**
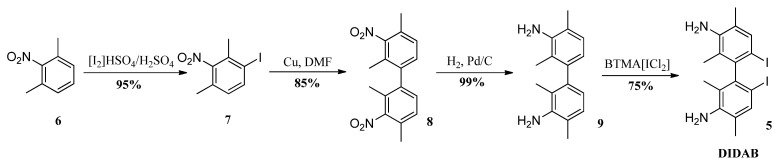
Synthesis of DIDAB.

Thus, commercially available 1,3-dimethyl-2-nitrobenzene was converted into iodoarene (**7**) in concentrated sulfuric acid using I_3_HSO_4_ as the iodine source in a good yield of 95% (Scheme 2). Then, iodoarene was homocoupled by means of Ullmann coupling reaction. It is worth noting that in order to obtain higher yield, the prior activation of the copper surface was necessary. Hence, treating the commercially available reagent with an acidic solution of copper(II) nitrate increased the yield from 50% to 85%. The initial attempts to reduce dinitrobiphenyl **8** to the diamine derivative **9** using a mixture of iron in hydrochloric acid yielded unsatisfactory results. Utilization of LiAlH_4_ for this purpose seemed to be an inconvenient and expensive approach on a larger scale. Finally, the desired diamine derivative **9** was obtained in 99% yield using palladium catalyst (10% Pd/C) under hydrogen pressure of 150 Atm at 150 °C. Next the compound **9** was converted into racemic **DIDAB** (**5**) via iodination with the benzyltrimethylammonium dichloroiodate complex (BTMA-ICl_2_) according to the procedure described in the literature [47]. Usage of alternative iodizing reagents such as ICl or I_2_ for the introduction of iodine atoms at the 2,2′-positions, resulted in much lower selectivity and/or lower conversion of the substrate.

**Scheme 3:**
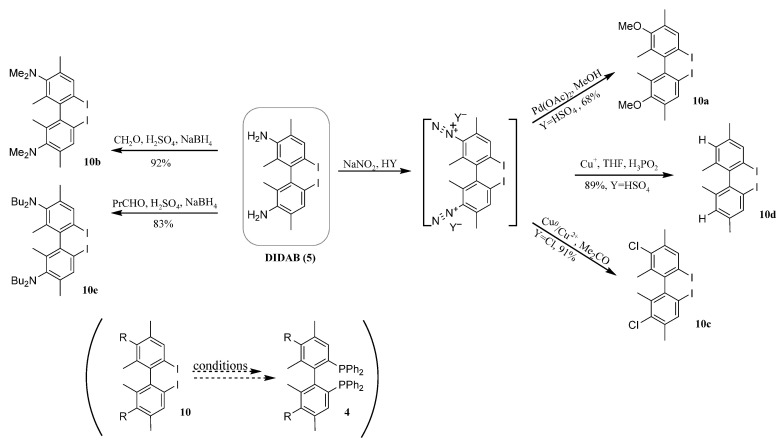
Synthesis of precursors **10a–e**.

**DIDAB**, as the designed universal precursor of all planned chiral bisphosphines, was subsequently transformed by classical transformation of amino groups as presented on Scheme 3, into a series of diiodobiphenyl derivatives with different substituents at 5,5′-positions to be used in further phosphorylation reaction steps.

To obtain diamine derivatives **10b** and **10e**, the amino groups of **DIDAB** were alkylated with formaldehyde and butyraldehyde in acidic aqueous medium under sodium borohydride reductive conditions [48,49]. The desired products were obtained in good yields 92% and 83%, respectively. **DIDAB** was also subjected to the bisdiazotation reaction followed by the substitution of diazonium groups with chlorine (Sandmayer reaction). That yielded compound **10c** in 91%. The dimethoxy derivative **10a** was obtained in the reaction of bisdiazonium salt with methanol catalysed by Pd(OAc)_2_ in 68% yield, while the reductive elimination reaction of that salt with aqueous H_3_PO_2_ in the presence of catalytic amounts Cu_2_O leads to product **10d** in 89% yield.

**Scheme 4:**
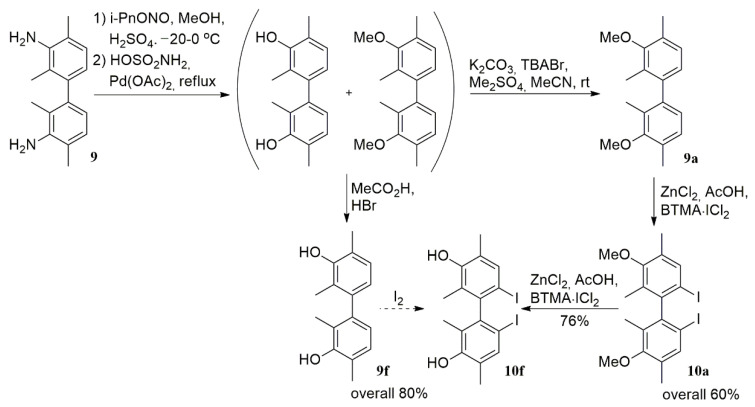
Alternative route to precursor **10a**.

Alternatively, racemic compounds **10a** could be obtained from the early precursor, diamine **9,** in the sequence of high-yielding reactions as presented on Scheme 4 in good 60% overall yield. The iodination reactions of other 3,3′-diamino and 3,3′-dimethoxy substituted 2,2′,4,4′-tetramethylbiphenyls were not as efficient or selective.

### 2.2. Synthetic Route to Bisphosphines

**Scheme 5:**
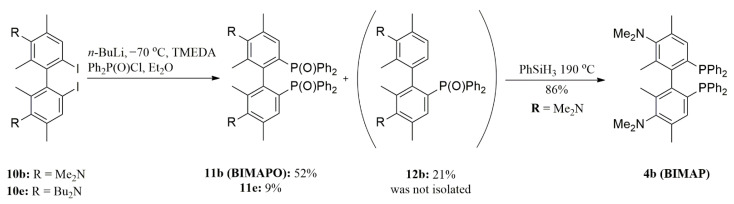
Synthesis of **BIMAP** ligand.

The racemic bisdimethylaminosubstituted ligand **BIMAP** was synthesized in the sequence of reactions leading from **10b** (Scheme 5). A low temperature deiodolithiation reaction, in which combination of *n*-BuLi and TMEDA was found to be the most efficient, leads to the reactive intermediate, suitable for phosphorylation. The amount of the base was found to be crucial to the success of the reaction, and in particular 3.1 equivalents of TMEDA and 2.1 equivalents of *n*-BuLi (1.3M in hexane) were the optimum amounts of lithiation reagents. The lithiated intermediate was then exposed to Ph_2_P(O)Cl to obtain the **BIMAPO** in a good yield of up to 52%. The byproduct of the reaction was the monophosphine oxide (**12b,** 21% yield). Its formation was evidence of the completion of the lithiation process and indicates the difficulty, probably due to the steric hindrance, during the phosphorylation step. The obtained **BIMAPO** was efficiently reduced to **BIMAP** with an excess of phenylsilane at elevated up to 190 °C temperature. Such remarkable high reactivity of triaryl phosphine oxide towards the deoxygenation reaction could be understandable taking into consideration that phosphorus atom received significant injection of electronic density induced from nitrogen [28], what is known to facilitate the deoxygenation reaction [50].

The **BIMAPO** synthesis pathway, shown above, was applied also in the case of transformation of another substrates: **10e**, which leads to the bis(di-*n*-buthylamino)-substituted bisphosphine dioxide **11e**, and chlorosubstituted derivative **10c,** which leads to **BIClPO (11c) in low yields**.

**Scheme 6:**
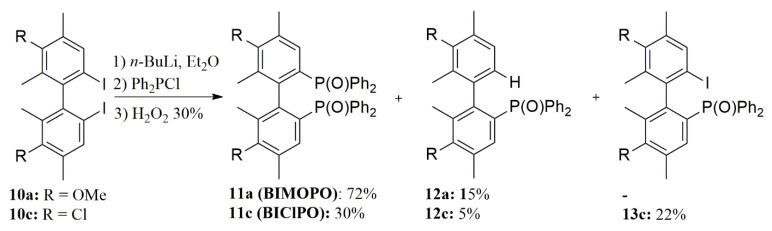
Synthesis of racemic bisphosphine dioxides.

Unfortunately, the product **11e** was unstable in oxidative conditions even as mild as air exposure. That could be rationalized by the tendency of the electron-rich amines to be oxidized in an unselective manner. The compound **10c,** comparison to compounds **10b,** was quite stable in oxidative media, but less reactive in reaction with *n*-BuLi, so the reaction conditions were modified accordingly. The temperature of the lithiation process was elevated during the reaction from −40 up to + 10 °C, and the reaction time was prolonged to 18 h. We found that the reaction of the bislithium derivative with Ph_2_P(O)Cl mostly furnishes product **12c** in low yield. In turn, an arylphosphine moiety was introduced by reaction with more active diphenylchlorophosphine without the addition of TMEDA (Scheme 6) in much better yields. Since the chromatographic isolation of the product formed was impossible in the studied cases, the treatment of the reaction mixtures with hydrogen peroxide in basic environment was applied to obtain corresponding phosphine oxides. It turned out that oxidation step proceeded very slowly, what indicates the high stability of the electronically poor phosphine. Two monophosphine oxides bearing an unreacted iodide group **13c** or a hydrogen atom at the 2-position **12c** were isolated from the reaction mixture in yield of 22% and 5%, respectively. In turn, bisphospnine oxide **BICLPO (11c)** was isolated in 30% yield. These observations indicate that the lithiation process is a limiting factor for the efficiency of the phosphorylation reaction but some better results could be obtained if Ph_2_PCl was used instead of less reactive Ph_2_P(O)Cl derivative.

In the synthesis of bisphosphine oxide **BIMOPO** (**11a**) bearing the MeO–substituents at 5,5′-positions of biaryl skeleton, the precursor **10a** was used. The phosphorylation reaction proceeded smoothly in 72% overall yield of **11a**, formation of the monophosphine oxide **12a** was observed as a byproduct in 15% yield.

**Scheme 7:**
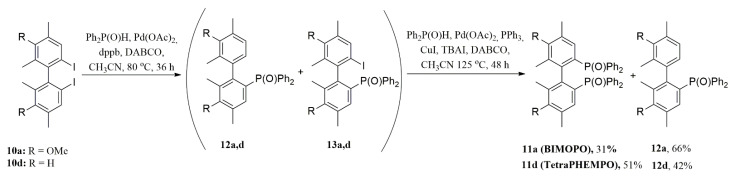
Alternative route to BIMOPO and TetraPHEMPO.

At the same time, we developed an alternative route of synthesis of **BIMOPO**, wherein diphenylphosphine oxide acted as a donor of the phosphorus moieties (Scheme 7). The synthesis based on the modified Hirao method was carried out in two steps. In the first step, the mixture of monophosphine oxided **12a** and **13a** was obtained under mild conditions, and then intermediates were subjected to the complete phosphorylation to **BIMOPO** in the second step. The **BIMOPO** yield (31%) was lower than that obtained by the classical iodide phosphorylation; however, it provided access to a number of valuable hard-to-reach products such as **12a** and **13a** and products of reactions with other readily available secondary phosphine oxides. In turn, with substrate **10d**, the reaction proceeded with full conversion and with moderate isolated yield of **TetraPHEMPO (11d)** (51%) and monophosphine oxide **12d** (42%).

Finally, the desired bisphosphines **BIMAP, BIMOP, BICLP** and **TetraPHEMP**, with full conversion were obtained by reduction of corresponding dioxides by trichlorosilane in the presence of tributylamine or by phenylsilane (**BIMAP**).

### 2.3. The Enantiomerically Pure Bisphosphines

Access to enantiomerically pure ligands could be provided by the separation of the racemic mixtures at several different stages of their synthesis. Thus, the resolution of racemic **DIDAB** would lead from one enantiomerically pure precursor to the entire series of optically pure or enantiomerically enriched ligands. Otherwise, the enantioseparation has to be applied individualy for each ligand. The enantioseparation of diphosphine oxides could be performed by crystallizating their diastereomeric salts with chiral non-racemic acids such as DBTA, naproxen [51]. The enantioseparation of diphosphines may go through the synthesis and crystallization of diastereomeric palladium complexes steps [40].

**Figure 2:**
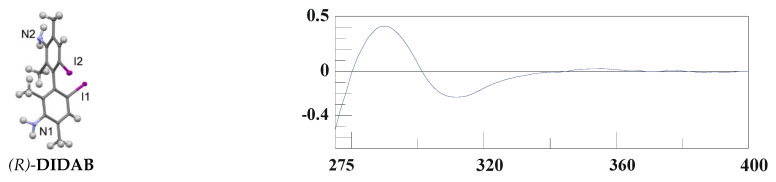
X-ray structure *(+)-(R)*-DIDAB and it CD spectrum in CH_3_CN.

The crucial intermediate **DIDAB (5)** was efficiently separated by crystallization using (−)-*O,O*′-dibenzoyl-L-tartaric acid ((-)-DBTA) as the resolving reagent. The optical resolution was carried out in hot chloroform to give 64% of 70% de salt DIDAB*DBTA, which was subjected to crystallization from methanol to yield pure (*+*)-**DIDAB** (in basic form) in 26% yield and enantiomeric excess above 99%, [*α*]_D_^20^ = +8.9 (*c =* 1, CH_2_Cl_2_). Interestingly, that the recrystallization of enantio enriched **DIDAB** from non-polar solvents, for example toluene or toluene/hexane, did not furnish the product with an optical purity greater than 96% ee. The absolute configuration of (*R*)-**DIDAB** was determined using X-ray analysis of crystals of basic (*R*)-**DIDAB,** which grew from acetonitrile solution (Figure 2). It was additionally confirmed by circular dichroism [52]. The enantiomeric composition of **DIDAB** was determined by means of ^1^H NMR spectroscopy: the spectrum of mixture 3.5 mg of **DIDAB** and 25 mg Eu(hfc)_3_ [53] was recorded and signals of corresponding to methyl groups and aromatic protons were integrated to calculate an enantiomeric excess according to the equation $\mathrm{ee},\%\;=\frac{A-A1}{A+A1}100\%$, where A and A1 are values of integrals of corresponding signals on spectra. The proper selection of the signals was confirmed in the experiment with racemic compound. The crystallographic analysis of monocrystalline (*R*)-**DIDAB** indicates that the aromatic rings are nearly perpendicular to each over with torsion between the phenyl rings being of 82.3(3)° which indicates significant repulsion of the bulky iodine atoms and methyl groups. (*S*)-**DIDAB** was isolated from the above crystallization residue after the enantio separation with (+)-DBTA in 31% yield and 95% ee, [*α*]_D_^20^ = −8.6 (*c =* 1, CH_2_Cl_2_).

From the enantiomerically enriched up to about 95% ee (*S*)-**DIDAB** the enantiomerically enriched diiodobiphenyl (*S*)-**10d** was obtained according to the Scheme 3. The (*S*)-**TetraPHEMPO** was obtained as described above (Scheme 7). The optical purity 90% ee of the resulting bisoxide was assessed by ^31^P NMR [52], In order to reach the complete optical purity, obtained (*S*)-**TetraPHEMPO** was recrystallized from methylcyclohexane. Subsequently, the enantiomerically pure (*S*)-**TetraPHEMP** was obtained by reduction of the corresponding bisoxide by trichlorosilane in toluene and tributylamine without racemization thereof. Similarly, enantiomerically pure (*R*)-**BIMOP** was obtained in phosphorylation reaction of (*R*)-**10a**, followed by deoxygenation of the bisphosphine dioxide formed.

**Figure 3:**
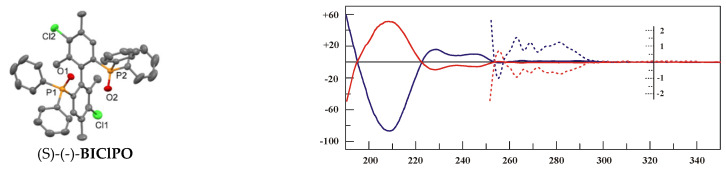
X-ray structure (S)-(-)-**BIClPO** (water molecule and hydrogen atoms were omitted for clarity) and the CD spectra of its enantiomers: (S)-(-)-**BIClPO** (>99% ee, blue), *(R)*-(+)-**BIClPO** (60% ee, red) recorded in CH_3_CN.

In turn, **BIClLPO** (**11c**) was an excellent example, when racemic bisoxide could be separated to enantiomerically pure forms using fractional crystallization of its salts with DBTA. From the solution of (*rac)*-**BICLPO** and (-)-DBTA in mixture of methylene chloride/carbon tetrachloride after the evaporation of a portion of CH_2_Cl_2_, the diastereomerically pure complex (*S*)-**BICLPO**∙(-)-DBTA in 40% yield of one enantiomer (ee > 99%, Figure 3) was crystalized. (*S*)-**BICLPO** was separated from its salt by extraction with methylene chloride from sodium carbonate aqueous solution and crystalized from a mixture of hexane/acetone. The enantiomeric purity of obtained bisphosphine dioxide was determined by NMR technique [52,54]. The ^1^H and ^31^P spectra of solution of mixture of **BIClPO** and mandelic acid in CDCl_3_ were recorded and the signals which correspond to aromatic hydrogen and phosphorus atoms were integrated to calculate an enantiomeric excess. The proper selection of the signals was confirmed in the experiment with racemic compound. The single crystal x-ray analysis confirmed the stereochemistry of this compound.

**Figure 4:**
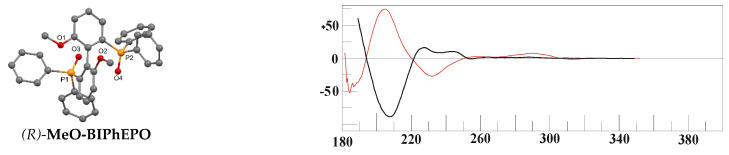
The CD spectra of *(S)-(-)-***BICLPO** (black) and *(R)-***MeO-BIPhEPO** (red) recorded in CH_3_CN.

The additional comparison of CD spectra of (*S)-(-)-***BICLPO** and *(R)-***MeO-BIPhEPO**, obtained from commercial *(R)-***MeO-BIPhEP** ligand [23] shows that these compounds adopt the opposite absolute configurations (Figure 4).

**Figure 5:**
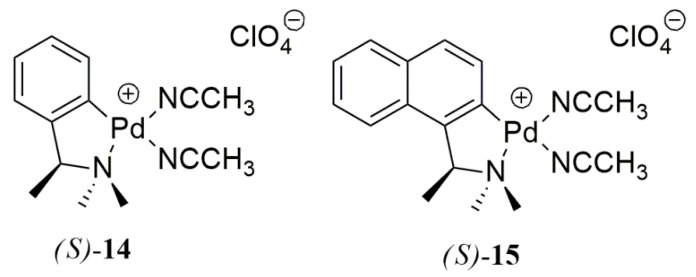
Palladium complexes **14** and **15** for the bisphosphines’ resolution.

Other than the cases described in Achiwa’s work [55,56], our attempts to separate **BIMOPO** (**9c**) enantiomers by fractional crystallization with addition of DBTA did not lead to satisfactory results. This was the case with further efforts to use chiral acids such as 2,3-di(phenylaminocarbonyl) tartaric acid [57], monodimethylamide DBTA, naproxen, mandelic acid or similar tested in a wide range of organic solvents. Surprisingly, resolution of racemic **BIMAPO** (**11b**), which contains amino groups at 5,5′-positions, also failed. Our further attempts focused on the application of chiral *C,N*-palladacycle complex which binds the bisphophine ligands. For this purpose, the palladium complexes **14** and **15** were used, which have been synthesized according to the literature data [58] (Figure 5).

**Scheme 8:**
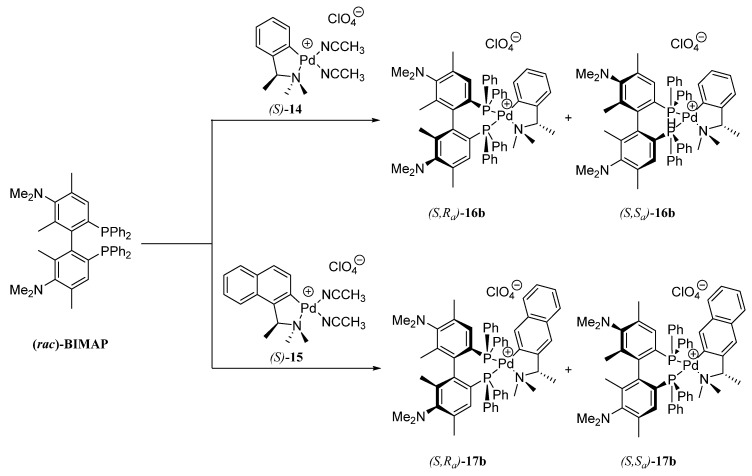
Synthesis of diastereomeric complexes **16** and **17**.

Racemic **BIMAP** ligand reacted quantitatively in mild conditions with both palladium complexes **14** and **15** giving mixtures of the corresponding diastereomeric products **16b, 17b,** respectively. It was suspected that such mixtures would crystallize and provide an access to diastereomerically pure products. The formation of diastereomeric adducts **16b** and **17b** was detected by NMR and mass spectroscopy. However, crystallization of the mixture of diastereomers **16b** did not allow to achieve the complete separation, even after several crystallizations the de was about 60%. In contrast, resolution of *(S,R_a_)*-**17b** and *(S,S_a_)*-**17b** complexes succeeded. The less soluble diastereoisomer *(S,R_a_)*-**17b** was isolated after several crystallizations from the mixture of ethanol/water and next hexane/ethyl acetate with de >98% (with respect to the total Pd) and with yield of 11% (Scheme 8).

**Scheme 9:**
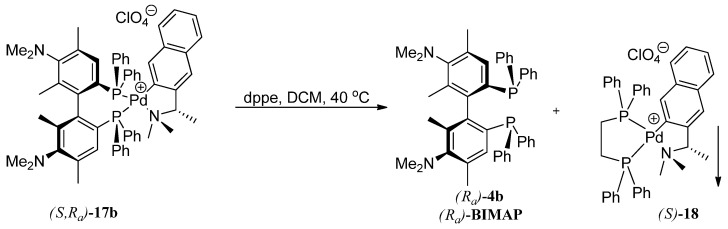
Synthesis of (*R_a_*)-**BIMAP**.

The enantiopure diphosphine *(R_a_)*-**BIMAP** was released from the *(S,R_a_)*-**17b** complex by replacing the ligand with **dppe** in methylene chloride as presented on Scheme 9. The optical purity was verified by the ^31^P NMR spectrum analysis of their corresponding complexes with the chiral ortho-palladium complex **14** as described in the literature [58,59].

**Figure 6:**
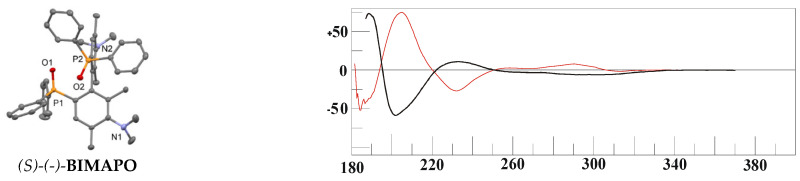
X-ray structure of *(S)-(-)-***BIMAPO** (solvent molecule and hydrogen atoms were omitted for clarity), its CD spectrum (black) as well the CD spectrum of *(R)-***MeO-BIPhEPO** (red).

An alternative route to enantiomerically pure ligands include the application of (semi)preparative chiral column chromatography. The racemic bisphosphine oxide **BIMAPO** (**11b**), was separated using Daicel Chiralpak AD column (250 × 10 × 10 um), which was eluted with hexane 88%, Et_2_NH (10–-3% in hexane) 10%, i-Pr 2% with a rate of 4 mL/min. Obtained enantiomers were then reduced with phenylsilane to the desired bisphosphine without any racemization in 87% yield. The enatiomers of **TetraPHEMPO** (**11d**), and **BIClPO** (**11c**) were separated similarly. The enantiomeric composition of all bisphosphine dioxides could be determined by means of chiral HPLC analysis or NMR spectroscopy [52,54]. The Figure 6 presents X-ray structure of enantiomerically pure (*S*)-(*-*)-**BIMAPO** which crystallizes as a solvate with benzene molecules, and the CD spectra of (*S*)-(*-*)-**BIMAPO** and *(R)-***MeO-BIPhEPO** used as a reference compound with known opposite to **BIMAPO** absolute configuration of biaryl core.

All the ligands obtained in chromatographic approach exhibited the enantiomeric purity over 99% ee. Nevertheless, from the practical point of view this method is not perfect since expensive chiral columns have to be used.

### 2.4. The Other Derivatives

**Scheme 10:**
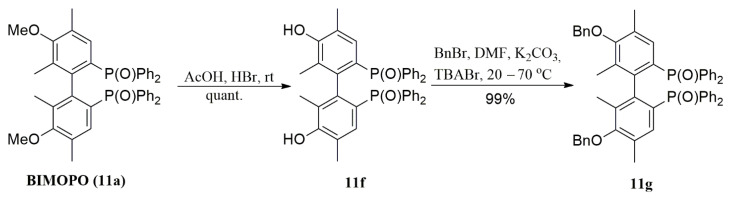
Synthesis of demethylated **BIMOPO** and its benzyl derivative.

It is important that the functional groups which are present in the ligands’ structures may allow for the introduction of different modifications providing the ligands with special properties such as solubility in water or nonpolar solvents or affinity to the solid supports without the influence on the ligand stereometry. At the same time, the electronic properties of the new ligands will be the same as those in the original ligand. This opportunity was presented on an example of modification of **BIMOPO** on methoxy groups (see Scheme 10).

For example, the oxygen atoms were deprotected in reaction with hydrogen bromide solution and the substituted biphenol **11f** obtained in quantitative yields was subjected to the alkylation reaction to obtain bisbenzylic derivative **11g** in excellent yield. The same protocol could be applied to introduce long-chain aliphatic substituents, polyether-chain substituents and some other substituents bearing basic and acidic functions.

The utilization of obtained ligands in asymmetric catalytic reactions as well as their special modifications, will be reported in a due time.

### 2.5. Cytotoxicity Assay

Some chiral biaryls (e.g., colchicine, allocolchicine, steganacin, rhazinilam) are known because of their biological activity, but in the majority of cases of biaryl compounds only those that are natural or synthetic (with the structures inspired by nature) are expected to be active and are therefore carefully assessed. On the other hand, the ligand, used in the chemical synthesis to form catalysts, could contaminate the products of the reactions and industrial or laboratory places. Surprisingly, this important issue is only rarely discussed, but must be taken into consideration during the designing of the synthesis of biologically active compounds e.g., medicines. Access to a small library of unnatural compounds based on generally common structural biaryl motif makes it possible to verify whether simple biaryls and triaryl phosphine oxides, commonly considered as biologically neutral, are safe.

**Figure 7:**
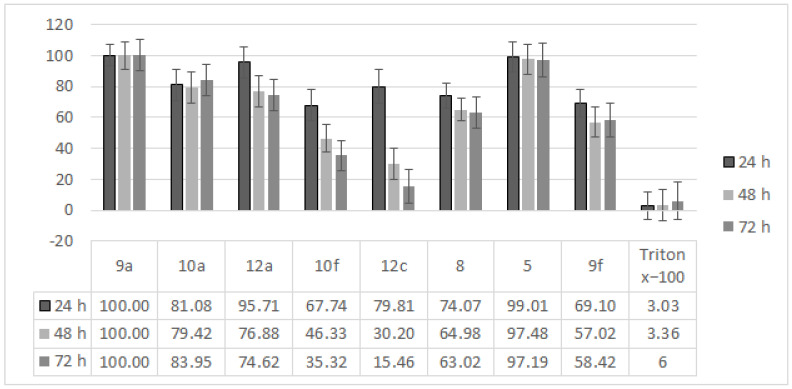
Cell viability (%) of normal human dermal fibroblasts (HDF) exposed to the tested compounds at a concentration of 200 µM assessed with the use of an MTT assay.

The biological activity of the compounds was determined at the highest possible concentration achieved in the cellular test conditions. The studied compounds showed various effects on human dermal fibroblasts. In the group of eight more soluble compounds tested at a concentration of 200 µM (see Figure 7), there were those that showed no cytotoxic effect (the maximum decrease in viability was about 3%) after 72 h of incubation (**9a** and **5**), others whose cytotoxic effect increased depending on the incubation time (**12a, 10f, 12c, 8, 9f**) while for compound **10a**, the effect appeared after 24 h and remained at a constant level for the next 48 h of exposure. The strongest cytotoxic effect within this group was shown by compound **12c**, leading to a decrease in cell viability up to 15.46% after 72 h of incubation.

**Figure 8:**
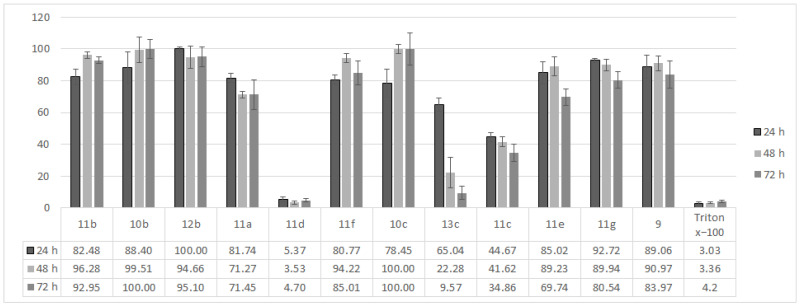
Cell viability (%) of normal human dermal fibroblasts (HDF) exposed to the tested compounds at a concentration of 50 µM assessed with a use of MTT assay.

In the second set of poorly soluble compounds, tested at a concentration of 50 µM (see Figure 8), one compound (**12b)** showed a slight cytotoxic effect on human cells (5% decrease in cell viability), some, despite initially demonstrated effectiveness, weakened with increasing exposure time (**11b, 10b, 11f, 10c**), and some compounds’ activity increased in a time dependent manner (**11a, 13c, 11c, 11e, 9a, 9**). Compound **11d** exhibited the highest toxicity, which after 24 h of incubation led to a decrease in cell viability by about 95%, and such effect remained constant up to 72 h of exposure, therefore it was found to be the most cytotoxic among all of the tested agents.

## 3. Materials and Methods

### 3.1. General Information

The reagents were purchased from commercial suppliers and used without further purification. Solvents were dried and distilled under argon before use. All of the reactions involving formation and further conversions of phosphines were carried out under argon atmosphere. Nuclear magnetic resonance (NMR) spectra were recorded on a Bruker AV300 (^1^H 300 MHz, ^31^P 121.5 MHz, ^13^C NMR 75 MHz) and Bruker AV500 (^1^H 500 MHz, ^31^P 202 MHz, ^13^C NMR 126 MHz) spectrometers (Bruker; Billerica, MA, USA). All spectra were recorded in CDCl_3_ solutions, unless mentioned otherwise, and the chemical shifts (δ) are expressed in ppm using internal reference to TMS and external reference to 85% H_3_PO_4_ in D_2_O for ^31^P. Coupling constants (J) are given in Hz. The abbreviations of signal patterns are as follows: *s*-singlet, *d*-doublet, *t*-triplet, *q*-quartet, *m*-multiplet, *b*-broad, and *i*-intensive. The IR spectra were recorded in KBr pallets and with ATR module on the Nicolet 8700A FTIR-ATR spectrometer: wave numbers are in cm-1. All separations and purifications by column chromatography were conducted by using Merck Silica gel 60 (230–400 mesh), unless noted otherwise. The X-ray data were collected at Nonius Kappa-CCD diffractometer using the MoKα = 0.71073 Å wavelength at 150 K for **DIDAB** and at room temperature for all other compounds. The structures were solved by direct methods (SHELXS) and refined by the full-matrix least-squares method based on *F*^2^ [60]. Hydrogen atoms were placed at calculated positions. The water molecule in **BIClPO** occupies a special position at 2-fold axis. Benzene molecule in **BIMAPO** was refined isotropically because of positional disorder. The quality of the X-ray measurement for *(S,R_a_)*-**17b** was not satisfactory for a full structure refinement. Only the symmetry, unit cell parameters and initial model of the molecule was obtained to confirm the molecular structure. All the details from data collecting and structure refinement are presented in Supplementary Materials in Tables S1–S4. The HDF1 (human dermal fibroblasts) cell line was obtained from ATCC. Cells were cultured in DMEM (Dulbecco’s Modified Eagle Medium, high glucose)+ GlutaMAX supplemented with penicillin (100 U/mL), streptomycin (100 U/mL) and 10% heat-inactivated FBS. Cells were maintained in a humidified atmosphere at 37 °C and 5% CO_2_ and passaged twice before performing an experiment.

### 3.2. Crystal Data

#### 3.2.1. Crystal Data for DIDAB

C_16_H_18_I_2_N_2_ (M =492.12 g/mol): tetragonal crystal system, space group P4_3_ (no. 78), a = 9.42900(10) Å, *c* = 19.2620(2) Å, V = 1712.51(3) Å^3^, Z = 4, T = 150(2) K, μ(MoKα) = 3.666 mm^−1^, Dcalc = 1.909 g/cm^3^, 3920 reflections measured (6.04° ≤ 2Θ ≤ 54.98°), 3920 unique (R_int_ = 0.0105, R_sigma_ = 0.0197), which were used in all calculations. The final R_1_ was 0.0193 (> 2sigma(I)) and wR_2_ was 0.0463 (all data).

#### 3.2.2. Crystal Data for BIMAPO

C_50_H_52_N_2_O_2_P_2_ (M =774.88 g/mol): orthorhombic crystal system, space group P2_1_2_1_2_1_ (no. 19), a = 13.0850(2) Å, b = 18.1300(3) Å, *c* = 18.7050(3) Å, V = 4437.41(12) Å^3^, Z = 4, T = 293(2) K, μ(MoKα) = 0.138 mm^−1^, Dcalc = 1.160 g/cm^3^, 10165 reflections measured (4.9° ≤ 2Θ ≤ 54.96°), 10165 unique (R_int_ = 0.0176, R_sigma_ = 0.0267), which were used in all calculations. The final R_1_ was 0.0493 (>2sigma(I)) and wR_2_ was 0.1381 (all data).

#### 3.2.3. Crystal Data for BICLPO

C_80_H_70_Cl_4_O_5_P_4_ (M =1377.04 g/mol): trigonal crystal system, space group P3_2_21 (no. 154), a = 13.13700(10) Å, *c* = 34.9290(3) Å, V = 5220.46(9) Å^3^, Z = 3, T = 293(2) K, μ(MoKα) = 0.315 mm^−1^, Dcalc = 1.314 g/cm^3^, 15710 reflections measured (4.28° ≤ 2Θ ≤ 54.94°), 7960 unique (R_int_ = 0.0279, R_sigma_ = 0.0396), which were used in all calculations. The final R_1_ was 0.0577 (>2sigma(I)) and wR_2_ was 0.1218 (all data).

#### 3.2.4. Crystal Data for (S,R_a_)-17b

C_58_H_62_ClN_3_O_4_P_2_Pd (M =1068.95 g/mol): monoclinic crystal system, space group C222_1_ (no. 20), a = 22.413(4) Å, b = 23.859(5) Å, *c* = 20.922(4) Å, V = 11188(4) Å^3^, Z = 4, T = 293(2) K, μ(MoKα) = 0.482 mm^−1^, Dcalc = 1.269 g/cm^3^, 6741 reflections measured (4.62° ≤ 2Θ ≤ 27.36°), 1719 unique (R_int_ = 0.1375), which were used in all calculations. The final R_1_ was 0.1018 (>2sigma(I)) and wR_2_ was 0.2342 (all data).

### 3.3. Synthesis

#### 3.3.1. Synthesis of 1-Iodo-2,4-dimethyl-3-nitrobenzene (7)

To prepare iodine reagent solution, solid KIO_3_ (16 g, 0.07 mol) was added in small portions over 45 min to the stirred solution of powdered iodine (120 g, 0.47 mol) in 95% H_2_SO_4_ (400 mL). The mixture was stirred for another 3 h at room temperature to fully dissolve the iodine. 1,3-dimethyl-1,2-nitrobenzene (**6**, 50 g, 0.33 mol) was dissolved and cooled to 0 °C concentrated H_2_SO_4_ (600 mL) in a two-necked round bottom flask equipped with a stirring bar and cooled into an ice bath. Next, the iodine reagent solution was added dropwise over the period of 2 h to the solution of 1,3-dimethyl-1,2-nitrobenzene. The reaction temperature was kept between 10–15 °C. After 1 h of continuous stirring the precipitated iodine was filtered off and the dark brown solution poured onto crushed ice. The resulting precipitate was filtered off, washed with water (20 × 200 mL), 1 M aqueous NaHCO_3_ (20 × 200 mL) and dissolved in warm chloroform (550 mL). Chloroform solutions were combined and a 3% solution of NaHCO_3_ in saturated aqueous solution of Na_2_S_2_O_3_ was added portion wise to complete discoloration of both phases. The organic phase was separated, washed with 1% aqueous NaHCO_3_ (100 mL), H_2_O (100 mL) and dried over MgSO_4_. The solvent was removed, and the resulting crude product purified by fractional distillation under reduced pressure. The collected fraction of 110–130 °C at 0.5 Torr contained a product with purity greater than 98%. Yield 90 g (95%). Yellow solid, mp = 70–73 °C (non-recrystallized); lit. 68–70 °C [61]. IR (cm^−1^): 2985, 2929, 2877, 2728, 1896, 1757, 1622, 1518, 1449, 1365, 1254, 1211, 1150, 1090, 1030, 999, 932, 856, 810, 738, 615, 598, 517. ^1^H NMR: δ = 2.23 (3 H, s, CH_3_), 2.38 (3 H, s, CH_3_), 6.85 (1 H, d, *J =* 8.2 Hz, CH), 7.80 (1 H, d, *J =* 8.2 Hz, CH). ^13^C NMR: δ = 17.0 (CH_3_), 23.4 (CH_3_), 98.3 (CI), 129.3 (C-*CH_3_*), 130.0 (CH), 132.2 (C-*CH_3_*), 140.3 (CH), 151.6 (CNO_2_). Anal. Calcd. for C_8_H_8_NO_2_I (277.06) C 34.68, H 2.91, N 5.06; Found: C 34.67, H 2.95, N 5.12.

#### 3.3.2. Synthesis of 2,2′, 4,4′-Tetramethyl-3,3′-dinitrobiphenyl (8)

To 1% Cu(NO_3_)_2_ solution in 1 M aqueous H_2_SO_4_ (500 mL) the copper powder (120 g, 2 mol) was added and mixed for 6 h. Then, the activated copper was filtered off, washed with water (100 mL) followed by acetone 30 × 100 mL and dried under reduced pressure. To a solution of 1-iodo-2,4-dimethyl-3-nitrobenzene (7) (125 g, 0.45 mol) in 200 mL DMF, activated copper powder was added, and then the mixture was boiled for 24 h, cooled to room temperature, and filtered by Celite. The solvents were evaporated, and the remaining crude product was recrystallized from a toluene/hexane mixture. Yield 57 g (85%). Yellow solid, mp = 139–140 °C. IR (cm^−1^): 2967, 2931, 2884, 1938, 1918, 1611, 1527, 1450, 1367, 1210, 1190, 1155, 1036, 994, 876, 857, 844, 831, 775, 743, 589. ^1^H NMR: δ = 1.99 (6 H, s, CH_3_), 2.35 (6 H, s, CH_3_), 7.11 (2 H, d, *J =* 7.8 Hz, CH), 7.20 (2 H, d, *J =* 7.8 Hz, CH). ^13^C NMR: δ = 14.7 (CH_3_), 17.2 (CH_3_), 127.4 (C-*CH_3_*), 128.7 (CH), 129.0 (C-*CH_3_*), 130.9 (CH), 139.0 (CC) 152.7 (CNO_2_). Anal. Calcd. for C_16_H_16_N_2_O_4_ (300.32) C 63.99, H 5.37, N 9.33; Found. C 63.96, H 5.44, N 9.11.

#### 3.3.3. Synthesis of 2,2′,4,4′-Tetramethyl-3,3′-diaminobiphenyl (9)

2,2′,4,4′-tetramethyl-3,3′-dinitrobiphenyl (**8**) (30 g, 0.1 mol) was dissolved in THF (50 mL) and then 150 mL of methanol was added. The resulting solution was placed in a stainless steel autoclave and loaded with 10% Pd/C (1 g). The autoclave was filled with hydrogen (150 atm, 25 °C) and placed in a shaker for 6 h at 150 °C. After cooling down to room temperature, the hydrogen pressure in the autoclave was supplemented to 150 atm and the autoclave was again heated at 150 °C for another 6 h. Upon cooling, the hydrogen from the autoclave was slowly released and the catalyst filtered off by celite. Evaporation of the solvent afforded a product with a purity of 98-99% and no further purification was necessary. Yield 37 g (99%). Light yellowish crystals, mp = 144–145 °C (crystallized from toluene/hexane). IR (cm^−1^): 3446, 3417, 3361, 3234, 3021, 2962, 2924, 2891, 2853, 2726, 2590, 1868, 1740, 1628, 1571, 1479, 1460, 1418, 1293, 1271, 1207, 1151, 1124, 1077, 990, 822, 805, 761, 717, 520. ^1^H NMR: δ = 1.89 (6 H, s, CH_3_), 2.22 (6 H, s, CH_3_), 3.63 (4 H, br.s, NH_2_), 6.53 (2 H, d, *J =* 7.6 Hz, CH), 6.94 (2 H, d, *J =* 7.6 Hz, CH). ^13^C NMR: δ = 14.1 (CH_3_), 17.7 (CH_3_), 119.7 (CH), 120.0 (C-*CH_3_*), 120.2 (C-*CH_3_*), 127.2 (CH), 141.0 (CNH_2_) 142.5 (CC). Anal.calcd. for C_16_H_20_N_2_ (240.35) C 79.96, H 8.39, N 11.66; Found: C 79.87, H 8.46, N 11.57.

#### 3.3.4. Synthesis of 4,4′, 6,6′-Tetramethyl-5,5′-diamino-2,2′-diiodobiphenyl—DIDAB (5) 

To a solution of 2,2′,4,4′-tetramethyl-3,3′-diaminobiphenyl **(9)** (10 g, 0.042 mol) in mixture of CH_2_Cl_2_ (300 mL) and MeOH (130 mL), BTMA∙ICl_2_ (30 g 0.086 mol) prepared as reported [47], and CaCO_3_ (20 g, 0.2 mol) were added. The mixture was stirred for 48 h at room temperature. Excess of CaCO_3_ was filtered off and the resulting filtrate concentrated and dissolved in CH_2_Cl_2_ (100 mL). The resulting solution was washed with NaHCO_3_ (3 × 50 mL) and water (2 × 50 mL) and dried over MgSO_4_. Next, the solvent was evaporated, and the crude product purified by column chromatography (acetone/hexane 1/3) followed by recrystallization from toluene. Yield: 15g (75%). Orange crystalline powder, mp = 218–220 °C. IR (cm^−1^): 3474, 3392, 2971, 2910, 2853, 2727, 1726, 1614, 1555, 1455, 1414, 1301, 1281, 1222, 1174, 993, 866, 849, 732, 510. ^1^H NMR: δ = 1.84 (6 H, s, CH_3_), 2.19 (6 H, s, CH_3_), 3.67 (4 H, br s, NH_2_), 7. 51 (2 H, s, CH). ^13^C NMR: δ = 15.5 (CH_3_), 17.4 (CH_3_), 86.6 (CI), 121.5 (C-*CH_3_*), 123.4 (C-*CH_3_*), 137.3 (CH), 143.1 (CNH_2_), 146.2 (CC). MS (ES): m/z (%) = 493 (100, M+H^+^), 515 (5, M+Na^+^). MS HR (ES): m/z = calcd. 492.9632 (M+H^+^, C_16_H_19_N_2_I_2_), found 492.9650 (M+H^+^, C_16_H_19_N_2_I_2_). Anal. calcd. for C_16_H_18_N_2_I_2_ (492.14) C 39.05, H 3.69, I 51.57, N 5.69; found: C 39.06, H 3.75, I 51.42, N 5.65.

#### 3.3.5. Separation of DIDAB Enantiomers

To the boiling solution of *(-)*-DBTA (8.2 g, 0.023 mol) in CHCl_3_ (500 mL), a solution of racemic **DIDAB** (4.5 g, 0.049 mol) in CHCl_3_ (30 mL) was added dropwise. After addition of whole amount of diamine, the mixture was heated for 1 h and then the solution was left without stirring at room temperature. Salt (DBTA∙DIDAB = 1:1, 5 g (64%), 70% ee) slowly precipitated in duration of 72 h. Further proceedings were carried out in two variants.

##### Variant 1

The precipitate was filtered off, dissolved in hot MeOH and slowly cooled to room temperature. Pure *(R)*-**DIDAB** crystallized in the form of orange crystals within 72 h. Yield 1.2 g (26%), 99% ee. Decomposition temperature: 220–225 °C. *(R)-***DIDAB**, [*α*]_D_^20^ = +8.9 (*c =* 1, CH_2_Cl_2_). CD (3.4∙10^−4^ M, CH_2_Cl_2_): −9 (232), −8 (246), +1 (285), −0.5 (306).

##### Variant 2

The precipitate was filtered off and mixed with 1 M NaOH (50 mL) and CH_2_Cl_2_ (100 mL) until fully dissolved. The organic phase was separated, washed with 1 M NaOH (50 mL), and dried over MgSO_4_. After evaporation of the solvent, the amine was crystallized from CHCl_3_ (150 mL) with *(-)*-DBTA (3 g, 0.008 mol) as described in the Variant 1. *(R)*-**DIDAB** was isolated from the resulting salt (DBTA∙**DIDAB** = 1:1, 3.3 g) and recrystallized from toluene/hexane. Yield 1.3 g (31%), 95% ee. [*α*]_D_^20^ = +8.5 (*c* = 1, CH_2_Cl_2_). *(S)*-enriched **DIDAB** was isolated from liquid residual and then *(S)*–enantiomer was resolved with *(+)*-DBTA according to the procedure described above. Yield 1.7 g (42%), 96% ee. [*α*]_D_^20^ = −8.6 (*c* = 1, CH_2_Cl_2_).

#### 3.3.6. Synthesis of *N,N,N’,N’*-4,4′,6,6′-Octamethyl-5,5′-diamino-2,2′-diiodobiphenyl (10b) and (*R*)-10b

A 37% aqueous formaldehyde solution (12 mL) was slowly added to a cooled to −10 °C mixture of 3 M aqueous H_2_SO_4_ (40 mL) and THF (50 mL). To the resulting solution, a slurry of **DIDAB** (4 g, 8 mmol) and NaBH_4_ (4.8 g, 129 mmol) in THF (200 mL) was added over 1 h in small portions. The reaction temperature was maintained in range of −5 to 0 °C. Next, the reaction was stirred for 18 h at room temperature. After this time THF was evaporated and CH_2_Cl_2_ (100 mL) was added. To the intensively stirring biphasic mixture, a 4 M aqueous NaOH was added dropwise to obtain the pH of the aqueous phase about 14. The organic phase was separated, washed with water (20 mL) and dried over MgSO_4_. After evaporation of the solvent, the product was isolated by column chromatography (hexane/ethyl acetate: 160/1) and recrystallized from hexane. Yield 3.8 g (92%). Colorless crystals, mp = 140–141 °C (crystallized from hexane). IR (cm^−1^): 2959, 2915, 2825, 2780, 1714, 1570, 1539, 1443, 1391, 1315, 1220, 1165, 1127, 1058, 1021, 987, 951, 859, 811, 515. ^1^H NMR: δ = 1.94 (6 H, s, CH_3_), 2.29 (6 H, s, CH_3_), 2.82 (12 H, s, NCH_3_), 7.60 (2 H, s, CH). ^13^C NMR: δ = 17.3 (CH_3_), 18.9 (CH_3_), 42.5 (NCH_3_), 96.2 (CI), 137.0 (C-CH_3_), 138.4 (C-CH_3_), 138.4 (CH), 147.0 (CNCH_3_), 150.5 (CC). MS (EI): m/z (%) = 548 (100, M^+^), 391 (25), 279 (20). MS HR (EI): m/z = Calcd. 548.01855 (M^+^, C_20_H_26_N_2_I_2_), found 548.01782 (M^+^, C_20_H_26_N_2_I_2_). Anal. calcd. for C_20_H_26_N_2_I_2_ (548.25) C 43.82, H 4.78, I 46.29, N 5.11; found C 43.83, H 4.76, I 46.36, N 5.11. (*R*)-*N,N,N’,N’*-4,4′,6,6′-octamethyl-5,5′-diamino-2,2′-diiodobiphenyl **((*R*)-10b):** [α]_D_^20^ = +36.9 (*c* = 0.9, CH_2_Cl_2_), 80% ee. CD (3.4∙10^−4^ M, CH_2_Cl_2_): −1 (233), +2 (252), +1.6 (272).

#### 3.3.7. Synthesis of *N,N,N’,N’*-Tetrabutyl-4,4′, 6,6′-tetramethyl-5,5′-dibutylamino-2,2′-diiodobiphenyl (10e)

To a cooled to −35 °C THF (50 mL), 98% H_2_SO_4_ (3 g, 30 mmol) in 5 mL of water was added followed by butyric aldehyde (3.38 g, 45 mmol). Next, the resulting solution a slurry of **DIDAB** (2.5 g, 5 mmol) and NaBH_4_ (0.83 g, 22.5 mmol) in THF (50 mL) was added over 1 h in small portions. The reaction temperature was maintained at −35 to −20 °C. Upon completion of the addition, the reaction mixture was stirred for 1 h, then the cooling bath was removed and the reaction mixture was stirred for two more hours at room temperature. Subsequently, 1 M aqueous NaOH was added dropwise to adjust pH to around 12, then the product was extracted with hexane (2 × 20 mL). The combined organic phases were washed with water (20 mL) and dried over MgSO_4_. After evaporation of the solvent, the product was isolated by column chromatography (hexane). Yield 3.1 g (89%). Transparent, rapidly darkening liquid. IR (film, cm^−1^): 2956, 2929, 2871, 2860, 2730, 1782, 1551, 1456, 1421, 1377, 1282, 1241, 1204, 1132, 1098, 1028, 989, 900, 864, 814, 733, 515. ^1^H NMR: δ = 0.84–0.94 (12 H, m, CH_3_), 1.22–1.30 (8 H, m, CH_2_), 1.36–1.44 (8 H, m, CH_2_), 1.94 (6 H, s, CH_3_), 2.29 (6 H, s, CH_3_), 2.95–3.05 (8 H, m, CH_2_N), 7.61 (2 H, s, CH). MS (EI): m/z (%) = 561 (20), 617 (50), 673 (100), 716 (20, M^+^). MS HR (EI): m/z = Calcd. 716.20635 (M^+^, C_32_H_50_N_2_I_2_), found 716.20487 (M^+^, C_32_H_50_N_2_I_2_).

#### 3.3.8. Synthesis of 4,4′,6,6′-Tetramethyl-5,5′-dimethoxy-2,2′-diiodobiphenyl (10a) 

To a cooled down to −10 °C solution of **DIDAB** (33 g, 0.06 mol) in dry methanol (300 mL), 98% H_2_SO_4_ (35 g, 0.35 mol) was added. The solution was stirred at that temperature for 30 min and cooled down to −20 °C. Next, isoamyl nitrite (17 g, 0.15 mol) was added and the solution was stirred at given temperature for 1 h and at −5 °C for 2 h. The temperature was increased up to 0 °C and NH_2_SO_3_H (3 g, 0.03 mol) was added in two portions over 40 min. The temperature was elevated up to +10 °C and palladium acetate (80 mg, 0.36 mmol) was added. The mixture was stirred for 15 min at +10 °C and 10 h at the reflux conditions. The solvent was evaporated off and the residual poured on 200 g of crushed ice. The product was extracted with DCM, washed with water, dried with MgSO_4_. Drying agent was removed by filtration, solvent evaporated under the reduced pressure and the residue was dissolved in dry 300 mL of CH_3_CN. To the solution 52 g of anhydrous K_2_CO_3_, 1 g of TBABr and 21 g of (MeO)_2_SO_2_ were added. The mixture was stirred at ambient temperature for 60 h. Insoluble salts were filtered off, solvents were evaporated under the reduced pressure, the residual was dissolved in 200 mL of DCM, washed with 1 M hydrochloric acid 2 × 50 mL) and 1 M sodium hydroxide (4 × 50 mL). Organic phase was separated, washed with water and dried over the MgSO_4_. The last was filtered off, solvent was evaporated and residual purified by column chromatography (hexane/ethyl acetate: 160/1). The crude product could be alternatively crystallized from hexane at −20 °C. Yield 24 g (68%). Colorless crystals, mp = 116–118 °C (crystallized from hexane). IR (cm^−1^): 2986, 2933, 2851, 1585, 1547, 1456, 1411, 1389, 1279, 1261, 1209, 1175, 1152, 1087, 1000, 831, 722, 510. ^1^H NMR: δ = 1.93 (6 H, s, CH_3_), 2.30 (6 H, s, CH_3_), 3.72 (6 H, s, OCH_3_), 7.64 (2 H, s, CH). ^13^C NMR: δ = 14.5 (CH_3_), 15.9 (CH_3_), 60.0 (OCH_3_), 94.3 (CI), 131.0 (C-CH_3_), 132.6 (C-CH_3_), 138.5 (CH), 146.7 (CC), 157.7 (C-OCH_3_). MS (EI): m/z (%) = 522 (100, M^+^), 395 (25), 253 (50). HRMS (EI): m/z = Calcd. 521.95,528 (M^+^, C_18_H_20_O_2_I_2_), found 521.95754 (M^+^, C_18_H_20_O_2_I_2_). Anal. calcd. for C_18_H_20_O_2_I_2_ (522.17) C 41.40, H 3.86, I 48.61; found C 41.33, H 3.65, I 48.75.

Alternatively, 4,4′,6,6′-tetramethyl-5,5′-dimethoxy-2,2′-diiodobiphenyl could be obtained from **9** after the deaminomethoxylation, as described above for the case of **10a,** where the 2,2′,4,4′-tetramethyl-3,3′-dimethoxybiphenyl was obtained in 80% yield as a white powder with mp = 86–67 °C after a crystallized from hexane. IR (cm^−1^): 3023, 2985, 2951, 2934, 2922, 2856, 2823, 2727, 2006, 1895, 1767, 1602, 1565, 1475, 1446,1396, 1300, 1263, 1214, 1167, 1135, 1083, 1009, 822, 813, 672, 513. ^1^H NMR: δ = 1.98 (6 H, s, CH_3_), 2.33 (6 H, s, CH_3_), 3, 75 (6 H, s, OCH_3_), 6.78 (2 H, d, J = 7.7 Hz, CH), 7.03 (2 H, d, J = 7.7 Hz, CH). ^13^C NMR: δ = 13.1 (CH_3_), 16.1 (CH_3_), 59.7 (OCH_3_), 125.0 (CH), 128.0 (CH), 129.2 (C-CH_3_), 129.4 (C-CH_3_), 141.0 (CC) 157.0 (COCH_3_). Anal. calcd. for C_18_H_22_O_2_ (270.37) C 79.96, H 8.20; found: C 79.86, H 8.45. The iodination of 2,2′,4,4′-tetramethyl-3,3′-dimethoxybiphenyl was realized as follows. To a solution of anhydrous ZnCl_2_ (28 g, 0.2 mol) in CH_3_COOH (150 mL), obtained 2,2′,4,4′-tetramethyl-3,3′-dimethoxybiphenyl (10 g, 37 mmol) and a solution of BTMA∙ICl_2_ (27 g, 77 mmol) in CH_3_COOH (150 mL) were slowly added. The resulting solution was stirred for 2 h at room temperature and another 60 h at 35 °C. Then, cold water (300 mL) was added to the reaction mixture and the product was extracted with hexane (5 × 75 mL). The combined organic phases were washed with saturated NaHCO_3_ (2 × 50 mL) and dried over MgSO_4_. The crude product was crystallized from hexane at −20 °C. Yield 15g (77%).

#### 3.3.9. Synthesis of 4,4′,6,6′-Tetramethyl-2,2′-diiodobiphenyl (10d) and (*S*)-10d

To prepared solution of **DIDAB** (0.6 g, 1.3 mmol) in THF (25 mL), 3 M aqueous H_2_SO_4_ (2.2 mL) was added and the mixture was cooled down to −10 °C followed by addition of NaNO_2_ (0.2 g, 2.9 mmol) in H_2_O (1 mL) and stirred for 45 min. Next, a solution of NH_2_SO_3_H (0.1 g, 1 mmol) in water (2 mL) was added in three portions and mixture was stirred 15 min more at 0 °C. Then 50% aqueous H_3_PO_2_ (5 mL) and Cu_2_O (50 mg) were added sequentially. The mixture thus obtained was stirred for 18 h at room temperature and then for 5 h at 60 °C. Next, THF was evaporated and water (50 mL) was added, the product was extracted with benzene (50 mL), dried and purified by column chromatography (hexane/ethyl acetate: 160/1). Yield 500 mg (89%). Colorless crystals, mp = 116–118 °C (crystallized from hexane). IR (cm^−1^): 3008, 2944, 2912, 2852, 1774, 1736, 1598, 1540, 1454, 1437, 1373, 1242, 1206, 1147, 1120, 1040, 1008, 985, 849, 782, 736, 589, 542. ^1^H NMR: δ = 1.97 (6 H, s, CH_3_), 2.33 (6 H, s, CH_3_), 7.07 (2 H, s, CH), 7.63 (2 H, s, CH). ^13^C NMR: δ = 20.7 (CH_3_), 21.4 (CH_3_), 101.0 (CI), 131.0 (CH), 137.2 (C-*CH_3_*), 137.2 (CH), 139.2 (C-*CH_3_*), 144.5 (CC). MS (EI): m/z (%) = 462 (100, M^+^), 335 (50), 208 (35), 193 (60). HRMS (EI): m/z = Calcd. 461.93415 (M^+^, C_16_H_16_I_2_), found 461.93385 (M^+^, C_16_H_16_I_2_). Anal. calcd. for C_16_H_16_I_2_ (462.11) C 41.59, H 3.49, I 54.92; found C 41.57, H 3.43, I 55.09. (*S*)- 4,4′,6,6′-tetramethyl-2,2′-diiodobiphenyl **((*S*)-10d)**: [*α*]_D_^20^ = +28.5 (*c* = 2.4, CDCl_3_). CD (1.3∙10^−4^ M, CH_3_CN): +10 (195), −29 (208), +13 (233), −2 (252).

#### 3.3.10. Synthesis of 4,4′,6,6′-Tetramethyl-5,5′-dichloro-2,2′-diiodobiphenyl (10c)

Into a stirred suspension of **DIDAB** powder (3 g, 6 mmol) in concentrated HCl (15 mL) water (10 mL) was added and the whole was cooled to −15 °C. Next, a solution of NaNO_2_ (1.14 g, 16 mmol) in 2 mL of water was added dropwise over the period of 30 min. After that time, a solution of NH_2_SO_3_H (0.7 g, 7.5 mmol) was added in several portions and the reaction mixture was stirred for 20 min. A catalyst solution was prepared as follows: copper(I) oxide (2 g, 14 mmol) mixed with concentrated HCl (5 mL) for 30 min and acetone (30 mL) and CuCl_2_ (50 mg) were added to the reaction with bisdiazonium salt. The reaction mixture was stirred for several hours at 0 °C and overnight at ambient temperature. Next, the reaction mixture was heated up to 70 °C for 2 h, what resulted in acetone evaporation. After cooling, the product was extracted with CH_2_Cl_2_ (50 mL). The organic phase was separated, washed with 1 M HCl and aqueous NaHCO_3_ and dried over MgSO_4_. After evaporation of the solvent, the product was isolated by column chromatography (hexane/ethyl acetate: 80/1). Yield 2.9 g (91%). Colorless crystals, mp = 153–154 °C (crystallized from hexane). IR (cm^−1^): 2974, 2950, 2918, 2851, 1737, 1577, 1532, 1444, 1378, 1245, 1169, 1146, 1054, 1032, 1011, 993, 868, 810, 702, 647, 464. ^1^H NMR: δ = 2.06 (6 H, s, CH_3_), 2.40 (6 H, s, CH_3_), 7.72 (2 H, s, CH). ^13^C NMR: δ = 19.2 (CH_3_), 20.6 (CH_3_), 97.8 (CI), 135.8 (C-Cl), 137.9 (C-*CH_3_*), 138.3 (C-*CH_3_*), 138.3 (CH), 146.4 (CC). MS (EI): m/z (%) = 530 (100, M^+^), 403 (60), 241 (90). MS HR (EI): m/z = Calcd. 529.85621 (M^+^, C_16_H_14_^35^Cl_2_I_2_), found 529.85815 (M^+^, C_16_H_14_^35^Cl_2_I_2_). Anal. calcd. for C_16_H_14_Cl_2_I_2_ (531.00) C 36.19, H 2.66, I 47.80; found C 36.32, H 2.77, I 47.79.

#### 3.3.11. Synthesis of 6,6′-Bis(Diphenylphosphoryl)-*N,N,N’,N’*,2,2′,4,4′-octamethylbiphenyl-3,3′-diamine (BIMAPO) (11b)

To a cooled to −70 °C solution of *N,N,N’,N’*-4,4′,6,6′-octamethyl-5,5′-diamino-2,2′-diiodobiphenyl **(10b)** (0.745 g, 1.36 mmol) in unhydrous Et_2_O (20 mL), a 1.3 M solution of *n*-BuLi in hexane (2.9 mmol) was slowly added. The reaction mixture was stirred for 1 h at −70 °C, then for next 1 h at −20 °C. Subsequently, the solutions of TMEDA (652 μL, 4.32 mmol) in Et_2_O (20 mL) and Ph_2_P(O)Cl (1.0 g, 4 mmol) in Et_2_O (5 mL) were added and resulting mixture stirred 4 h at −20 °C, overnight at room temperature, and then 4 h at 36 °C. The obtained precipitate was filtered off and dissolved in CH_2_Cl_2_ (100 mL), washed with 1 M aqueous NaOH (3 × 30 mL) and water (2 × 30 mL) and dried over MgSO_4_. After evaporation of the solvent, the residue was refluxed in hexane (50 mL) for 1 h. The crude product was filtered off from a hot solution and recrystallized from toluene/hexane. Yield 0.5 g (52%). White crystalline powder, mp > 250 °C. IR (cm^−1^): 3393, 3144, 3051, 2959, 2893, 2859, 2784, 1573, 1539, 1436, 1410, 1391, 1372, 1321, 1201, 1189, 1124, 1114, 1101, 1068, 996, 956, 880, 752, 699, 556, 511. ^31^P NMR: δ = 29.0. ^1^H NMR: δ = 1.32 (6 H, s, CH_3_), 2.16 (6 H, s, CH_3_), 2.70 (12 H, s, NCH_3_), 6.83 (2 H, d, J = 14.2 Hz, CH), 7.22–7.25 (4 H, m, Ph), 7.29–7.34 (2 H, m, Ph), 7.35–7.40 (4 H, m, Ph), 7.43–7.47 (2 H, m, Ph), 7.65–7.75 (8 H, m, Ph). ^13^C NMR: δ = 15.5 (s, CH_3_), 19.6 (s, CH_3_), 42.2 (s, NCH_3_), 127.4 (d, J = 107.4 Hz, CP), 127.8 (d, J = 12.6 Hz, C_o-_H), 127.9 (d, J = 12.6 Hz, C_o-_H), 130.4 (d, J = 2.5 Hz, C_p-_H), 130.7 (d, J = 2.4 Hz, C_p-_H), 132.3 (d, J = 8.9 Hz, C_m-_H), 132.5 (d, J = 9.8 Hz, C_m-_H), 133.6 (d, J = 13.5 Hz, CH), 134.2 (d, J = 14.2 Hz, C-*CH_3_*), 134.5 (d, J = 100.5 Hz, CP), 135.2 (d, J = 104.0 Hz, CP), 136.3 (d, J = 11.3 Hz, C-*CH_3_*), 142.7–142.8 (4 peaks, CC), 152.5 (d, J = 2.9 Hz, CN). MS (ES): m/z (%) = 697 (100, M + H^+^), 719 (10, M+Na^+^); MS HR (ES): m/z = calcd. 697.3107 (M+H^+^, C_44_H_47_N_2_O_2_P_2_), found. 697.3138 (M+H^+^, C_44_H_47_N_2_O_2_P_2_). Anal. calcd. for C_44_H_46_N_2_O_2_P_2_ (696.82) C 75.84, H 6.65, N 4.02; found. C 75.56, H 6.83, N 3.97.

#### 3.3.12. Synthesis of 6-(Diphenylphosphoryl)-*N,N,N’,N’*,2,2′,4,4′-octamethylbiphenyl-3,3′-diamine (12b) 

This compound was isolated by column chromatography (hexane/ethyl acetate/methanol 5/3/0.2) as a byproduct in **BIMAPO** synthesis. Yield 0.136 g (21%). White crystalline powder, mp = 180–181 °C (crystallized from benzene/hexane). IR (cm^−1^): 3056, 2960, 2918, 2860, 2831, 2783, 1963, 1894, 1819, 1769, 1681, 1537, 1437, 1323, 1192, 1121, 1101, 1064, 955, 887, 818, 751, 721, 696, 558, 527. ^31^P NMR: δ = 28.27. ^1^H NMR: δ = 1.56 (3 H, s, CH_3_), 1.72 (3 H, s, CH_3_), 2.09 (3 H, s, CH_3_), 2.18 (3 H, s, CH_3_), 2.59 (6 H, br s, CH_3_N), 2.77 (6 H, s, CH_3_N), 6.45 (1 H, d, J = 7.5 Hz, CH), 6.51 (1 H, d, J = 7.5 Hz, CH), 7.15–7.25 (5 H, m, CH + Ph), 7.27–7.32 (2 H, m, Ph), 7.40–7.50 (4 H, m, Ph). ^13^C NMR: δ = 15.8 (s, CH_3_), 16.0 (s, CH_3_), 19.0 (s, CH_3_), 19.5 (s, CH_3_), 42.4 (s, CH_3_), 127.1 (d, J = 105.8 Hz, CP), 127.3 (d, *J =* 24 Hz, CH), 127.7 (d, J = 11.9 Hz, C_o-_H), 130.6 (d, *J =* 2.8 Hz, C_p-_H), 130.8 (d, J = 2.7 Hz, C_p-_H), 131.5 (d, *J =* 9.5 Hz, C_m-_H), 131.8 (d, *J =* 9.2 Hz, C_m-_H), 133.5 (d, *J =* 12.7 Hz, C), 134.4 (d, *J =* 12.1 Hz, CH), 135.5 (s, CH), 135.9 (s, CH), 137.4-137.5 (4 peaks, CC), 145.1 (d, *J =* 9.7 Hz, C), 148.8 (s, C), 153.5 (d, *J =* 3.0 Hz, C). MS (ES): m/z (%) = 497 (100, M + H^+^). MS HR (ES): m/z = calcd. 497.2716 (M + H^+^, C_32_H_38_ON_2_P), found. 497.2739 (M + H^+^, C_32_H_38_ON_2_P).

#### 3.3.13. Synthesis of 6,6′-Bis(diphenylphosphoryl)-*N,N,N’,N’*,2,2′,4,4′-octamethylbiphenyl-3,3′-diamine ((*S*)-BIMAPO) ((*S*)-11b)

The compound was obtained via a semipreparative column chromatography using a DAICEL CHIRALPACK AD column (250 mm × 10 mm × 10 μm), Mobile phase: hexane: 0.01% Et_2_NH in hexane: *i*-PrOH (88:10:2), Flow rate: 4 mL/min. About 5 mg of racemic **BIMAPO** dissolved in 0.5 mL mixture of ethyl acetate/methylene chloride (1:1) was injected to the column. Fractions containing *(S)-***BIMAPO** enantiomer were collected in 20 to 40 min while fractions containing *(R)*-**BIMAPO** were collected in 50 to 80 min. Repeating the separation procedures furnished a necessary amount of enantiomerically enriched **BIMAPO** fractions. Optically pure products were obtained by additional crystallization from ethyl acetate. Yield *(S)*-**BIMAPO** 51 mg (68%), >99% ee; *(R)*-**BIMAPO** 45 mg (60%), >99% ee.

*(S)*-**BIMAPO**: White crystals, mp > 250 °C. [*α*]_D_^20^ = −162.2 (*c* = 1.0, CH_2_Cl_2_). CD (7.6 × 10^−5^ M, CH_3_CN): +82.5(188.0), −59.81 (201.0), +11.40 (232.0), −2.91 (262.5), −6.01 (300.0).

#### 3.3.14. Determination of Enantiomeric Composition BIMAPO (11b) 

The enantiomeric purity of obtained bisphosphine dioxide was determined by NMR technique: [52,54] the ^1^H and ^31^P spectra of solution of 2 mg of compounds and 10 mg of O,O′-dibenzoyl-L-tartaric acid mono(dimethylamide) in 1 mL of CDCl_3_ were recorded and the signals which correspond to aromatic hydrogen and phosphorus atoms were integrated to calculate an enantiomeric excess. The proper selection of the signals was confirmed in the experiment with racemic compound.

#### 3.3.15. Synthesis of (*R*)-6,6′-Bis(diphenylphosphanyl)-*N,N,N’,N’*,2,2′,4,4′-octamethylbiphenyl-3,3′-diamine (BIMAP, 4b) 

In the glass reactor were placed: *(R)*-**BIMAPO** (50 mg), mesitylene (5 mL), and phenylsilane (1 mL). The reactor was sealed and heated to 190 °C for 12 h. After cooling, the solvents were evaporated, and *(R)*-**BIMAP** was isolated by column chromatography (argon flashed column, degassed hexane/ethyl acetate: 160/1). Yield 41 mg (86%), >99% ee. ^31^P NMR (benzene-d_6_): δ = −13.0. ^1^H NMR (benzene-d_6_): δ = 1.73 (6 H, s, CH_3_), 2.24 (6 H, s, CH_3_), 2.66 (12 H, s, CH_3_N), 7.10–7.20 (12 H, m, Ph), 7.38 (2 H, s, CH), 7.62–7.66 (4 H, m, Ph), 7.68–7.72 (4 H, m, Ph). MS (ES): m/z (%) = 666 (40, M + H^+^), 681 (100, M + O + H^+^), 697 (90, M + 2O + H^+^); MS HR (ES): m/z = calcd. 665.3209 (M + H^+^, C_44_H_47_N_2_P_2_), found 665.3186 (M + H^+^, C_44_H_47_N_2_P_2_). Due to its instability in diluted solution of the compound, the specific rotation measurement was not performed.

#### 3.3.16. Determination of Enantiomeric Composition BIMAP (4b)

The solution of 4 mg of BIMAP in 0.4 mL of benzene was added to the solution of *(S)-***15** (3 mg in 1 mL of EtOH). The solution was stirred overnight at ambient temperature. The solvents were evaporated off under the reduced pressure and residual was dissolved in CDCl_3_, and ^31^P NMR spectrum was recorded. The ratio of the signals corresponding to the phosphorus atoms of the diateriomeric complexes **17c**, correspond to the ratio of the enantiomers in the bisphosphine **4b** assessed. ^31^P NMR (CDCl_3_): δ = 14.7 (0.5P, d, *J* = 47.6 Hz, (S, R_a_)), 15.5 (0.5 P, d, *J* = 55.9 Hz, (S, S_a_)), 40.8 (0.5P, d, *J* = 47.6 Hz, (S, R_a_)), 46.3 (0.5 P, d, *J* = 55.9 Hz, (S, S_a_)).

The separation of enantiomers of BIMAP (4b) with utilization of chiral palladium complex (*S*)-15. (S,R_a_)-17b to the slurry of (*S*)-15 (15 mg, 3.3 mmol) in 10 mL of dry degassed methanol the racemic BIMAP (200 mg, 3.0 mmol) was added. After 24 h of stirring at ambient temperature under the argon atmosphere, the insoluble precipitate was filtered off, the solvent was evaporated under the reduced pressure and the residual was crystalized twice from the mixture of ethanol/water (about 40 volume-%) and one additional time from a mixture hexane/ethyl acetate. The obtained in 11% (with respect to the total Pd used) yield yellow crystalline powder of (S,R_a_)-17b has a purity of >98% de. ^31^P NMR (CDCl_3_): δ = 11.1 (1 P, d, J = 47.5 Hz), 37.5 (1 P, d, J = 47.5 Hz). MS (ES): m/z(%) = 968(100), [M-ClO_4_^-^]^+^; 1072(1) {M + H^+^]^+^. The observed isotope profile of [M-ClO_4_^-^] cation was in excellent agreement with the calculated one for the ion [C_58_H_63_N_3_P_2_Pd]^+^. The crystallographic analysis of the obtained complex allowed to assign the absolute configuration of the phosphine. To liberate the (R_a_)-4b from the obtained complex, 7 mg of DPPE in 1 mL of DCM was added to the 20 mg of (S,R_a_)-17b placed under the argon atmosphere into the NMR tube. The progress of the reaction was monitored by ^31^P NMR spectroscopy. After 14 days of the reaction in ambient temperature, the phosphine was chromatographically separated on small SiO_2_-filled column eluted with degassed mixture of hexane/ethyl acetate = 160/1 to yield 8 mg (67%) of enantiomerically pure the (R_a_)-4b. ^31^P NMR (benzene-d_6_): δ = −13.1. ^1^H NMR (benzene-d_6_): δ = 1.72 (6 H, s, CH_3_), 2.24 (6 H, s, CH_3_), 2.65 (12 H, s, CH_3_N), 7.11–7.21 (12 H, m, Ph), 7.38 (2 H, s, CH), 7.62–7.66 (4 H, m, Ph), 7.68–7.72 (4 H, m, Ph). CD (3 × 10^−4^ M/L, Et_2_O): −8.07 (224), −36.66 (243), +9.41(286), +9.4 (313). MS (ES): m/z = 666 [M + H^+^]^+^, 681 [M + O + H^+^]^+^, 697 [M + 2O + H^+^]^+^.

#### 3.3.17. Synthesis of *N,N,N’,N’*-Tetrabutyl-6,6′-bis(diphenylphosphoryl)-2,2′,4,4′-tetramethylbiphenyl-3,3′-diamine (11e)

This compound was prepared from *N,N,N’,N’*-tetrabuthyl-4,4′,6,6′-tetramethyl-5,5′-diamino-2,2′-iodobiophenyl (3 g, 4.2 mmol) according to the procedure of **BIMAPO**. The product was isolated via column chromatography (hexane/acetone: 7/2). Yield 0.32 g (9%). White crystalline powder, unstable in the air. IR (cm^−1^): 3146, 3055, 3009, 2956, 2930, 2871, 2868, 2636, 1574, 1540, 1482, 1458, 1436, 1376, 1282, 1196, 1115, 1102, 748, 721, 696, 557. ^31^P NMR: δ = 29.5. ^1^H NMR: δ = 0.6–0.9 (12 H, m, CH_3_*-CH_2_*), 1.05–1.52 (16 H, m, CH_2_), 1.24 (6 H, s, CH_3_), 2.10 (6 H, s, CH_3_), 2.65–2.75 (4 H, m, CH_2_N), 2.78–2.88 (4 H, m, CH_2_N), 6.79 (2 H, d, *J =* 14.2 Hz, CH), 7.16–7.39 (12 H, m, Ph), 7.55–7.70 (8 H, m, Ph). ^13^C NMR: δ = 14.0 (s, CH_3_), 14.2 (s, CH_3_), 15.8 (s, CH_3_), 20.1 (s, CH_3_), 20.5 (s, CH_2_), 20.6 (s, CH_2_), 31.7 (s, CH_2_), 31.9 (s, CH_2_), 52.7 (s, CH_2_), 53.9 (s, CH_2_), 127.2 (d, *J =* 107.8 Hz, CP), 127.8 (d, *J =* 11.7 Hz, C_o-_H), 130.4 (s, C_p-_H), 130.7 (s, C_p-_H), 132.3 (d, *J =* 8.8 Hz, C_m-_H), 132.5 (d, *J =* 9.8 Hz, C_m-_H), 133.6 (d, *J =* 13.2 Hz, CH), 134.4 (d, *J =* 100.2 Hz, CP), 134.9 (d, *J =* 14.0 Hz, C-*CH_3_*), 135.6 (d, *J =* 103.4 Hz, CP), 136.9 (d, *J =* 11.2 Hz, C-*CH_3_*), 142.9–142.9 (m, CC), 151.8 (d, *J =* 3.4 Hz, CN). MS (ES): m/z (%) = 865 (100, M+H^+^), 887 (40, M + Na^+^); MS HR (ES): m/z = calcd. 865.4985 (M + H^+^, C_56_H_71_N_2_O_2_P_2_), found 865.4953 (M+H^+^, C_56_H_71_N_2_O_2_P_2_). Anal. calcd. for C_56_H_70_N_2_O_2_P_2_ (865.14) C 77.75, H 8.16, N 3.24; found C 78.02, H 8.18, N 3.35.

#### 3.3.18. Synthesis of (5,5′-Dimethoxy-4,4′,6,6′-tetramethylbiphenyl-2,2′-diyl)bis(diphenylphosphane) Dioxide (BIMOPO, 11a)

To a stirred solution of 4,4′,6,6′-tetramethyl-5,5′-dimethoxy-2,2′-dipodobiphenyl **(10a)** (0.66 g, 1.26 mmol) in Et_2_O (20 mL) a 1.3 M solution of n-BuLi in hexane (3.34 mmol) was added dropwise at −20 °C and stirring was continued for 3 h. Then the solution of freshly distilled Ph_2_PCl (1.1 g, 4.66 mmol) in Et_2_O (10 mL) was rapidly added and mixture was stirred for 2 h at −20 °C, next 12 h at room temperature and 6 h at 34 °C. After cooling to room temperature, 10% H_2_O_2_ in 1 M aqueous NaOH (100 mL) and 100 mL CHCl_3_ were added. The solution was stirred vigorously for 1 h, then the organic phase was separated and treated again with a solution of H_2_O_2_ for 3 h. The organic phase was separated, washed with water (50 mL), and dried over MgSO_4_. After evaporation of the solvents, the residue was heated to reflux in hexane (100 mL) for 30 min. After cooling to room temperature, the crude product was filtered off and recrystallized from toluene/chloroform. Yield 0.61 g (72%). White crystalline powder, mp >250 °C. IR (cm^−1^): 3418, 3054, 2926, 2856, 1632, 1589, 1556, 1459, 1436, 1411, 1393, 1281, 1196, 1153, 1116, 1101, 1007, 920, 892, 751, 722, 696, 5573 517, 432. ^31^P NMR: δ = 29.3; ^1^H NMR: δ = 1.30 (6 H, s, CH_3_), 2.20 (6 H, s, CH_3_), 3.59 (6 H, s, OCH_3_), 6.90 (2 H, d, *J =* 13.9 Hz, CH), 729-7.39 (12 H, m, Ph), 7.64-7.73 (8 H, m, Ph); ^13^C NMR: δ = 12.6 (CH_3_), 16.4 (CH_3_), 59.4 (OCH_3_), 127.2 (d, *J =* 106.9 Hz, CP), 128.0 (d, *J =* 11.9 Hz, C_o-_H), 128.8 (d, *J =*. 14.6, C-*CH_3_*), 130.7 (d, *J =* 2.0 Hz, C_p-_H), 130.8 (d, *J =*. 13.6, C-*CH_3_*), 130.9 (d, *J =* 2.4 Hz, C_p-_H), 132.3 (d, *J =* 8.9 Hz, C_m-_H), 132.5 (d, *J =* 10.0 Hz, C_m-_H), 133.6 (d, *J =* 13.5 Hz, CH), 134.0 (d, *J =* 100.4 Hz, CP), 135.1 (d, *J =* 104.8 Hz, CP), 142.8-142.9 (4-peaks, CC), 159.3 (d, *J =* 3.0 Hz, C*OCH_3_*). MS (ES): m/z (%) = 671 (100, M + H^+^), 693 (50, M+Na^+^); MS HR (ES): m/z = Calcd. 671.2475 (M+H^+^, C_42_H_41_O_4_P_2_), found 671.2463 (M + H^+^, C_42_H_41_O_4_P_2_).

#### 3.3.19. Synthesis of (3′,5-Dimethoxy-2′,4,4′,6-tetramethylbiphenyl-2-yl)(diphenyl)phosphane Oxide (12a)

This compound was isolated by column chromatography (hexane/ ethyl acetate/methanol 5/3/0.2) from evaporated residue derived from hexane solution obtained during **BIMOPO** extraction. Yield 0.09 g (15%). White crystalline powder, mp = 147–149 °C (crystallized from benzene/hexane). IR (cm^−1^): 3056, 2953, 2860, 2829, 2731, 2481, 1970, 1895, 1821, 1770, 1589, 1553, 1459, 1438, 1401, 1289, 1262, 1194, 1142, 1115, 1101, 1010, 937, 906, 839, 819, 751, 721, 696, 560, 516. ^31^P NMR: δ = 28.4; ^1^H NMR: δ = 1.55 (3 H, s, CH_3_), 1.74 (3 H, s, CH_3_), 2.10 (3 H, s, CH_3_), 2.20 (3 H, s, CH_3_), 3.47 (3 H, s, CH_3_O), 3.68 (3 H, s, CH_3_O), 6.37 (1 H, d, *J =* 7.7 Hz, CH), 6.56 (1 H, d, *J =* 7.7 Hz, CH), 7.20–7.50 (11 H, m, CH + Ph); ^13^C NMR: δ = 13.0 (s, CH_3_), 13.3 (s, CH_3_), 16.0 (s, CH_3_), 16.2 (s, CH_3_), 59.5 (s, CH_3_O), 59.7 (s, CH_3_), 126.2, 126.4, 127.2, 127.7, 127.8, 129.6, 129.8, 129.9, 130.0, 130.7, 130.8, 130.9, 131.0, 131.1, 131.5, 131.6, 131.7, 131.8, 133.7, 133.8, 133.9, 134.1, 134.4, 134.5, 136.0, 145.0, 156.3, 160.5. MS (ES): m/z (%) = 471 (100, M + H^+^), 493 (50, M+Na^+^). MS HR (ES): m/z = Calcd. 471.2084 (M + H^+^, C_30_H_32_O_3_P), found 471.2099 (M + H^+^, C_30_H_32_O_3_P).

#### 3.3.20. Alternative BIMOPO Synthesis Procedure (11a)

In the sealed reactor were placed: 4,4′,6,6′-tetramethyl-5,5′-dimethoxy-2,2′-diiodobiphenyl **(10a)** (0.16 g, 0,3 mmol), diphenylphosphine oxide (0.2 g, 1 mmol), **DABCO** (0.25 g, 2.2 mmol), **dppb** (6 mg, 0.014 mmol), Pd(OAc)_2_ (3 mg, 0.014 mmol) and CH_3_CN (10 mL). The reactor was sealed and heated to 80–85 °C and the reaction mixture was intensively stirred for 3 days. After cooling, the solvents were evaporated under the reduced pressure and the residue was mixed with CH_2_Cl_2_ (200 mL). The resulting solution was washed with 1 M HCl (2 × 100 mL), treated with 3 portions of 10% H_2_O_2_ in 1 M aqueous NaOH (3 × 100 mL) for 2, 4 and 4 h, respectively, then washed with 1 M aqueous NaOH (100 mL) and dried over MgSO_4_. After solvent evaporation, the mixture composition was determined by HPLC (RP-18 column (250 × 4.5 mm), Mobile phase: MeOH 70%, H_2_O 30%, flow rate: 1.5 mL/min, inj. vol.: 20 μL. **10a** (17 min, 26%); **BIMOPO** (28 min, 3%); **12a** (30 min, 72%), **13a** (55 min, 4%). The resulting mixture was placed again in the reactor, diphenylphosphine oxide (0.2 g, 1 mmol), **DABCO** (0.25 g, 2.2 mmol), triphenylphosphine (8 mg, 0.014 mmol), Pd(OAc)_2_ (3 mg, 0.014 mmol) and CH_3_CN (10 mL) were added. The reaction mixture was heated to 95 °C and intensively stirred for two days, then the temperature was elevated to 125 °C and stirring was continued for two more days. After the reaction mixture was proceeded as described above, **BIMOPO** was isolated by column chromatography (hexane/ethyl acetate/methanol 5/3/0.25); **BIMOPO** yield 86 mg (60%).

*(R)-***BIMOPO** obtained starting from *(R)-***DIDAB** in 35% overall yields after the crystallization from methanol/t-BuOMe has mp= 267 °C and [*α*]_D_^20^ = +78 (*c* = 0.8, CHCl_3_). Those values are in good agreements with the literature ones. [56]

#### 3.3.21. Synthesis of (4,4′,6,6′-Tetramethylbiphenyl-2,2′-diyl)bis(diphenylphosphane) dioxide (*S*)-tetraphempo, (*S*)-11d)

In the sealed reactor were placed: *(S)*-4,4′,6,6′-tetramethyl-2,2′-diiodobiphenyl **(10d)** (0.2 g, 0.4 mmol, 95% ee), diphenylphosphine oxide (0.8 g, 4.3 mmol), **DABCO** (1 g, 8.9 mmol), CuI (0.1 g, 0.5 mmol), TBA·I (0.025 g, 0.068 mmol), triphenylphosphine (0.07 g, 0.27 mmol) Pd(OAc)_2_ (0.02 g, 0.09 mmol) and CH_3_CN (20 mL). Then, the reactor was heated to 90 °C with continuously stirring for 24 h and next 48 h at 125 °C. After cooling, the solvent was evaporated and the residue was dissolved in CH_2_Cl_2_ (200 mL), washed with 1 M HCl (2 × 100 mL) and treated with three portions of 10% H_2_O_2_ in 1 M aqueous NaOH (3 × 100 mL) for 2, 4 and 4 h respectively and finally washed with 1 M aqueous NaOH (100 mL). After drying over the MgSO_4_ and evaporation of the solvent, the crude product was isolated by column chromatography (hexane/ethyl acetate/methanol 5/3/0.25). Yield 135 mg (51%), >90% ee. Pure *(S)*-**TetraPHEMPO** was recrystallized from methylcyclohexane. Yield 100 mg (38%), >99% ee. Colorless crystals, mp = 250 °C. [*α*]_D_^20^ = −34.5 (*c* = 1.0, CH_2_Cl_2_). CD (8.2∙10^−5^M, CH_3_CN): +65.7(187.0), −86.46 (200.0), +27.70 (226.0), +21.29 (237.0), −2.92 (254.0), +0.95 (289.0). IR (cm^−1^): 3144, 3052, 3020, 2987, 2958, 2917, 1589, 1572, 1483, 1436, 1404, 1380, 1309, 1202, 1188, 1150, 1116, 1070, 1028, 995, 868, 752, 721, 695, 555, 534, 484. ^31^P NMR: δ = 30.14. ^1^H NMR: δ = 1.46 (6 H, s, CH_3_), 2.34 (6 H, s, CH_3_), 6.90 (2 H, d, *J =* 14.1 Hz, C_3-_H), 7.00 (2 H, s, C_5-_H), 7.23–7.27 (4 H, m, Ph), 7.32–7.46 (8 H, m, Ph), 7.63–7.72 (8 H, m, Ph). ^13^C NMR: δ = 19.4 (s, CH_3_), 21.3 (s, CH_3_), 127.8 (d, *J =* 12.0 Hz, C_o-_H), 127.9 (d, *J =* 11.5 Hz, C_o-_H), 130.6 (d, *J =* 2.6 Hz, C_p-_H), 130.8 (d, *J =* 2.5 Hz, C_p-_H), 130.9 (d, *J =* 103.8 Hz, C_-_P), 131.3 (d, *J =* 12.9 Hz, C_3-_H), 132.3 (d, *J =* 8.9 Hz, C_m-_H), 132.6 (d, *J =* 10.1 Hz, C_m-_H), 133.7 (d, *J =* 2.8 Hz, C_5-_H), 134.1 (d, *J =* 99.9 Hz, CP), 135.1 (d, *J =* 104.5 Hz, CP), 135.6 (d, *J =* 14.1 Hz, C-CH_3_), 137.3 (d, *J =* 11.0 Hz, C-CH_3_), 140.6 (4 peaks, CC). MS (ES): m/z (%) = 611 (100, M + H^+^), 633 (70, M+Na^+^); MS HR (ES): m/z = calcd. 611.2263 (M+H^+^, C_40_H_37_O_2_P_2_), found 611.2277 (M + H^+^, C_40_H_37_O_2_P_2_).

#### 3.3.22. Synthesis of Diphenyl(2′,4,4′,6-tetramethylbiphenyl-2-yl)phosphane Oxide, (12d)

This compound was isolated by column chromatography (hexane/ethyl acetate/methanol 5/3/0.2) as a byproduct in **TetraPHEMPO** synthesis. Yield 0.083 g (52%). White powder, mp = 256 °C ^31^P NMR (161.94 MHz): δ = 28.0. ^1^H NMR(400.04 MHz): δ = 1.61 (3 H, s, CH_3_), 1.85 (3 H, s, CH_3_), 2.19 (3 H, s, CH_3_), 2.32 (3 H, s, CH_3_), 6.50–6.62 (3 H, m, CH), 7.20–7.70 (12 H, m, Ph). MS (ES): m/z (%) = 411 (100, M + H^+^), 433 (50, M + Na^+^).

#### 3.3.23. Synthesis of (*S*)-(4,4′,6,6′-Tetramethylbiphenyl-2,2′-diyl)bis(diphenylphosphane) ((*S*)-tetraPHEMP, (*S*)-4d)

Into a glass reactor *(S)*-**TetraPHEMPO** (30 mg) and toluene (10 mL) were placed and during vigorous stirring, tributylamine (1 mL) and trichlorosilane (0.16 mL) were sequentially added. The reactor was sealed and heated to 120 °C for 24 h. Upon cooling, the reaction mixture was poured into 30% aqueous NaOH (30 mL) and stirred vigorously for 2 h. The organic phase was separated, washed with water (10 mL), dried over MgSO_4,_ the solvent was evaporated and the product was isolated by column chromatography (argon flashed column, degassed hexane/ethyl acetate: 160/1). Yield 21 mg (75%). White powder, mp = 216–218 °C (crystallized from ethyl acetate), lit. 217.5–219 °C. ^31^P NMR (benzene-d_6_): δ = −12.1. The obtained spectra were in accordance with literature data [18].

#### 3.3.24. Synthesis of (5,5′-Dichloro-4,4′,6,6′-tetramethylbiphenyl-2,2′-diyl)bis(diphenylphosphane) Dioxide (BIClPO, 11c)

To a stirred at −50 °C solution of 4,4′,6,6′-tetramethyl-5,5′-dichloro-2,2′-dipodobiphenyl **(10c)** (1 g, 1.88 mmol) in Et_2_O (50 mL) a 1.3 M solution of n-BuLi in hexane (4.7 mL) was added dropwise and stirring was continued for 3 h at −20 °C and 20 min at 0 °C. Then the reaction mixture was chilled down to −50 °C and a solution of freshly distilled Ph_2_PCl (2 g, 9.1 mmol) in Et_2_O (40 mL) was rapidly added and mixture was stirred for 2 h at −20 °C, next 12 h at ambient temperature and 6 h at 34 °C. After cooling to room temperature, 10% H_2_O_2_ in 1 M aqueous NaOH (100 mL) and 100 mL CHCl_3_ were added. The solution was stirred vigorously for 6 h, the organic phase was separated and treated again with a solution of H_2_O_2_ for 18 h. The organic phase was separated and washed with water (50 mL) and dried over MgSO_4_. After evaporation of the solvents, the product was isolated by chromatography (hexane/ethyl acetate/methanol = 5/3/2.5). Yield 0.38 g (30%). White crystalline powder, mp > 250 °C. IR (cm^−1^): 3415, 3054, 2952, 2920, 2853, 1739, 1633, 1589, 1542, 1483, 1436, 1381, 1244, 1205, 1188, 1116, 1048, 1029, 1012, 997, 895, 877, 750, 722, 695, 551, 501. ^31^P NMR: δ = 29.3; ^1^H NMR: δ = 1.34 (6 H, s, CH_3_), 2.30 (6 H, s, CH_3_), 6.96 (2 H, d, J = 13.7 Hz, CH), 7.30–7.35 (4 H, m, Ph), 7.38–7.44 (6 H, m, Ph), 7.47–7.51 (2 H, m, Ph), 7.57–7.61 (4 H, m, Ph), 7.73–7.78 (4 H, m, Ph); ^13^C NMR: δ = 17.2 (s, CH_3_), 21.3 (s, CH_3_), 128.1 (d, *J =* 12.3 Hz, C_o-_H), 128.2 (d, *J =* 11.6 Hz, C_o-_H), 130.6 (d, J = 104.6 Hz, CP), 131.1 (d, J = 2.5 Hz, C_p-_H), 131.2 (d, J = 2.5 Hz, C_p-_H), 132.3 (d, J = 9.1 Hz, C_m-_H), 132.5 (d, J = 13.5 Hz, CH), 132.6 (d, J = 10.0 Hz, C_m-_H), 132.9 (d, J = 100.0 Hz, CP), 134.4 (d, J = 104.6 Hz, CP), 134.9 (d, J = 13.6 Hz, C-CH_3_), 135.8 (d, J = 10.9 Hz, C-CH_3_), 138.6 (d, J = 3.1 Hz, CCl), 141.5–141.6 (4 peaks, CC). MS (ES): m/z (%) = 679 (100, M+H^+^). MS HR (ES): m/z = calcd. 679.1484 (M + H^+^, C_40_H_35_O_2_Cl_2_P_2_), found. 679.1481 (M + H^+^, C_40_H_35_O_2_Cl_2_P_2_).

#### 3.3.25. Synthesis of (3′,5-Dichloro-6′-iodo-2′,4,4′,6-tetramethylbiphenyl-2-yl)(diphenyl)phosphane Oxide (13c)

This compound was isolated by column chromatography (hexane/ethyl acetate/methanol 5/3/0.2) as a byproduct in **BIClPO** synthesis. Yield 0.25 g (22%). White crystalline powder, mp = 178–180 °C. IR (cm^−1^): 3049, 2918, 2852, 1821, 1739, 1574, 1534, 1480, 1437, 1379, 1272, 1241, 1194, 1179, 1112, 1100, 1049, 1012, 997, 885, 757, 719, 695, 555, 499, 426. ^31^P NMR: δ = 28.0. ^1^H NMR: δ = 1.85 (3 H, s, CH_3_), 1.97 (3 H, s, CH_3_), 2.24 (3 H, s, CH_3_), 2.39 (3 H, s, CH_3_), 7.26–7.32 (3 H, m, CH, Ph), 7.33 (1 H, s, CH), 7.37–7.42 (3 H, m, Ph), 7.46–7.50 (1 H, m, Ph), 7.54–7.66 (4 H, m, Ph). ^13^C NMR: δ = 17.4 (CH_3_), 20.0 (CH_3_), 20.4 (CH_3_), 21.2 (CH_3_), 99.7 (CI), 127.8, 127.9, 128.1, 128.2, 128.6, 129.0, 131.0, 131.1, 131.4, 131.5, 131.8, 131.9, 132.0, 132.1, 132.2, 132.3, 132.4, 132.5, 132.6, 132.9, 135.1, 135.5, 135.6, 136.0, 137.4, 137.7, 139.8, 141.1, 146.2. MS (ES): m/z (%) = 627 (100, M+Na^+^), 629 (35). MS HR (ES): m/z = calcd. 626.9879 (M+Na^+^, C_28_H_24_ONaCl_2_IP), found. 626.9878 (M+Na^+^, C_28_H_24_ONaCl_2_IP). Anal. calcd. for C_28_H_24_OCl_2_IP (605.29) C 55.56, H 4.00, I 20.97; found. C 55.65, H 4.00, I 20.89.

#### 3.3.26. Synthesis of (3′,5-Dichloro-2′,4,4′,6-tetramethylbiphenyl-2-yl)(diphenyl)phosphane Oxide (12c)

This compound was isolated by column chromatography (hexane/ethyl acetate/methanol: 5/3/0.2) as a byproduct in **BIClPO** synthesis. Yield 0.05 g (5%). White crystalline powder, mp = 188–190 °C. IR (cm^−1^): 3054, 3021, 2955, 2919, 2856, 2734, 1957, 1891, 1830, 1768, 1681, 1689, 1535, 1483, 1435, 1378, 1308, 1273, 1203, 1194, 1116, 1101, 1049, 997, 885, 814, 787, 749, 724, 693, 557, 498. ^1^H NMR: δ = 1.56 (3 H, s, CH_3_), 1.93 (3 H, s, CH_3_), 2.25 (3 H, s CH_3_), 2.39 (3 H, s, CH_3_), 6.69 (1 H, d, *J =* 7.9 Hz, CH), 6.80 (1 H, d, *J =* 7.9 Hz, CH), 7.25–7.85 (11 H, m, CH + Ph). MS (ES): m/z (%) = 479 (100, M + H^+^), 501 (55, M + Na^+^). MS HR (ES): m/z = calcd. 479.1093 (M + H^+^, C_28_H_26_OCl_2_P), found. 479.1083 (M + H^+^, C_28_H_26_OCl_2_P).

#### 3.3.27. Synthesis of (*S*)-(5,5′-Dichloro-4,4′,6,6′-tetramethylbiphenyl-2,2′-diyl)bis(diphenylphosphane) Dioxide ((*S*)-BIClPO, (*S*)-11c) 

To a filtered solution of *(-)*-DBTA∙H_2_O (0.29 g, 0.77 mmol) and **BIClPO** (0.4 g, 0.59 mmol) in CH_2_Cl_2_ (10 mL) CCl_4_ (50 mL) was added. Then, 10 mL of solvent was evaporated at atmospheric pressure and the residue was slowly cooled down without stirring to room temperature. After a few days the colorless needles of the **BIClPO**∙DBTA complex were filtered off. Mp = 233 °C. To isolate the bisphosphone dioxide, the complex was dissolved in a minimum amount of CH_2_Cl_2_ and *(S)*-**BIClPO** was subjected to column chromatography (Al_2_O_3_ as a stationary phase, eluent: hexane/ethyl acetate/methanol (5:3:0.2). Yield 0.08 g (40%), >99% ee. Mp > 250 °C, [α]_D_^20^ = −119 (*c* = 0.87, CH_2_Cl_2_). CD (10^−4^ M, CH_3_CN): −86.73 (208.8), +15.86 (228.6), +9.98 (245.6), −1.10 (255.0), +1.29 (288.6). Next, a 15 mL of CCl_4_ was added to the warm mother liquor and after several days obtained complex was filtered off giving *(S)*-**BIClPO** (0.08 g, >21% ee). The crystallization procedure was repeated again and *(R)*-**BAClPO** 0.13 g (65%), >99% ee was isolated. [α]_D_^20^ = +118 (*c* = 0.85, CH_2_Cl_2_).

#### 3.3.28. Determination of Enantiomeric Composition BClPO (11c)

The enantiomeric purity of obtained bisphosphine dioxide was determined by NMR technique: [52,54] the ^1^H and ^31^P spectra of solution of 2 mg of compounds and 3 mg of mandelic acid in 1 mL of CDCL_3_ were recorded and the signals which correspond to aromatic hydrogen and phosphorus atoms were integrated to calculate an enantiomeric excess. The proper selection of the signals was confirmed in the experiment with racemic compound.

#### 3.3.29. Synthesis of (*S*)-(5,5′-Dichloro-4,4′,6,6′-tetramethylbiphenyl-2,2′-diyl)bis(diphenylphosphane) ((*S*)-BIClP, (*S*)-4c)

Into a glass reactor *(S)*-**BIClPO** (60 mg) and toluene (20 mL) were placed and during vigorous stirring, tributylamine (2 mL) and trichlorosilane (0.36 mL) were sequentially added. The reactor was sealed and heated to 120 °C for 24 h. Upon cooling, the reaction mixture was poured into 30% aqueous NaOH (30 mL) and stirred vigorously for 2 h. The organic phase was separated, washed with water (10 mL), and dried over MgSO_4_. The solvent was evaporated and the product was isolated by column chromatography (argon flashed column, degassed hexane/ethyl acetate: 160/1). Yield 47 mg (79%). ^31^P NMR (benzene-d_6_): δ = −12.0; ^1^H NMR (benzene-d_6_): δ = 1.59 (6 H, s, CH_3_), 2.19 (6 H, s, CH_3_), 7.10–7.20 (12 H, m, Ph), 7.32 (2 H, s, CH), 7.44–7.50 (4 H, m, Ph), 7.63–7.69 (4 H, m, Ph). MS (ES): m/z (%) = 647 (20, M + H^+^), 663 (30, M + O + H^+^), 679 (100, M + 2O + H^+^). Due to low stability of the compound in diluted solution the specific rotation measurement was not performed.

#### 3.3.30. Determination of Enantiomeric Composition BIClP (4c) 

The solution of 2.6 mg of **BIClP** in 0.3 mL of benzene was added to the solution of *(S)-***15** (2 mg in 1 mL of EtOH). The solution was stirred overnight at ambient temperature. The solvents were evaporated under the reduced pressure and residual was dissolved in CDCl_3_, and ^31^P NMR spectrum was recorded. The ratio of the signals corresponding to the phosphorus atoms of the diateriomeric complexes **17c**, correspond to the ratio of the enantiomers in the bisphosphine **4c** assessed. ^31^P NMR (CDCl_3_): δ = 12.8 (1P, d, *J* = 53.7 Hz, (S, S_a_)), 42.9 (1P, d, *J* = 53.7 Hz, (S, S_a_)).

#### 3.3.31. Synthesis of 6,6′-Bis(diphenylphosphoryl)-2,2′,4,4′-tetramethylbiphenyl-3,3′-diol (11f) 

The slurry of **BIMOPO** (0.5 g, 0.75 mmol) in HBr (40% in CH_3_CO_2_H, 15 mL) had been stirred at ambient temperature for 14 days. Next, the solvents were evaporated under the reduced pressure and residual was dissolved in 15 mL of dry ethanol which had contained 1.5 g NaOH. After the 3 h of stirring under the argon atmosphere at ambient temperature, the solution was cooled down to 0 °C and acidified with 1 M H_2_SO_4_ to obtain pH = 2. Formed white precipitate was filtered off, washed with 50 mL of water and 10 mL of methanol and dried under the reduced pressure to furnish 0.48 g of pure product with mp > 250 °C. IR (cm^−1^): 3250, 3145, 3054, 2920, 1962, 1896, 1822, 1634, 1591, 1556, 1436, 1294, 1156, 1116, 1099, 1027, 997, 903, 747, 722, 694, 558, 509, 444. ^31^P NMR (in DMSO-d_6_): δ = 28.5. ^1^H NMR (in DMSO-d_6_): δ = 1.14 (6 H, s, CH_3_), 2.05 (6 H, s, CH_3_), 6.65 (2 H, d, J = 13.9 Hz, CH), 7.34 (4 H, td, J = 7.8, 2.5 Hz, Ph), 7.47–7.65 (16 H, m, Ph), 8.51 (2 H, s, OH). MS (ES): m/z (%) = 643 (80, M + H^+^), 665 (100, M + Na^+^); MS HR (ES): m/z = calc. 665.1981 (M + Na^+^, C_40_H_36_O_4_P_2_Na), found. 665.1988 (M + Na^+^, C_40_H_36_O_4_P_2_Na). Anal. calc. for C_40_H_36_O_4_P_2_ (642.68) C 74.76, H 5.65; for (C_40_H_36_O_4_P_2_ + ½ H_2_O) C 73.73, H 5.68; found. C 74.08, H 5.70.

#### 3.3.32. Synthesis of [5,5′-Bis(benzyloxy)-4,4′,6,6′-tetramethylbiphenyl-2,2′-diyl]bis(diphenylphosphane) Dioxide (11g) 

0.1 g, (0.16 mmol) of **11f** was added to stirred mixture of 10 g K_2_CO_3_ in 50 mL of dry DMF. After a 5 min 10 mg (0.03 mmol) of TBA·Br was added, followed by addition of 1 mL (8 mmol) of benzyl bromide. The reaction mixture was argonated and stirred at 45 °C for 24 h, at 50 °C for 96 h and at 70 °C for 10 days. Unreacted K_2_CO_3_ was filtered off and solvent evaporated under the reduced pressure. The residual was dissolved in 50 mL of CHCl_3_, washed with 1 M aqueous NaOH (2 × 30 mL), 1 M aqueous HCl (2 × 30 mL) and dried with MgSO_4_. The product was purified on SiO_2_ column chromatography using as eluent mixture hexane/ethyl acetate/methanol = 5/3/0.25) in 99% yield (0.1 g). White crystalline solid with mp > 250 °C (from toluene). IR (cm^−1^): 3420, 3054, 2922, 2867, 1607, 1589, 1556, 1497, 1454, 1436, 1376, 1280, 1192, 1160, 1115, 1101, 986, 898, 751, 721, 696, 556, 509. ^31^P NMR: δ = 29.53; ^1^H NMR: δ = 1.35 (6 H, s, CH_3_), 2.21 (6 H, s, CH_3_), 4.60 (2 H, d, J = 11.3 Hz, HCH), 4.74 (2 H, d, J = 11.3 Hz, HCH), 6.93 (2 H, d, J = 13.9 Hz, CH), 7.26–7.48 (12 H, m, Ph), 7.66–7.76 (8 H, m, Ph). ^13^C NMR: δ = 13.0 (s, CH_3_), 16.7 (CH_3_), 73.5 (s, CH_2_), 127.2 (d, J = 106.6 Hz, C-P), 127.6 (s, CH), 127.9 (d, J= 14.0 Hz, Co-H), 128.0 (d, J= 12.4 Hz, Co-H), 128.1 (s, CH), 128.5 (s, CH), 129.2 (d, J = 14.6 Hz, C-CH_3_), 130.8 (d, J = 2.2 H, Cp-H), 130.9 (d, J = 11.7 Hz, C-CH_3_), 131.0 (d, J = 2.4 Hz, Cp-H), 132.3 (d, J = 8.9 Hz, Cm-H), 132.5 (d, J = 10.1 Hz, Cm-H), 133.7 (d, J = 13.7 Hz, C-H), 134.2 (d, J = 100.6 Hz, CP), 135.2 (d, J = 104.9 Hz, CP), 137.5 (s, C-CH_2_), 142.9-143.0 (4 peaks, CC), 158.1 (d, J = 3.0 Hz, C-OCH_2_). MS (ES): m/z (%) = 823 (100, M + H^+^), 845 (10, M+Na^+^); MS HR (ES): m/z = calc. 823.3101 (M + H^+^, C_54_H_49_O_4_P_2_), found. 823.3129 (M + H^+^, C_54_H_49_O_4_P_2_). Anal. calc. for C_54_H_48_O_4_P_2_ (822.93) C 78.82, H 5.88; found. C 78.76, H 5.96.

### 3.4. Cytotoxicity Assay

For cytotoxicity assay cells were seeded in 96-well microplates at a density of 2.5 × 10^4^ cells/mL in 100 μL DMEM + GlutaMAX supplemented with 10% heat-inactivated FBS in three sets for different periods of tested compound exposure. After 24 h of cell attachment, plates were washed with 100 μL/well of Dulbecco’s phosphate buffered saline (DPBS) and treated with specific concentration of each compound prepared in fresh FBS-free medium for 24, 48 and 72 h. Due to differences in solubility, the compounds were divided into two groups in order to obtain the maximum possible concentration while maintaining complete solubility and obtaining a homogeneous solution for a given compound. The group of twelve compounds that showed relatively low solubility were tested at a final concentration of 50 µM, while the second group of eight compounds at a final concentration of 200 µM. All compounds were dissolved in DMSO, in order to prepare a stock solutions. During experiments, stock solution was diluted in cell culture medium to reach a maximum 0.01% *w/v* DMSO in final solution. Each concentration was tested in triplicate. All sets included wells containing 0,01% DMSO as a negative control and 1% of Triton X-100 as a positive control. The cytotoxicity of compounds was assessed using MTT assay as described below. Following 24, 48 and 72 h of compound exposure, control medium or test exposures medium were removed, the cells were rinsed with DPBS and 100 μL of fresh medium (without FBS or antibiotics) containing 0.5 mg/mL of MTT was added to each well and the plates were incubated for 3 h at 37 °C in a 5% CO_2_ humidified incubator. After incubation period the medium was discarded, the cells were washed with 100 μL of DPBS and 100 μL of DMSO was added to each well to extract the dye. The plate was shaken for 10 min and the absorbance was measured at 570 nm. Viability was calculated as the ratio of the mean of OD obtained for each condition to the control condition.

## 4. Conclusions

In summary, we have designed and synthesized atropisomer 4,4′,6,6′-tetramethyl biaryls bearing phosphorus functionalities at 2,2′-positions and different substituents at 5,5′-positions from one universal non-racemic 5,5′-diamine substituted precursor **DIDAB**. The other approaches leading to the *C2*-symmetric enantiomerically pure bisphosphine ligands were practically assessed. We demonstrated that optical resolution of racemic mixtures could be carried out in different stages of ligand synthesis and in various ways such as: fractional crystallization of phosphine oxide complex with the chiral acid, with the use of chiral palladium complexes, with application of the chiral high performance liquid chromatography for individual ligands or their precursors, otherwise a single early stage precursor could be prepared in an enantiomerically pure form and used to give access to the entire series of chiral non-racemic ligands. The new atropisomeric ligands with enantiomeric purity over 99% ee were obtained in reasonable yields. The compounds at tested concentrations of 50 and 200 µM showed various biological activity against normal human dermal fibroblast, ranging from inactivity for non-phosphorus contained compounds through time-dependent action for some mono-phosphine oxides and ending up with high toxicity for bis-phosphine dioxides.

## Data Availability

CCDC 2141371-2141373 contain the supplementary crystallographic data. These data can be obtained free of charge via http://www.ccdc.cam.ac.uk/conts/retrieving.html (accessed on 2 July 2022), or from the Cambridge Crystallographic Data Centre, 12 Union Road, Cambridge CB2 1EZ, UK; Fax: (+44)-1223–336-033; or e-mail: deposit@ccdc.cam.ac.uk.

## Supplementary Materials

The following supporting information can be downloaded at: https://www.mdpi.com/article/10.3390/molecules27175504/s1, Table S1: Crystal data and structure refinement for DIDAB, BICLPO and BIMAPO; Table S2: Selected bond lengths in DIDAB, BICLPO and BIMAPO (Å); Table S3: Torsion angles in DIDAB, BICLPO and BIMAPO (°); Table S4: Hydrogen bonding parameters (Å,°); IR, NMR and MS Spectra.

## Sample Availability

Samples of the compounds are available from the authors.
