# Supplementary material for: A Modular Approach to Atropisomeric Bisphosphines of Diversified Electronic Density on Phosphorus Atoms
Source: Molecules. 2022 Aug 27;27(17):5504. doi: 10.3390/molecules27175504 (PMC9457989; doi:10.3390/molecules27175504)
Supplement: Supplementary file 1 [file molecules-27-05504-s001.zip › molecules-1855524-supplementary.pdf]

molecules

MDPI

A modular approach to atropisomeric bisphosphines of diversified electronic density on phosphorus atoms

Oleg M. Demchuk,[1]* A. Martyna,[1] M. Kwaśnik,[1] K. Szwaczko,[2] D. Strzelecka,[2] B. Miroslaw,[2] K. Michał Pietrusiewicz,[2] Z. Lipkowska[3]

[1] Institute of Biological Sciences, Faculty of Science and Health, The John Paul II Catholic University of Lublin, Konstantynów 1J/4.03, 20-708 Lublin, Poland.

[2] Faculty of Chemistry, Maria Curie-Sklodowska University, Gliniana 33, 20-031 Lublin, Poland

[3] Institute of Organic Chemistry PAS, Kasprzaka 44/52, 01-224 Warsaw, Poland

* Correspondence: Oleh.Demchuk@KUL.Lublin.pl

Electronic Supporting Information

Crystallographic data

The X-ray data were collected at Nonius Kappa-CCD diffractometer using the MoKα = 0.71073 Å wavelength at 150 K for **DIDAB** and at room temperature for all other compounds. The structures were solved by direct methods (SHELXS) and refined by the full-matrix least-squares method based on F^2 [60]. Hydrogen atoms were placed at calculated positions. The water molecule in **BIClPO** occupies a special position at 2-fold axis. Benzene molecule in **BIMAPO** was refined isotropically because of positional disorder.

***(R)*-DIDAB**	***(S)*-BIClPO**	***(S)*-BIMAPO**
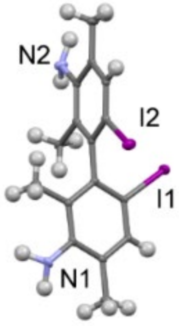	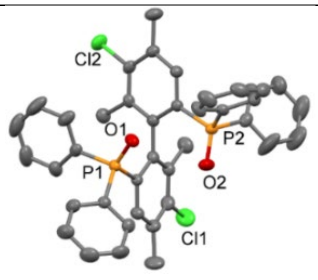	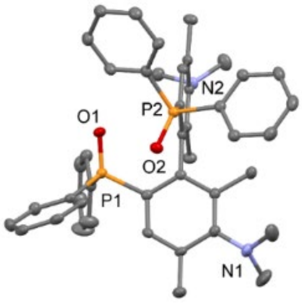
CCDC No.: 2141373	CCDC No.: 2141372	CCDC No.: 2141371

molecules MDPI

Table S1 Crystal data and structure refinement for DIDAB, BICLPO and BIMAPO.

Identification code	DIDAB	BICLPO	BIMAPO
	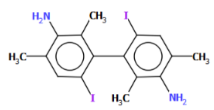	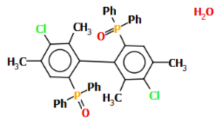	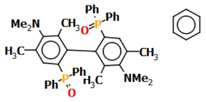
Empirical formula	$C_{16}H_{18}I_2N_2$	$C_{80}H_{70}Cl_4O_5P_4$	$C_{50}H_{52}N_2O_2P_2$
Formula weight	492.12	1377.04	774.88
Temperature/K	150(2)	293(2)	293(2)
Crystal system	Tetragonal	Trigonal	Orthorhombic
Space group	$P4_3$	$P3_221$	$P2_12_12_1$
a/Å	9.42900(10)	13.13700(10)	13.0850(2)
b/Å	9.42900(10)	13.13700(10)	18.1300(3)
c/Å	19.2620(2)	34.9290(3)	18.7050(3)
α/°	90.00	90.00	90.00
β/°	90.00	90.00	90.00
γ/°	90.00	120.00	90.00
Volume/$Å^3$	1712.51(3)	5220.46(9)	4437.41(12)
Z	4	3	4
$ρ_{calc}g/cm^3$	1.909	1.314	1.160
$μ/mm^{-1}$	3.666	0.315	0.138
F(000)	936.0	2154.0	1648.0
Crystal size/mm^3	0.30 × 0.25 × 0.22	0.30 × 0.28 × 0.24	0.32 × 0.28 × 0.25
Radiation	MoKα (λ = 0.71073)	MoKα (λ = 0.71073)	MoKα (λ = 0.71073)
2Θ range for data collection/°	6.04 to 54.98	4.28 to 54.94	4.9 to 54.96
Index ranges	-12 ≤ h ≤ 12, -12 ≤ k ≤ 12, -25 ≤ l ≤ 25	-17 ≤ h ≤ 17, -17 ≤ k ≤ 17, -45 ≤ l ≤ 45	-16 ≤ h ≤ 16, -23 ≤ k ≤ 23, -24 ≤ l ≤ 24
Reflections collected	3920	15710	10165
Independent reflections	3920 [R_{int} = 0.0105, R_{sigma} = 0.0197]	7960 [R_{int} = 0.0279, R_{sigma} = 0.0396]	10165 [R_{int} = 0.0176, R_{sigma} = 0.0267]
Data/restraints/parameters	3920/1/185	7960/0/424	10165/0/471
Goodness-of-fit on F^2	1.089	1.118	1.099
Final R indexes [I>=2σ (I)]	R_1 = 0.0193, wR_2 = 0.0461	R_1 = 0.0577, wR_2 = 0.1161	R_1 = 0.0493, wR_2 = 0.1365
Final R indexes [all data]	R_1 = 0.0197, wR_2 = 0.0463	R_1 = 0.0773, wR_2 = 0.1218	R_1 = 0.0514, wR_2 = 0.1381
Largest diff. peak/hole / e $Å^{-3}$	0.44/-0.54	0.25/-0.32	0.62/-0.44
Flack parameter	0.036(19)	0.19(7)	0.05(11)

molecules MDPI

Table S2. Selected bond lengths in DIDAB, BICLPO and BIMAPO (Å).

DIDAB		BICLPO		BIMAPO	
Bond	Length	Bond	Length	Bond	Length
I2-C12	2.104(3)	P1-O1	1.4830(19)	P1-O1	1.477(3)
I1-C1	2.100(3)	P1-C1	1.808(3)	P1-C1	1.805(4)
N1-C4	1.411(4)	P1-C7	1.823(3)	P1-C7	1.813(4)
N2-C9	1.396(4)	P1-C13	1.824(3)	P1-C13	1.821(4)
		P2-O2	1.477(2)	P2-O2	1.480(3)
		P2-C29	1.806(3)	P2-C40	1.815(4)
		P2-C35	1.810(3)	P2-C34	1.821(4)
		P2-C28	1.826(3)	P2-C33	1.830(4)
		Cl2-C24	1.751(3)	N2-C29	1.440(5)
		Cl1-C17	1.748(3)	N2-C27	1.444(5)
				N2-C28	1.448(5)
				N1-C18	1.409(5)
				N1-C19	1.450(7)
				N1-C20	1.457(6)

Table S3. Torsion angles in DIDAB, BICLPO and BIMAPO [°].

DIDAB		BICLPO		BIMAPO	
Torsion	Value	Torsion	Value	Torsion	Value
C1-C6-C7-C12	-95.4(3)	C13-C20-C21-C28	87.8(3)	C33-C24-C23-C21	-91.6(4)
C12-C7-C6-C5	82.2(4)	C28-C21-C20-C18	-95.0(3)	C13-C23-C24-C33	93.2(5)
C1-C6-C7-C8	82.3(4)	C13-C20-C21-C22	-94.2(3)	C21-C23-C24-C25	86.5(4)
C5-C6-C7-C8	-100.2(3)	C18-C20-C21-C22	83.0(3)	C13-C23-C24-C25	-88.6(5)

Table S4. Hydrogen bonding parameters [Å,°].

Crystal	Donor	Acceptor	D-H (Å)	H...A (Å)	D...A (Å)	D-H...A (°)	Symmetry operation
BICLPO	O3-H3A	O2	0.98	1.869(4)	2.829(3)	167.1(2)	–
	C8-H8	O3	0.93	2.395(4)	3.298(3)	163.9(2)	–
DIDAB	N2-H2A	N1	1.01	2.389(3)	3.229(4)	140.0(2)	-1+x, y, z
BIMAPO	C41-H41	O1	0.93	2.588(5)	3.324(5)	136.4(2)	½+x, ½-y, -z
	C4-H4	O2	0.93	2.493(4)	3.388(5)	162.1(2)	½-x, -y, -½+z

molecules

IR, NMR and MS Spectra

Nuclear magnetic resonance (NMR) spectra were recorded on a Bruker AV300 (^{1}H 300 MHz, ^{31}P 121.5 MHz, ^{13}C NMR 75 MHz) and Bruker AV500 (^{1}H 500 MHz, ^{31}P 202 MHz, ^{13}C NMR 126 MHz) spectrometers (Bruker; Billerica, Ma., USA). All spectra were recorded in $CDCl_3$ solutions, unless mentioned otherwise, and the chemical shifts (δ) are expressed in ppm using internal reference to TMS and external reference to 85% H_3PO_4 in D_2O for ^{31}P. Coupling constants (J) are given in Hz. The abbreviations of signal patterns are as follows: *s*-singlet, *d*-doublet, *t*-triplet, *q*-quartet, *m*-multiplet, *b*-broad, and *i*-intensive. The IR spectra were recorded in KBr pallets and with ATR module on the Nicolet 8700A FTIR-ATR spectrometer: wave numbers are in cm^{-1}.

1-iodo-2,4-dimethyl-3-nitrobenzene (7)

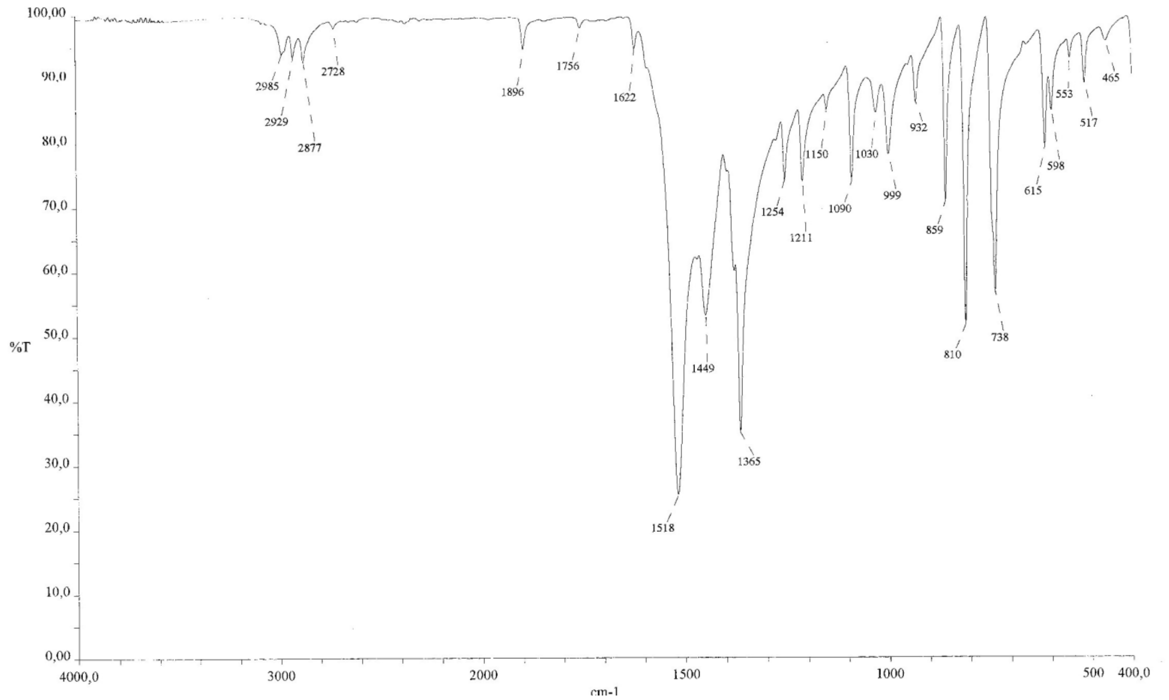

molecules MDPI

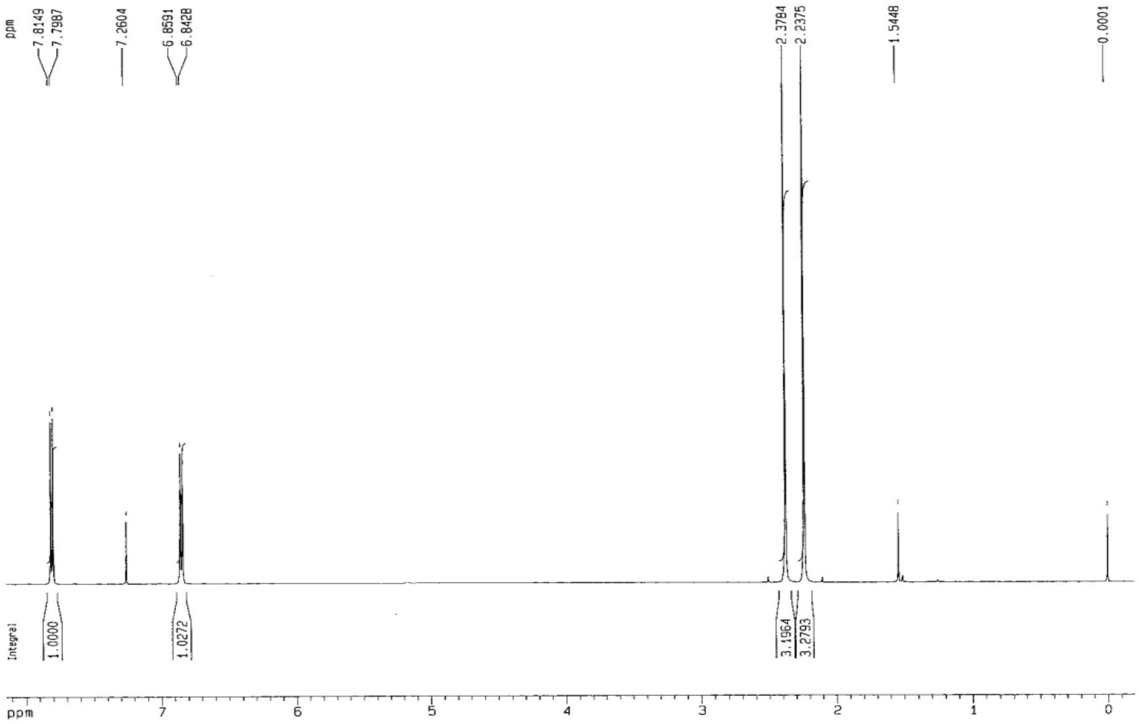

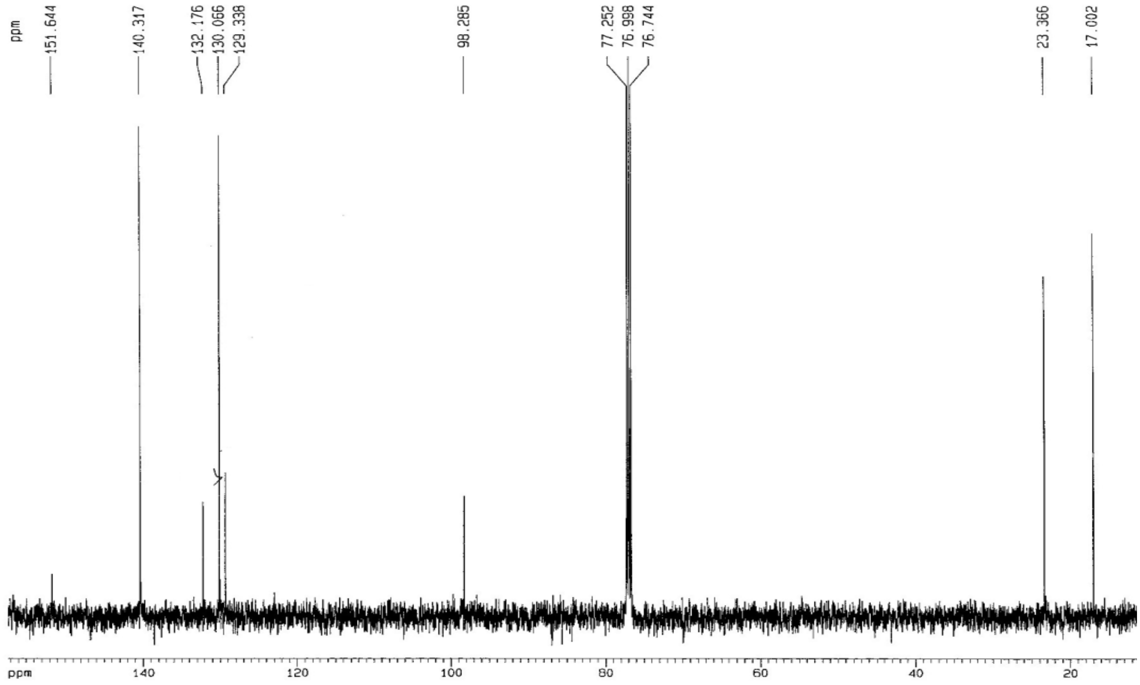

2,2', 4,4'-tetramethyl-3,3'-dinitrobiphenyl (8)

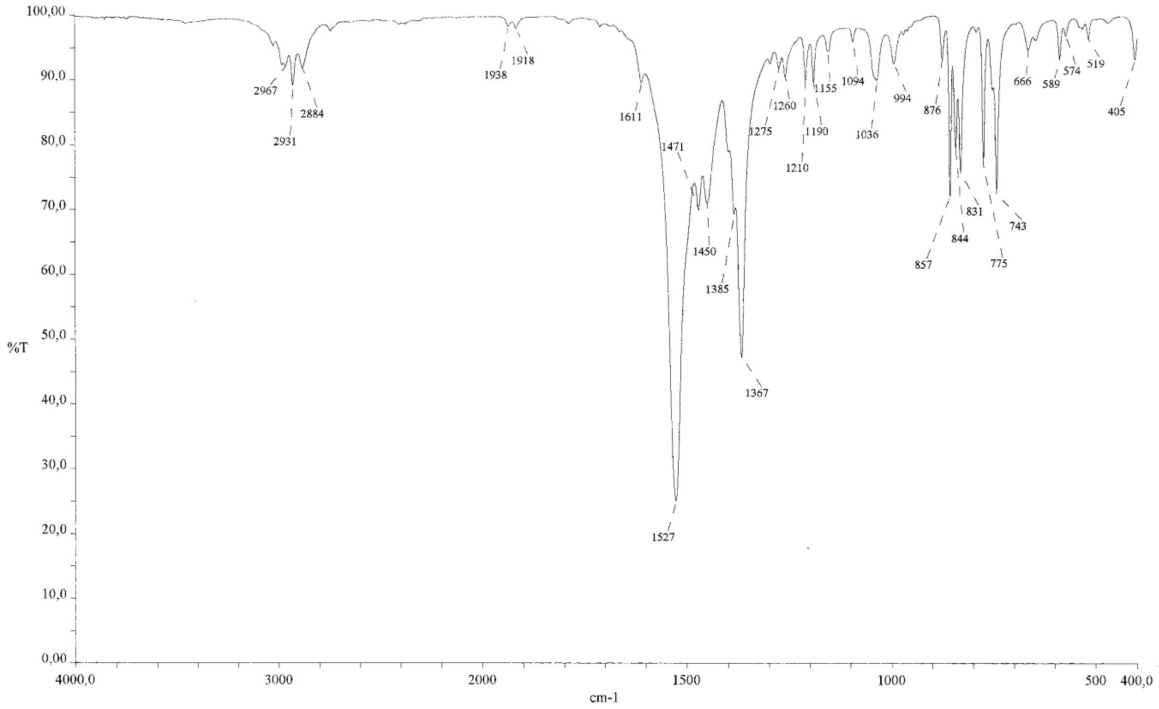

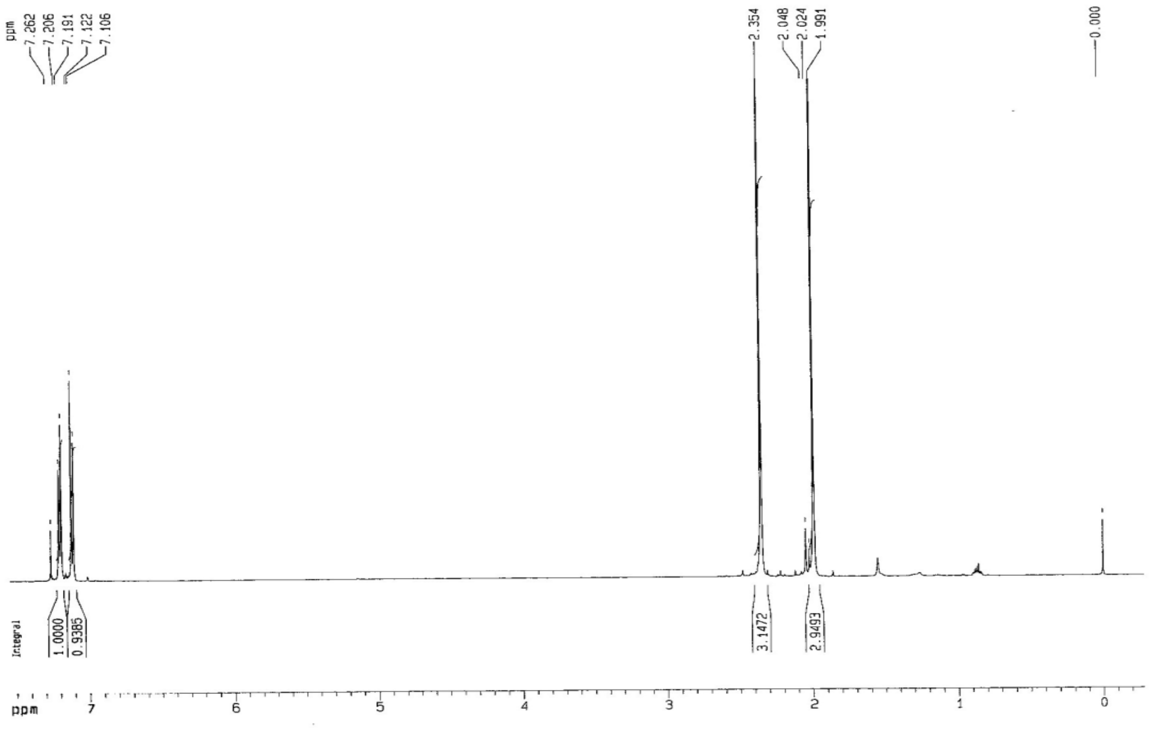

molecules
MDPI

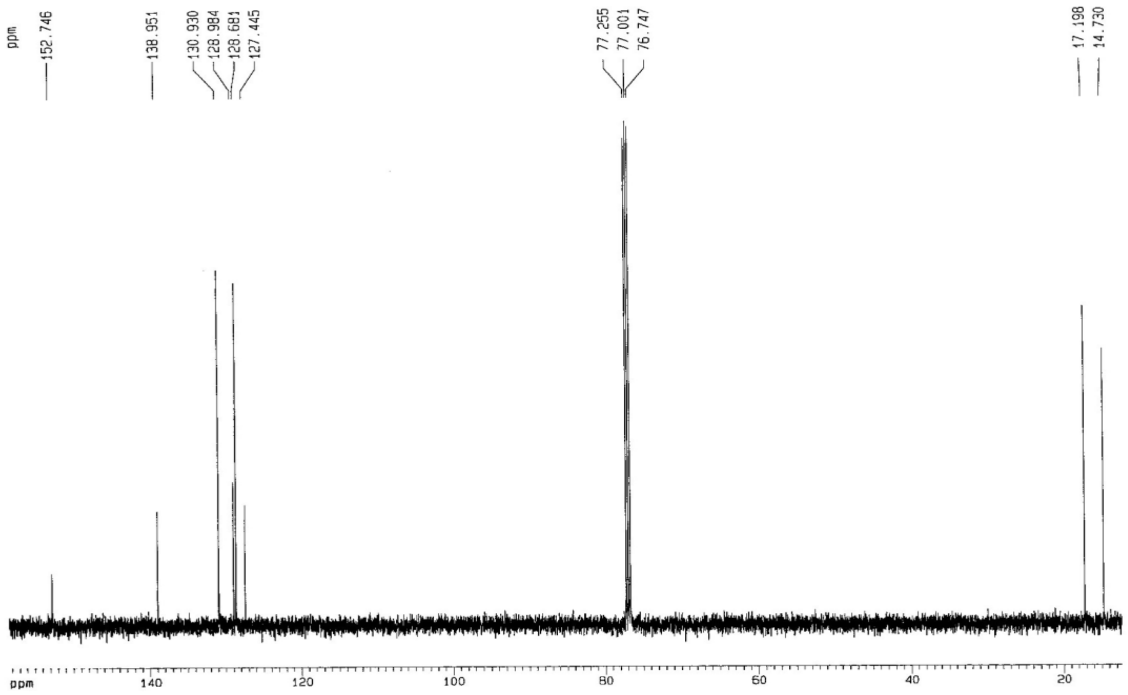

molecules

MDPI

2,2',4,4'-tetramethyl-3,3'-diaminobiphenyl (9)

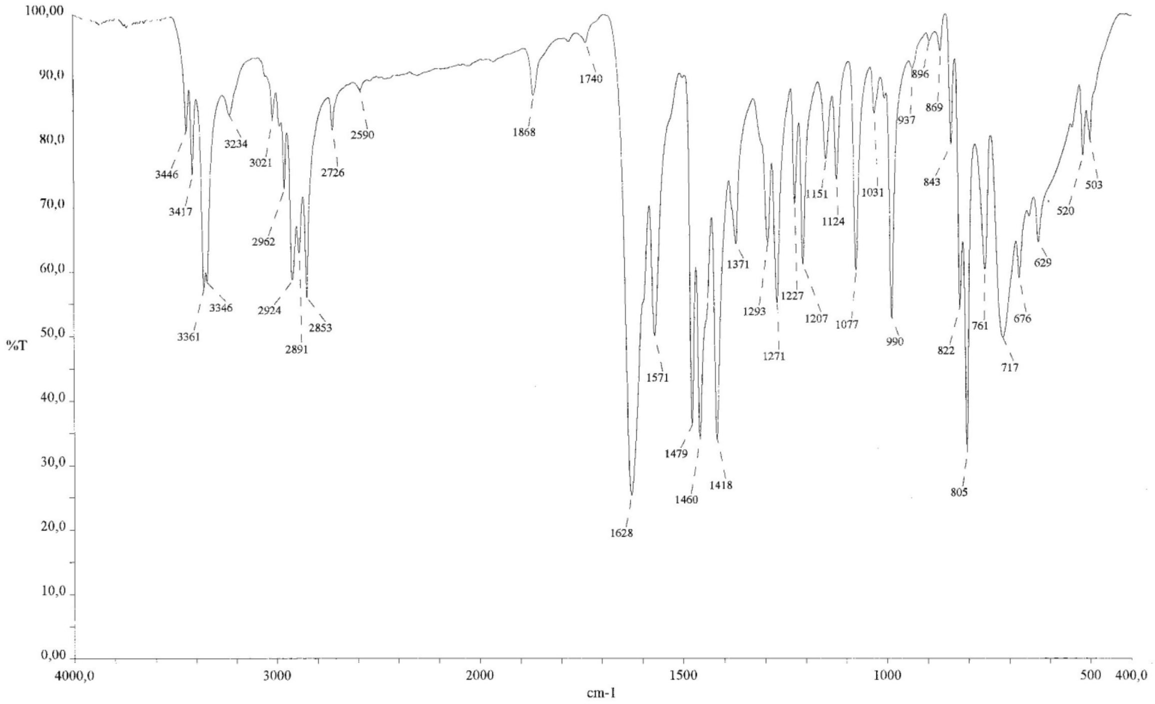

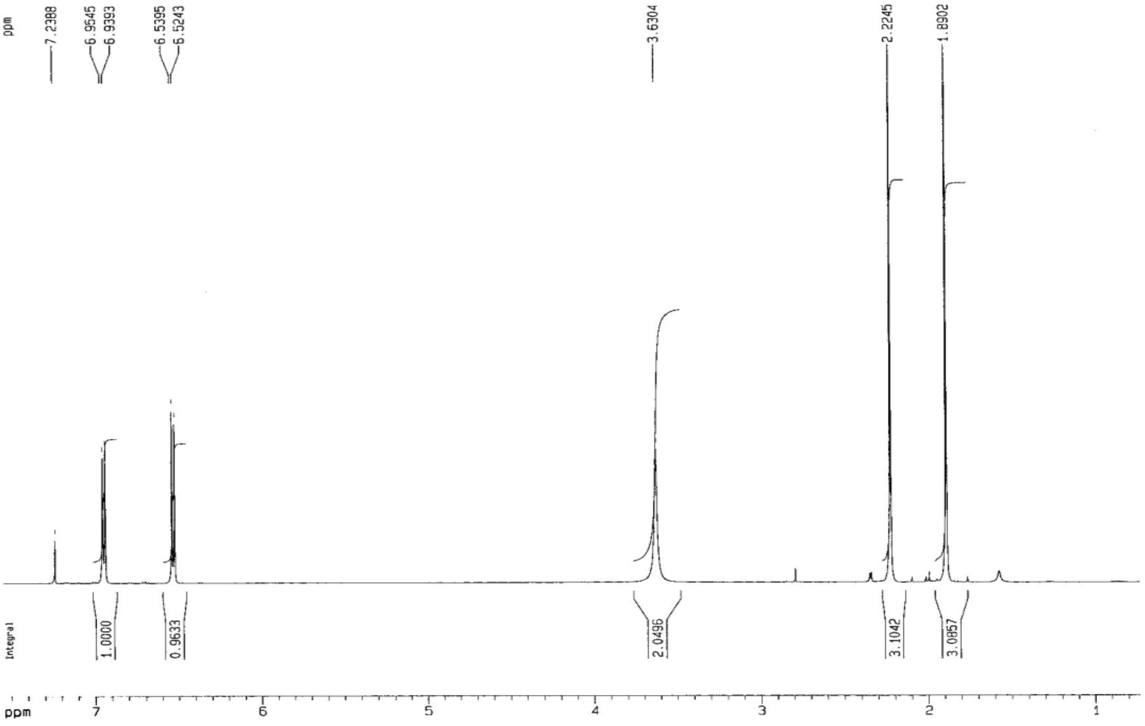

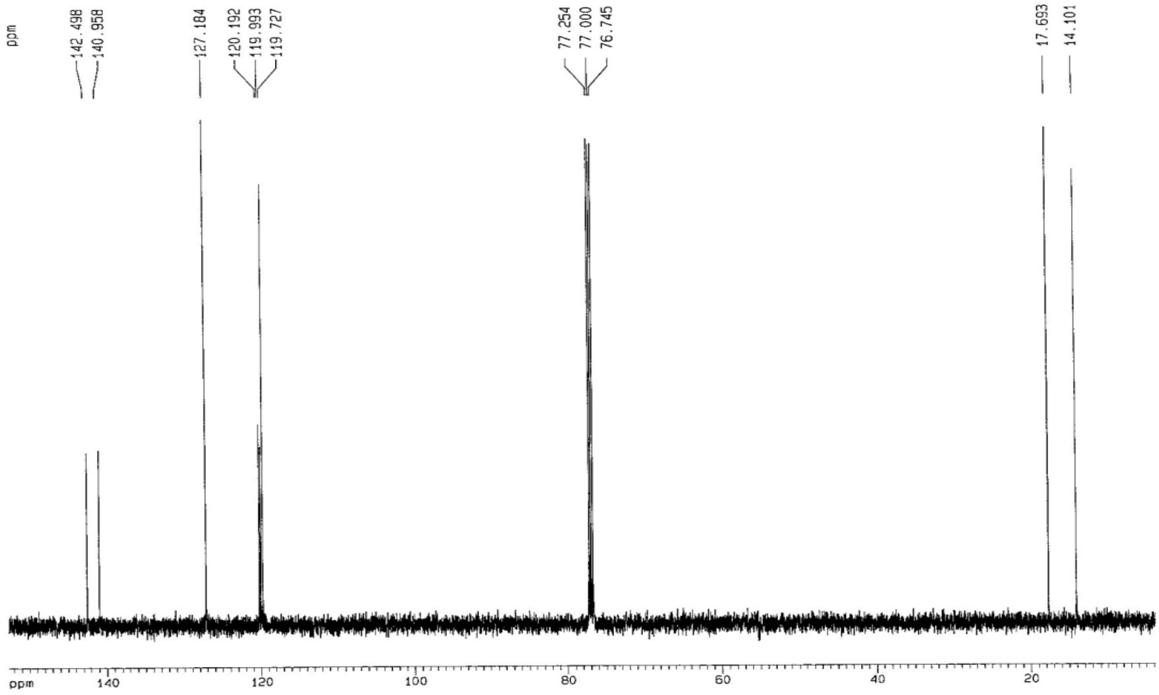
ppm
142.498
140.958
127.184
120.192
119.993
119.727
77.254
77.000
76.745
17.693
14.101
ppm
140
120
100
80
60
40
20

molecules

MDPI

2,2', 4,4'-tetramethyl-3,3'-dimethoxybiphenyl (9a)

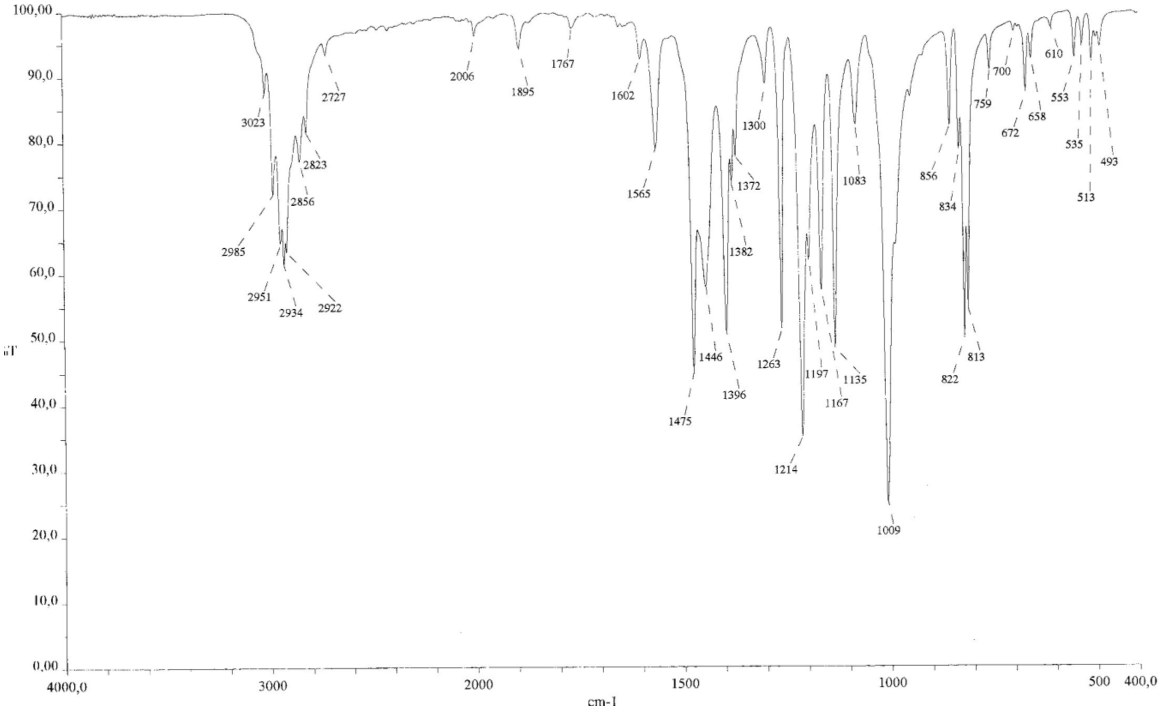

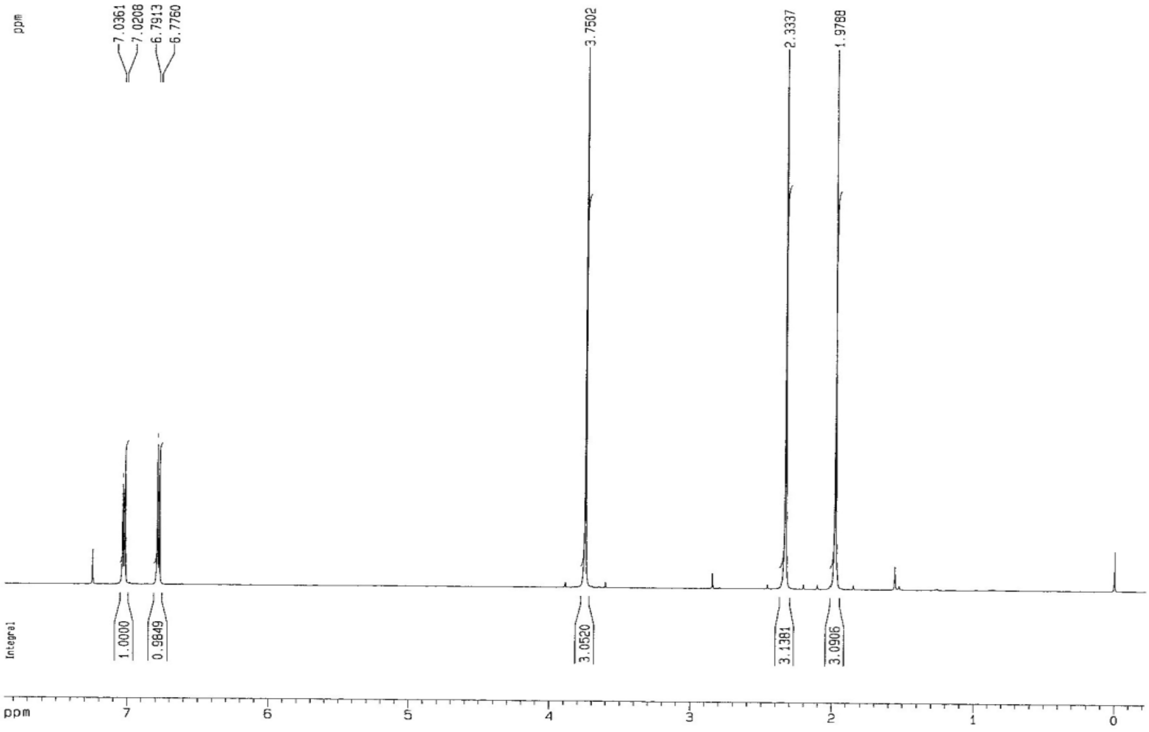

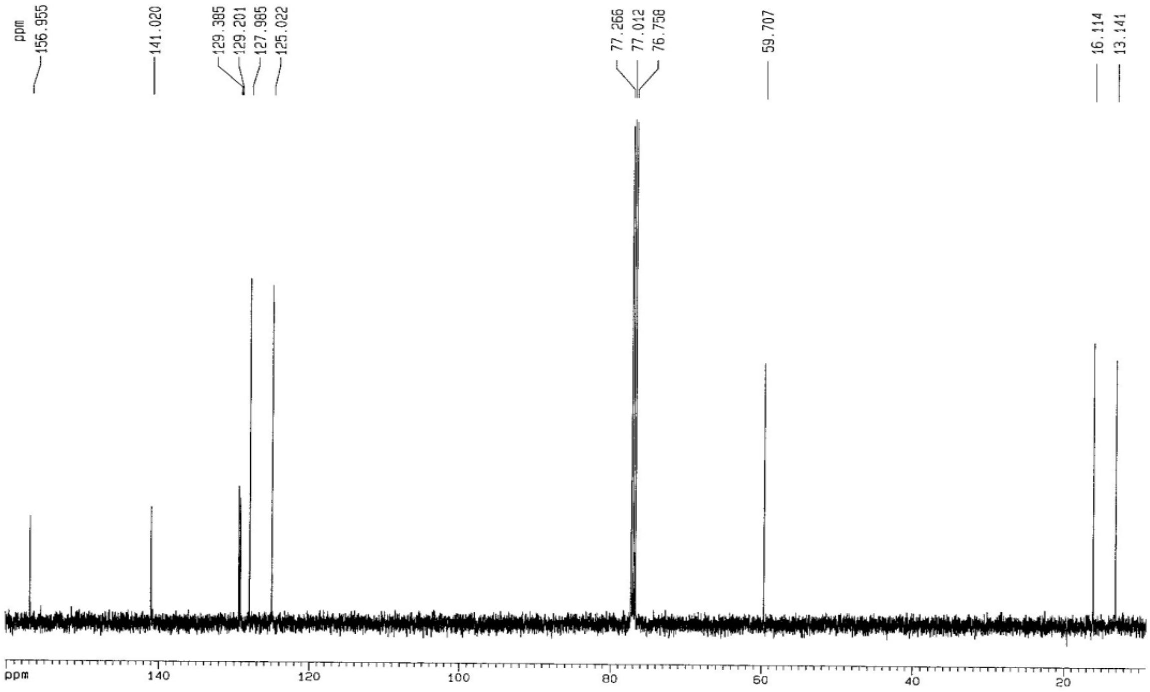
ppm
156.955
141.020
129.385
129.201
127.985
125.022
77.266
77.012
76.758
59.707
16.114
13.141
ppm
140
120
100
80
60
40
20

4,4', 6,6'-tetramethyl-5,5'-diamino-2,2'-diiodobiphenyl (5)

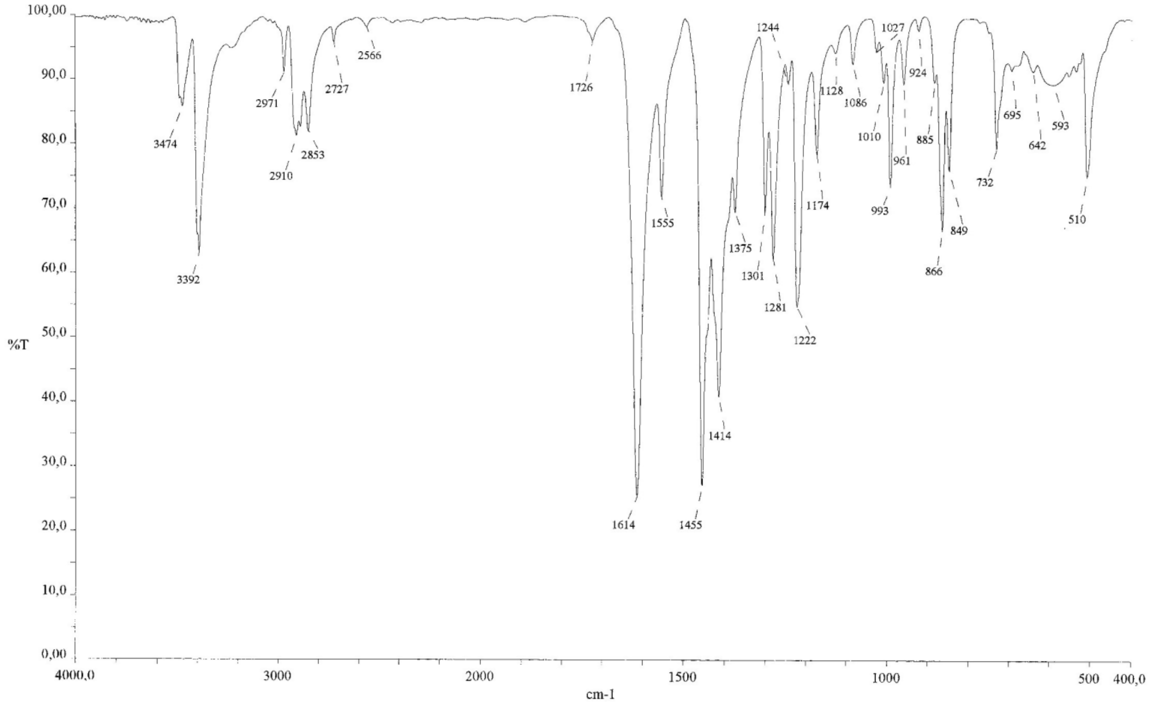

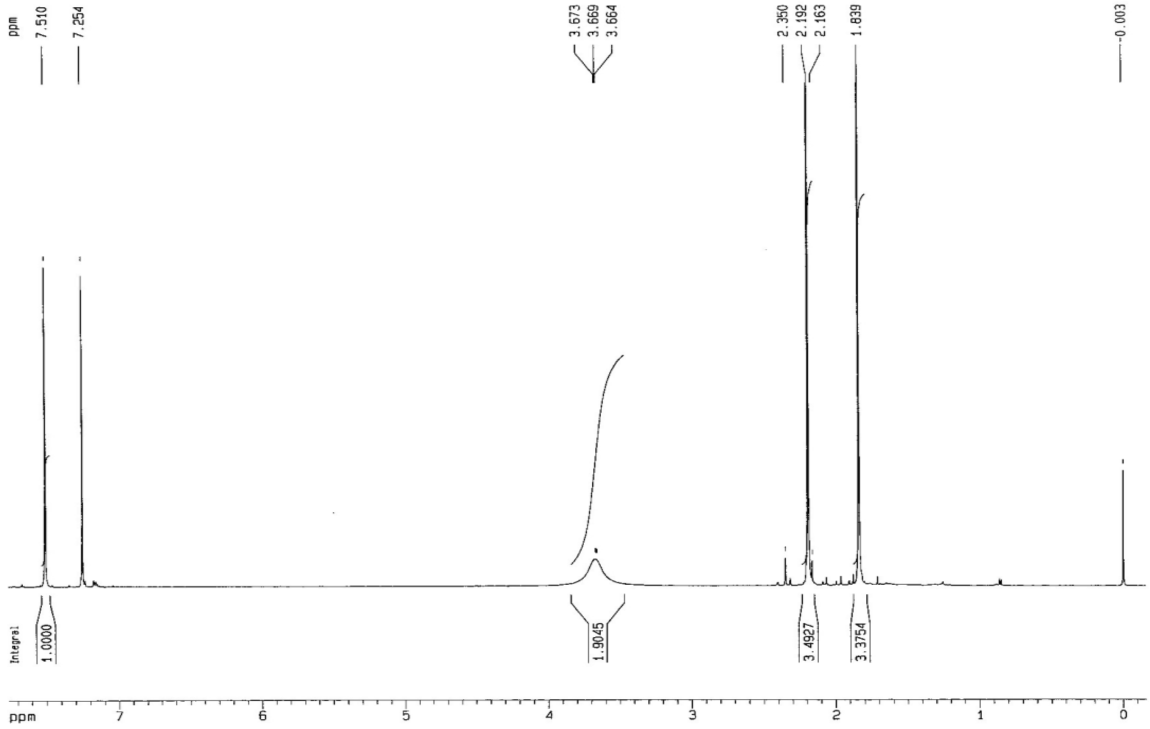

molecules MDPI

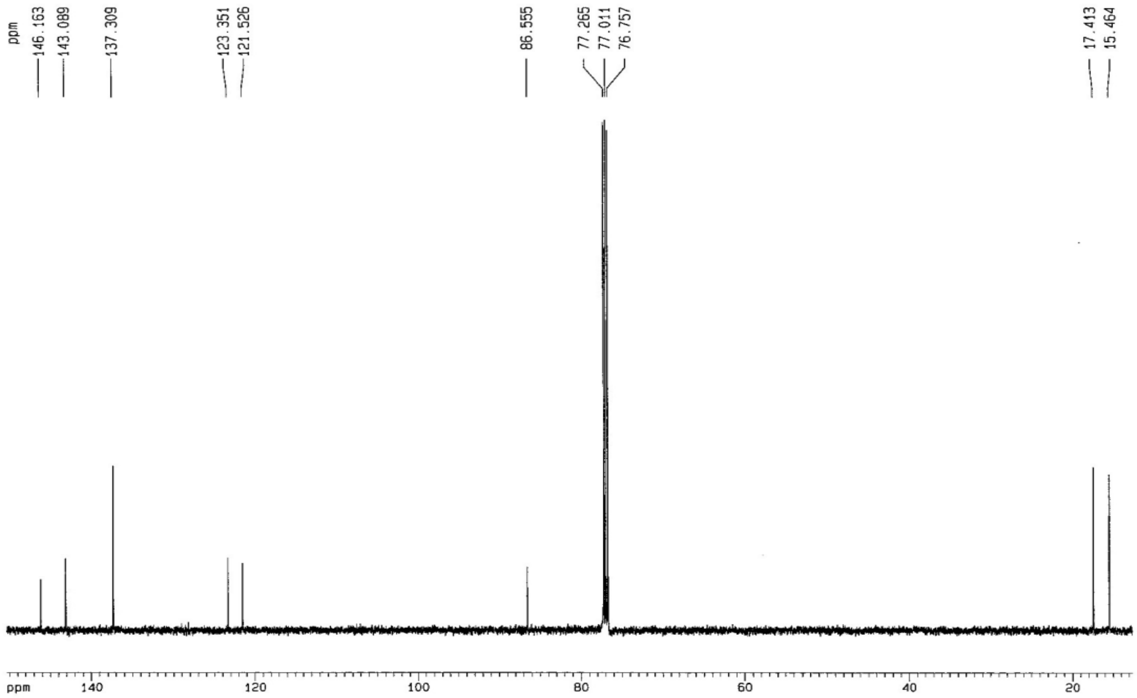

N, N, N',N'-4,4',6,6'-octamethyl-5,5'-diamino-2,2'-diiodobiphenyl (10b)

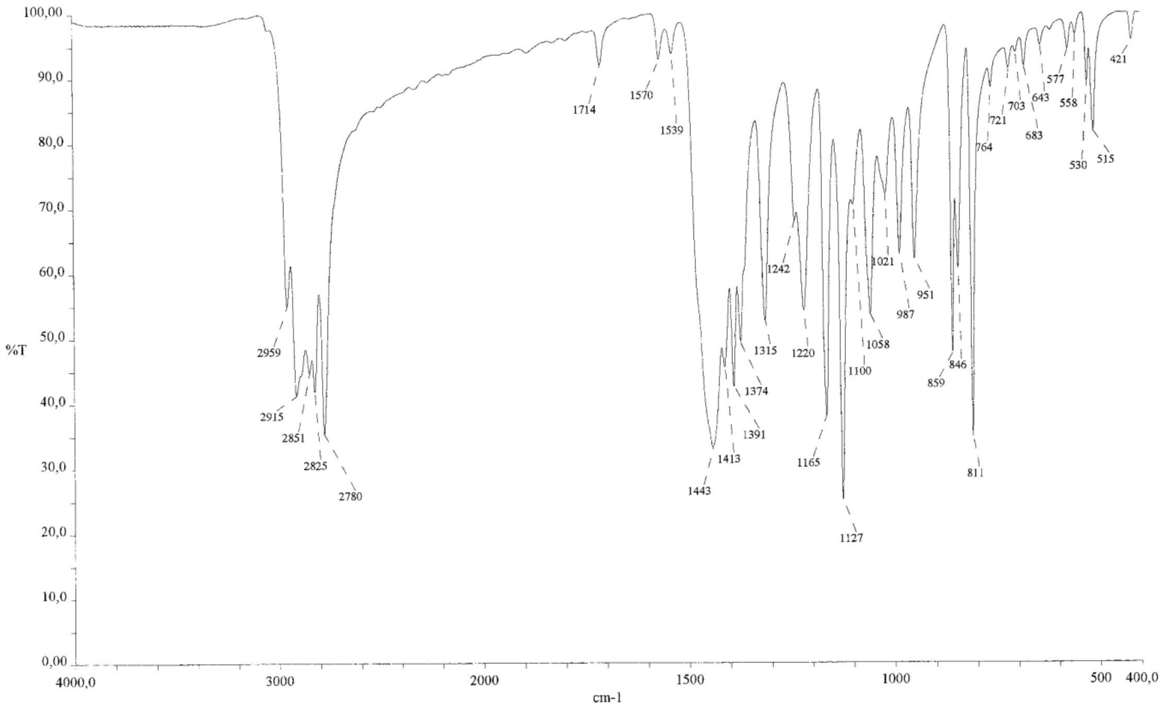

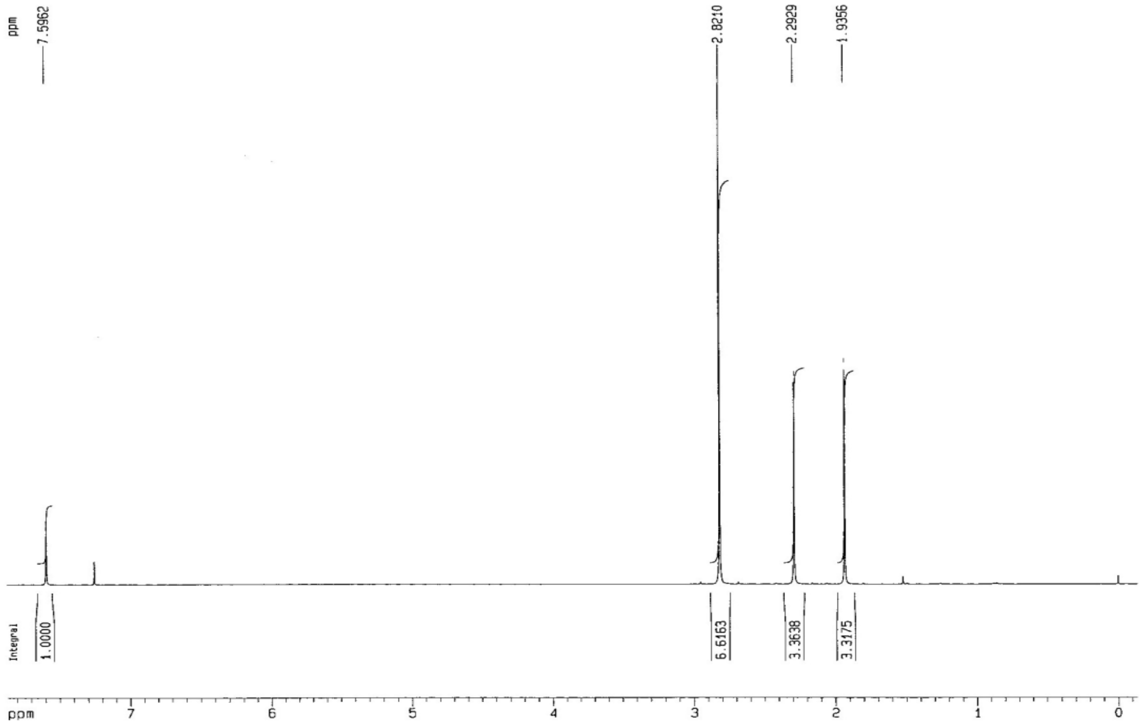

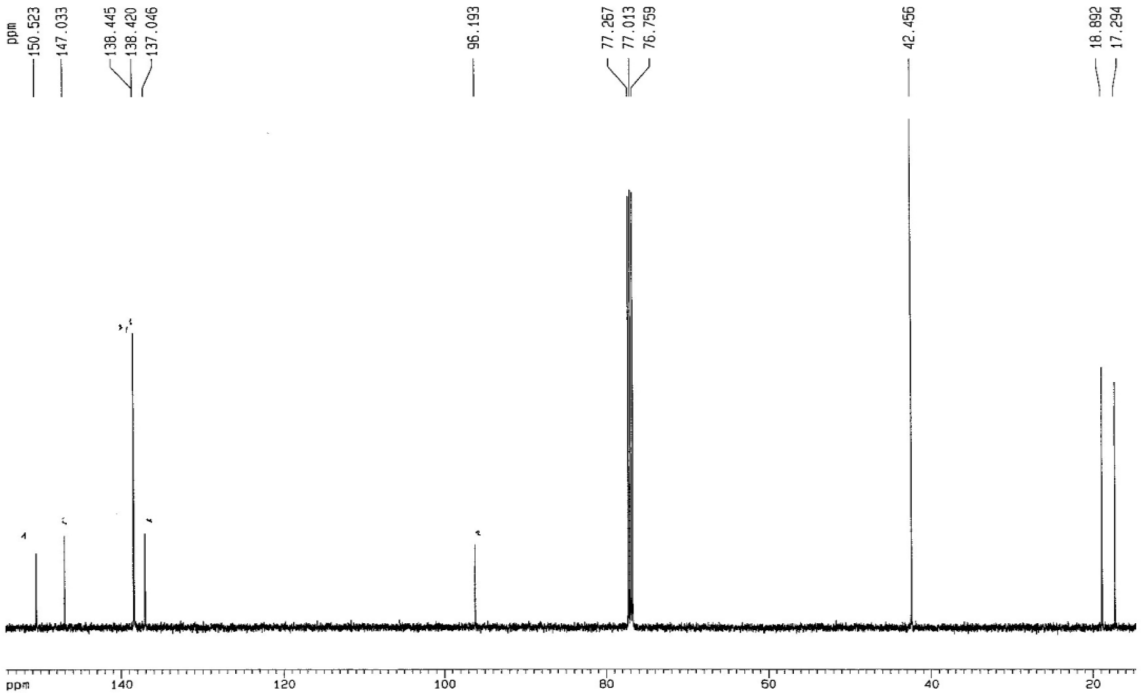
ppm
150.523
147.033
138.445
138.420
137.046
96.193
77.267
77.013
76.759
42.456
18.892
17.294
ppm
140
120
100
80
60
40
20

molecules

MDPI

N, N, N', N'-tetrabutyl-4,4',6,6'-tetramethyl-5,5'-diamino-2,2'-diiodobiphenyl (10e)

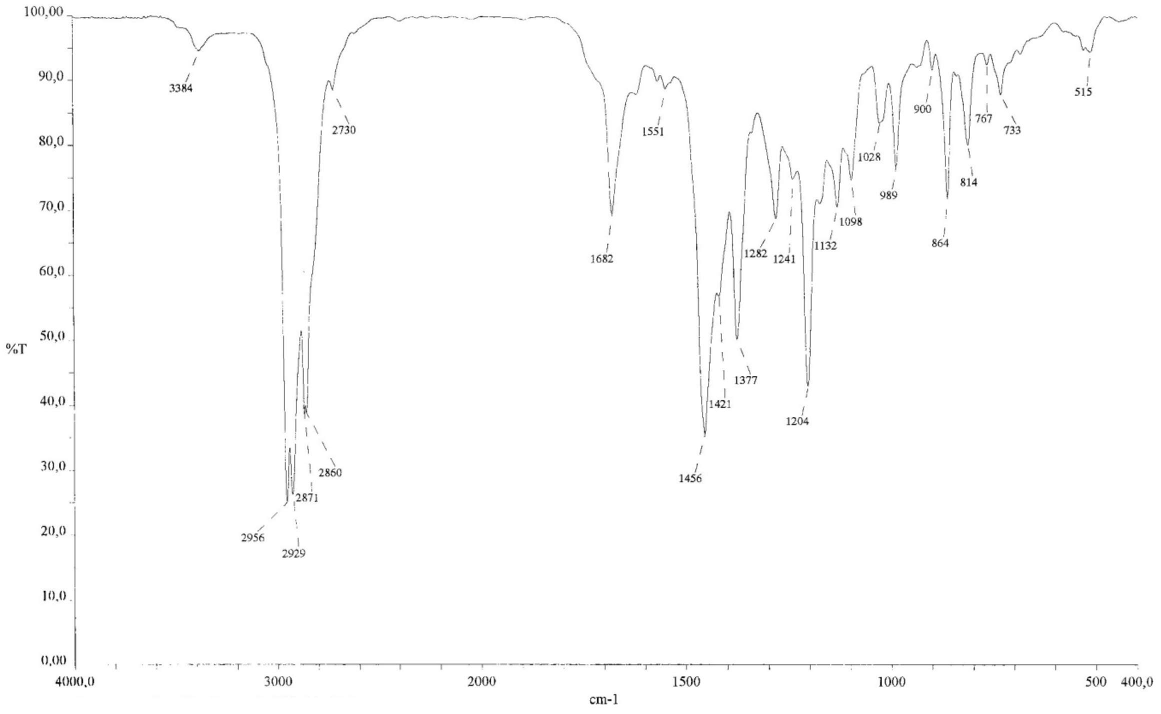

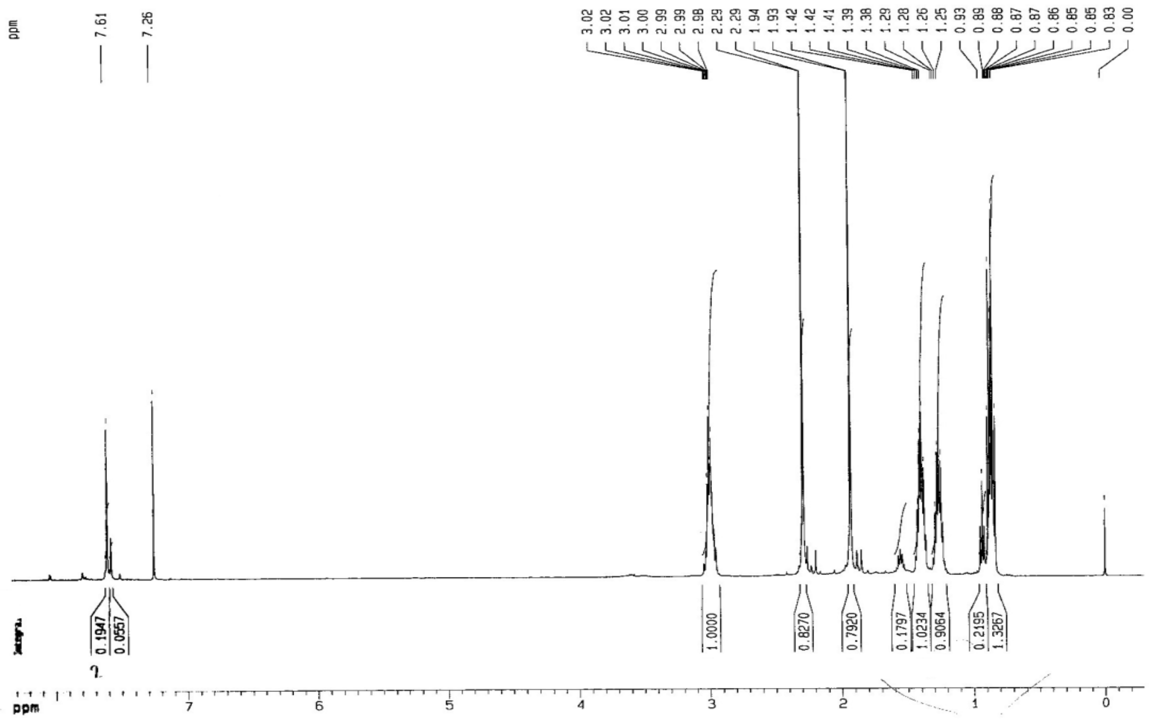

molecules

MDPI

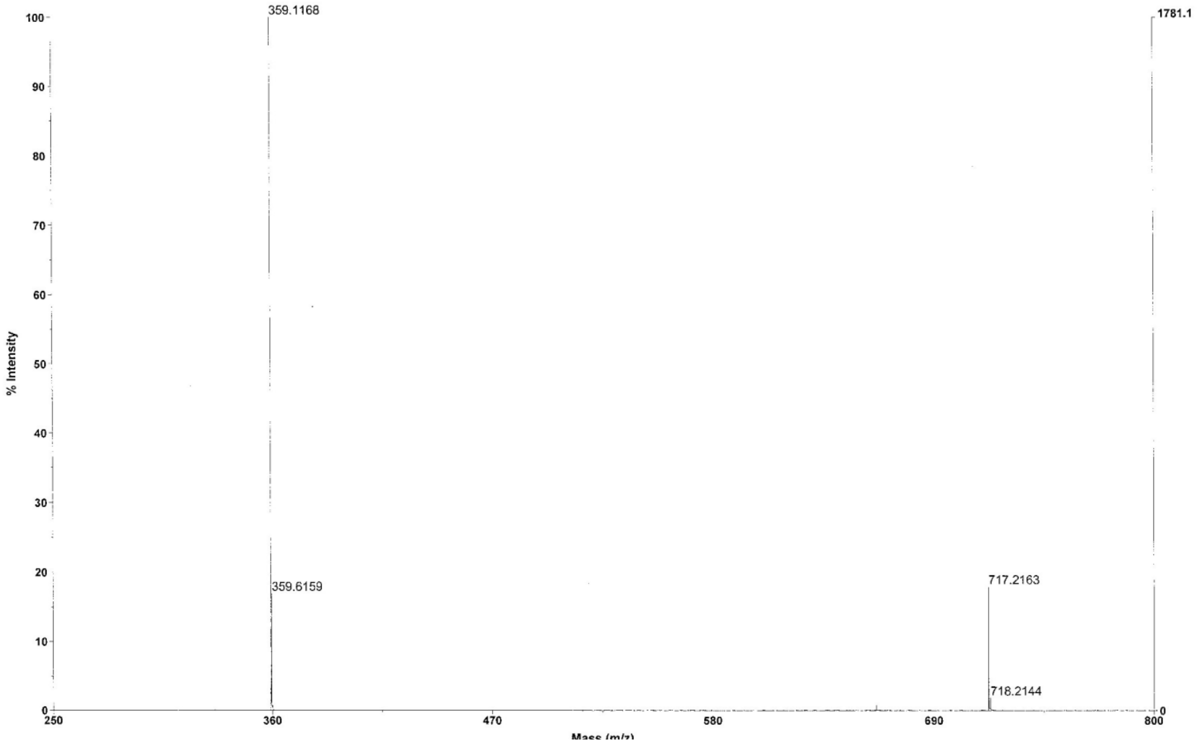

molecules

MDPI

***(S)*-4,4',6,6'-tetramethyl-2,2'-diiodobiphenyl (10d)**

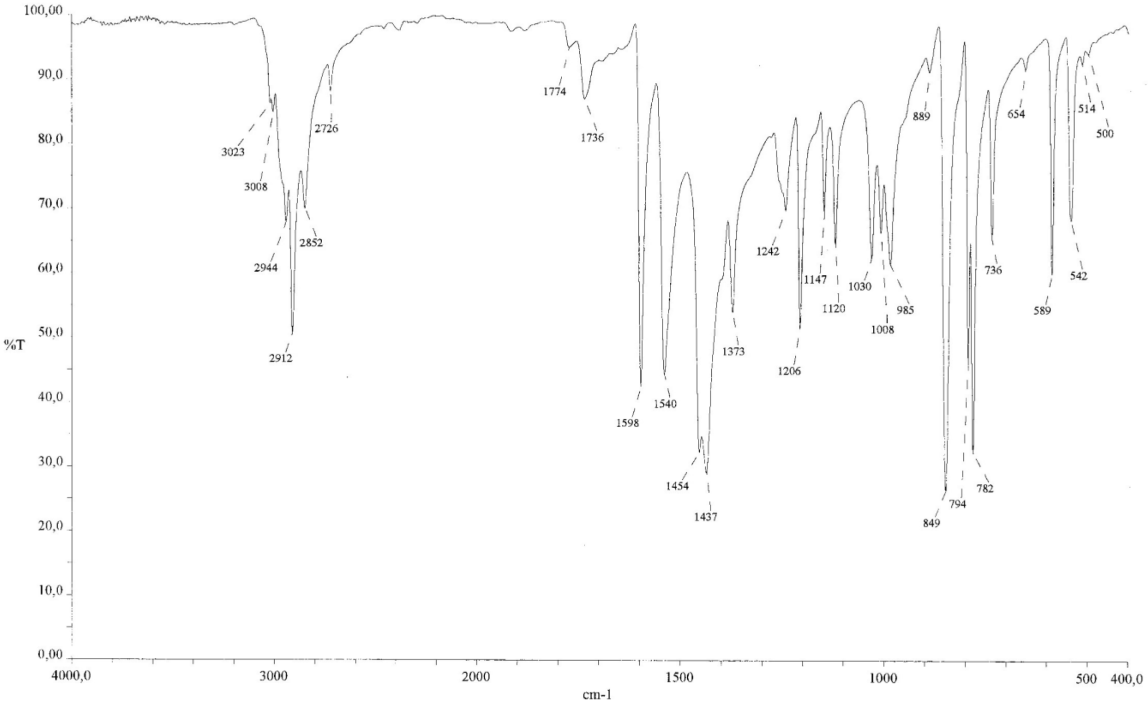

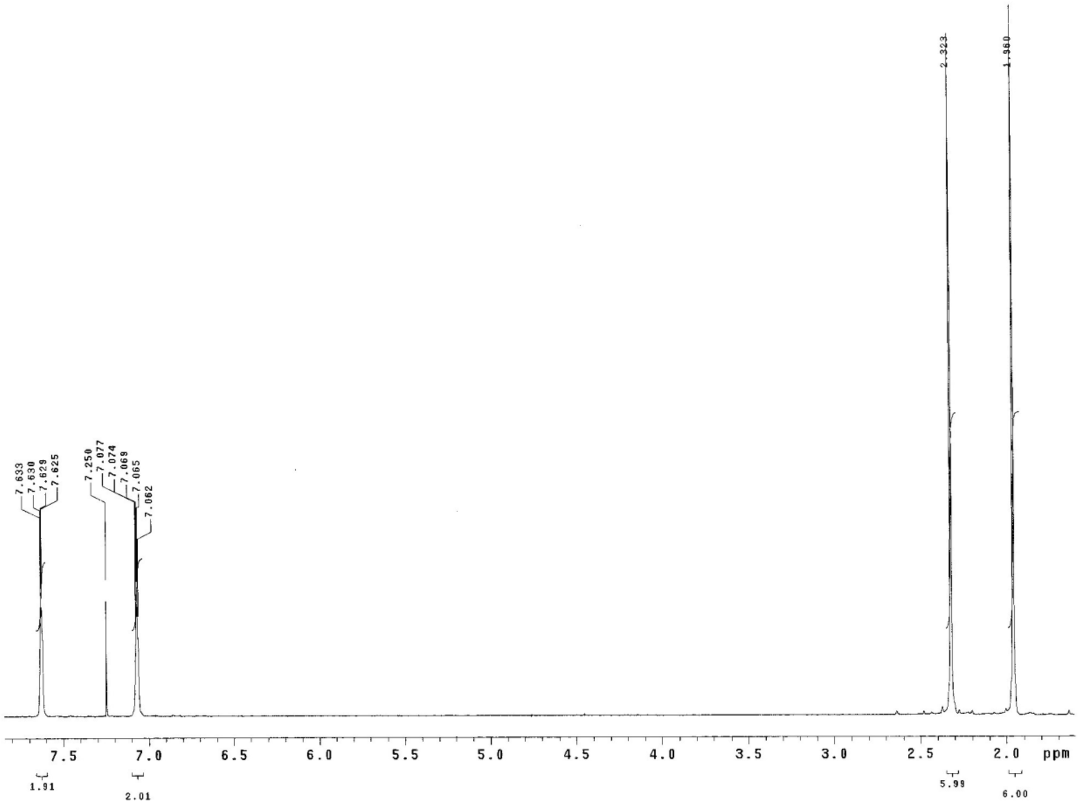

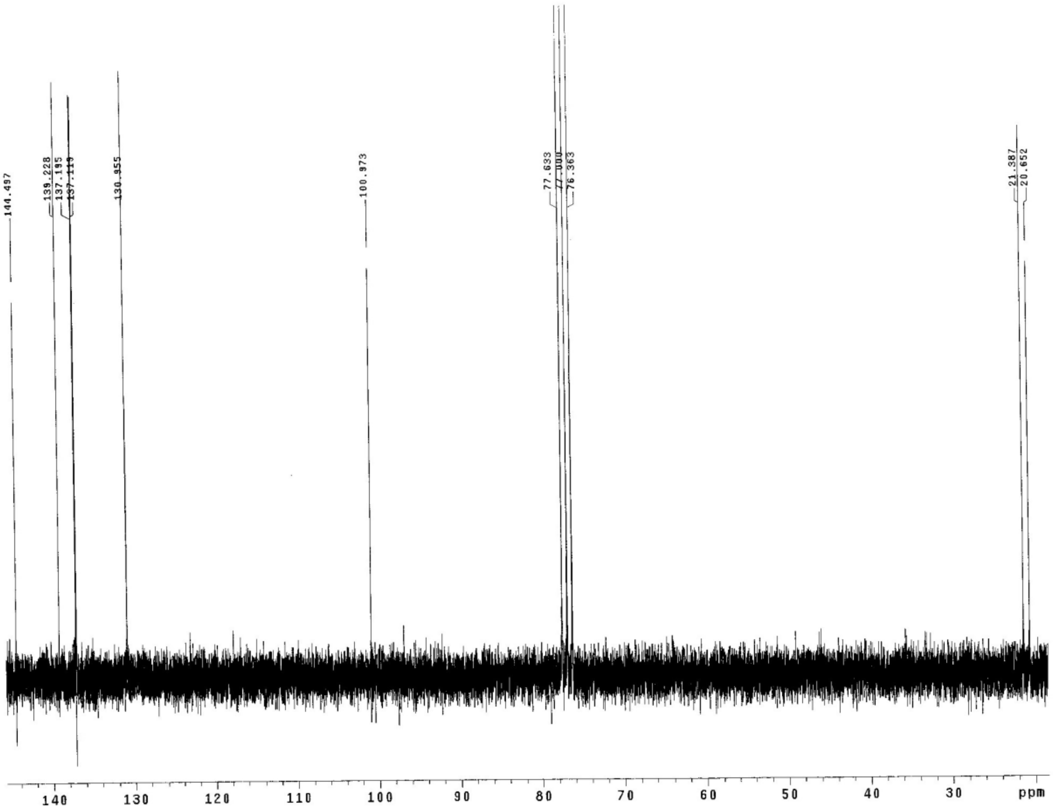
144.497
139.228
137.195
137.119
130.955
100.973
77.633
77.000
76.363
21.387
20.652
140
130
120
110
100
90
80
70
60
50
40
30
ppm

4,4',6,6'-tetramethyl-5,5'-dimethoxy-2,2'-diiodobiphenyl (10a)

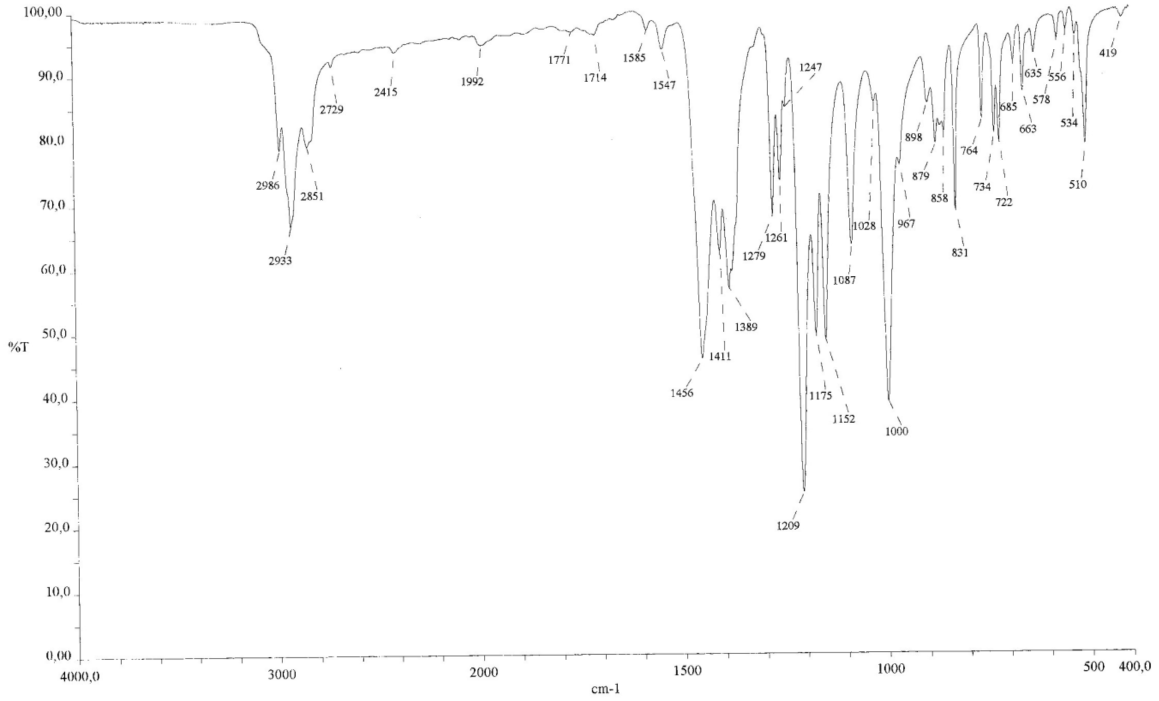

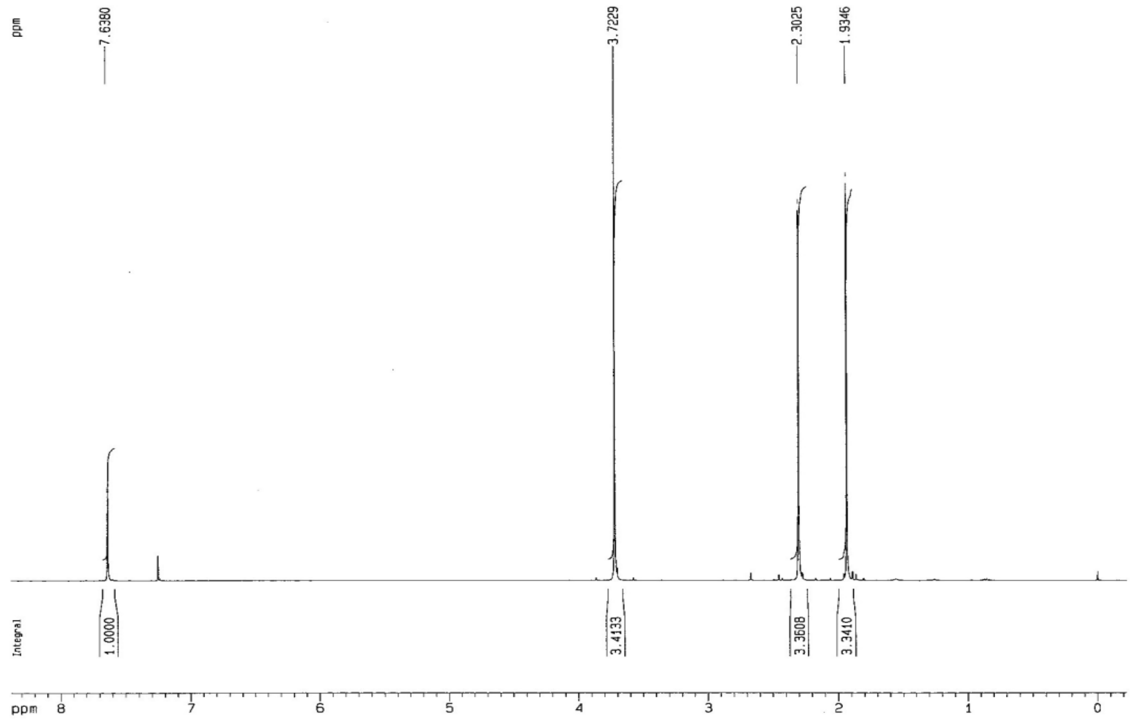

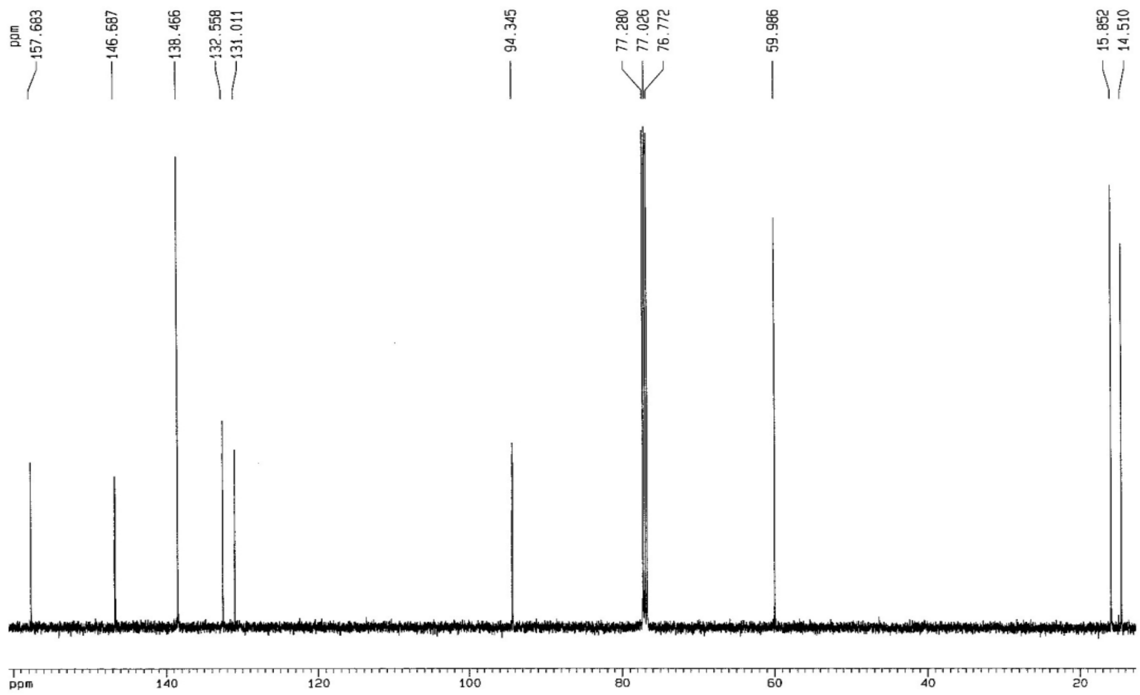
ppm
157.683
146.687
138.466
132.558
131.011
94.345
77.280
77.026
76.772
59.986
15.852
14.510
ppm
140
120
100
80
60
40
20

molecules

MDPI

4,4',6,6'-tetramethyl-5,5'-dichloro-2,2'-diiodobiphenyl (10c)

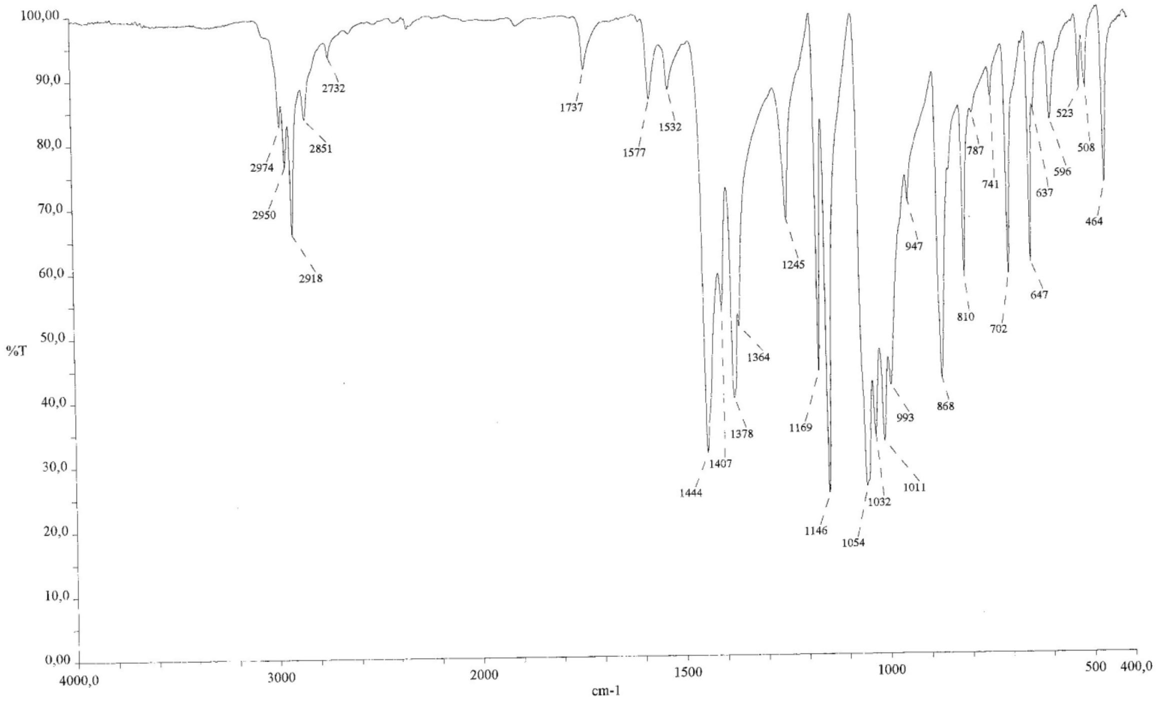

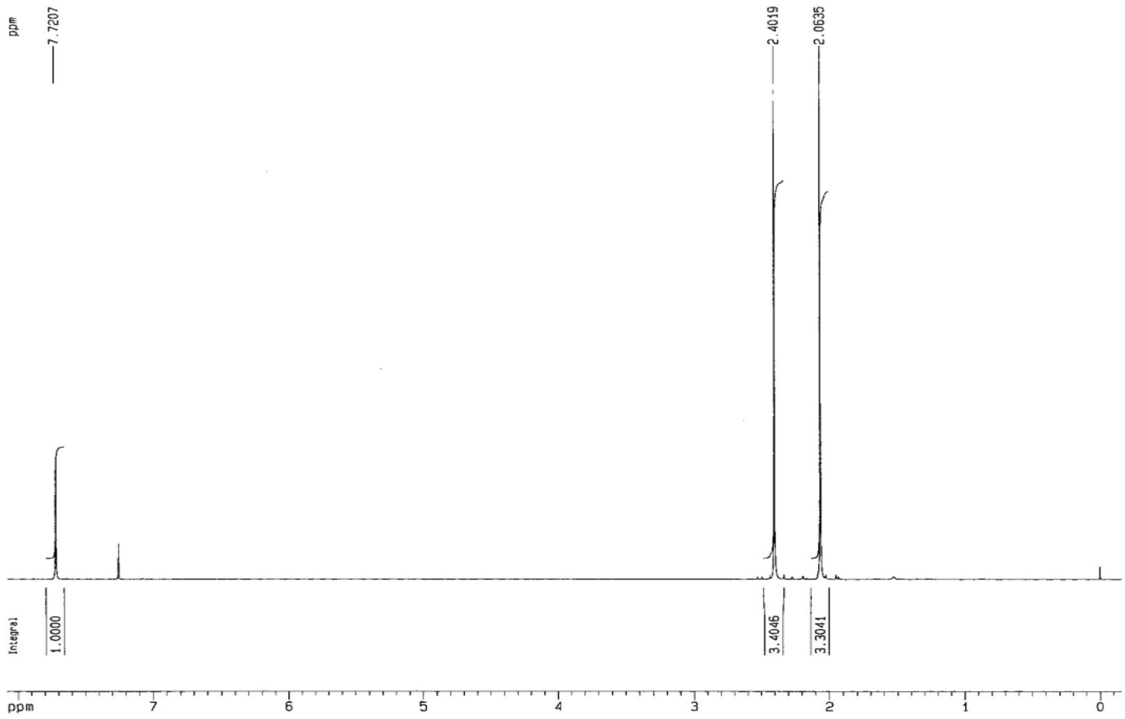

molecules

MDPI

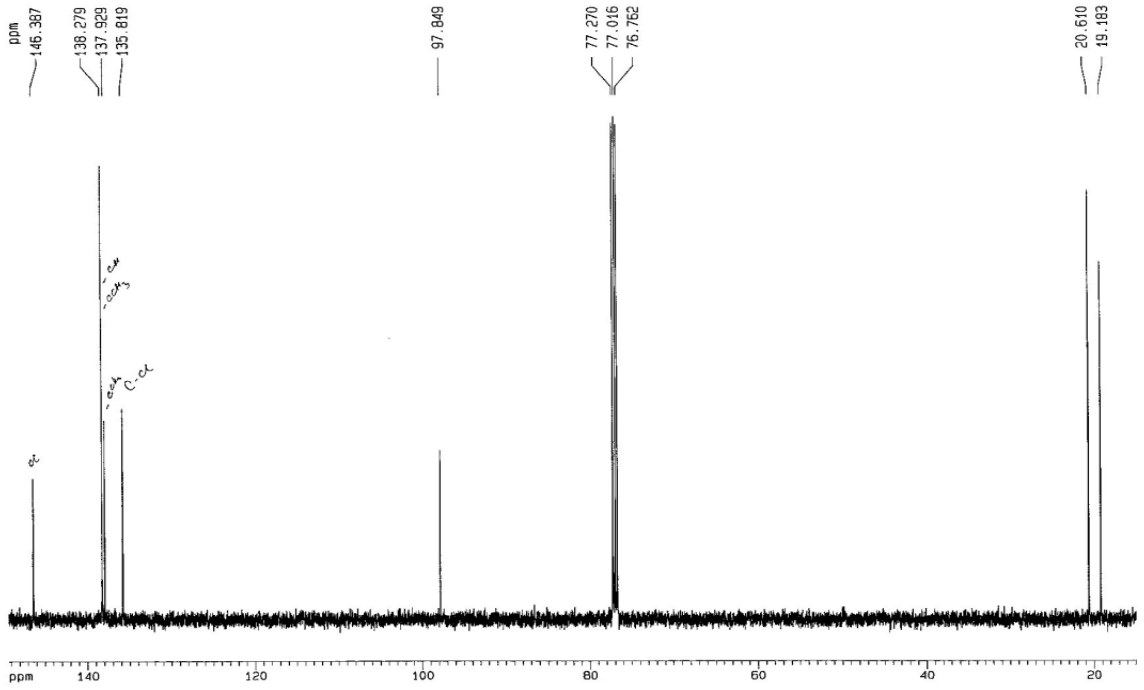

molecules

MDPI

(5,5'-dimethoxy-4,4',6,6'-tetramethylbiphenyl-2,2'-diyl)bis(diphenylphosphane) dioxide (11a):

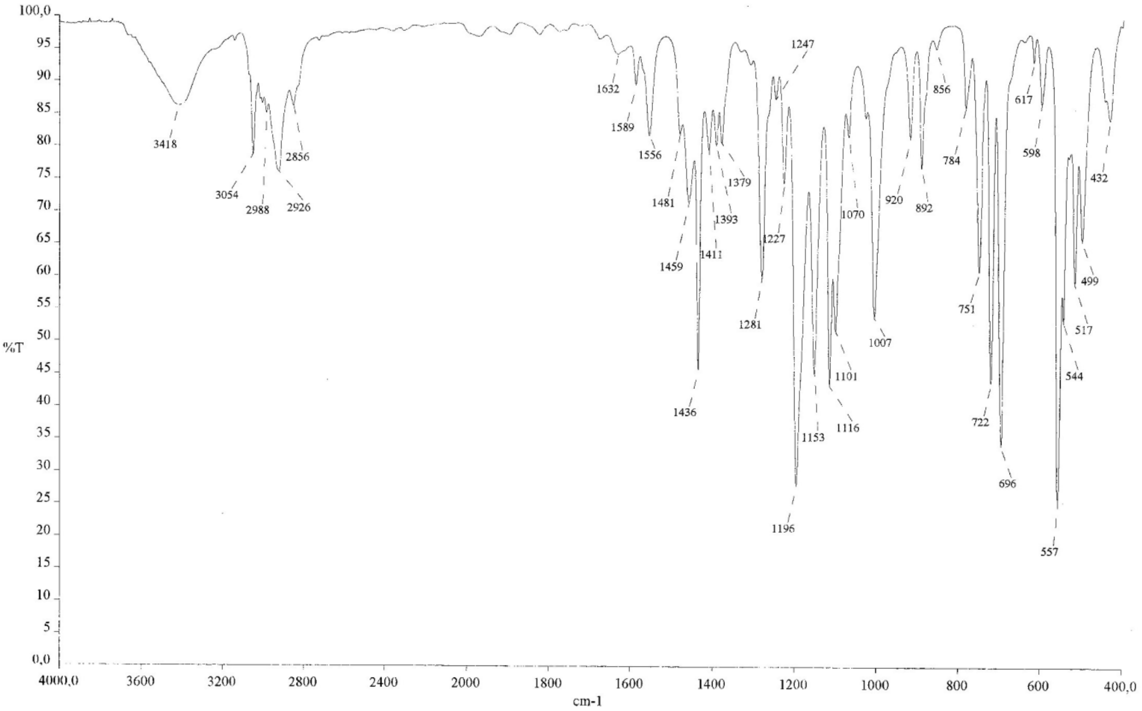

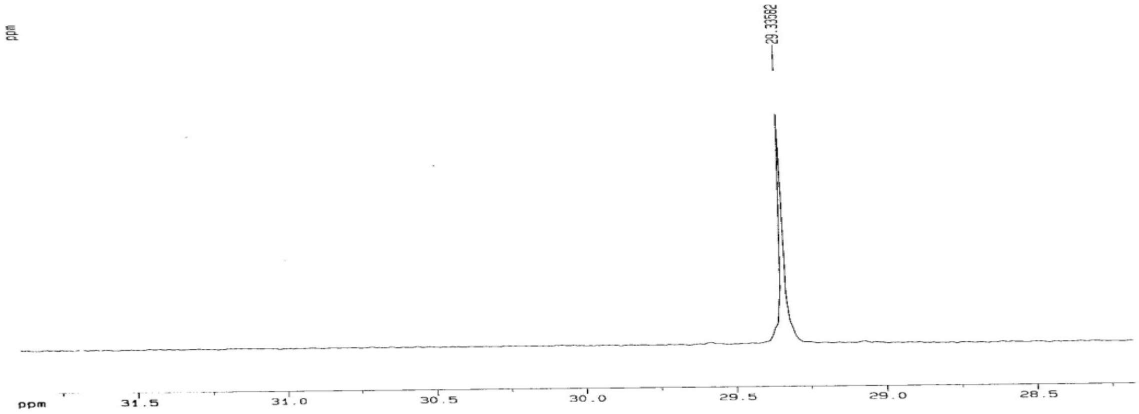

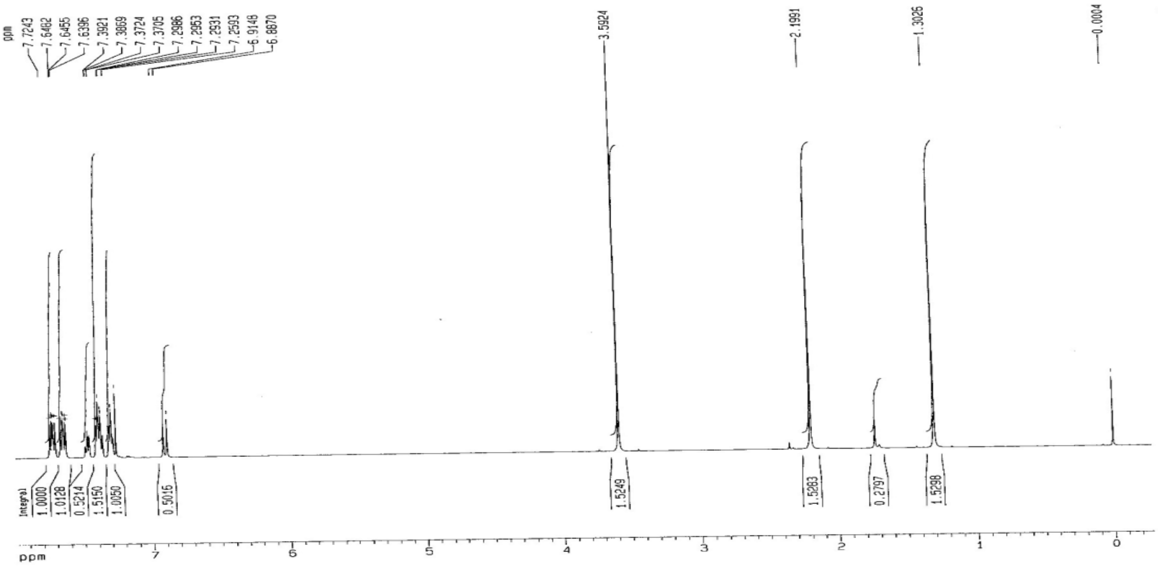
ppm
7.7243
7.6462
7.6455
7.6396
7.3921
7.3869
7.3724
7.3705
7.2986
7.2953
7.2931
7.2593
6.9148
6.8870
3.5924
2.1991
1.3026
0.0004
Integral
1.0000
1.0128
0.5214
1.5150
1.0050
0.5016
1.5249
1.5283
0.2797
1.5298
ppm
7
6
5
4
3
2
1
0

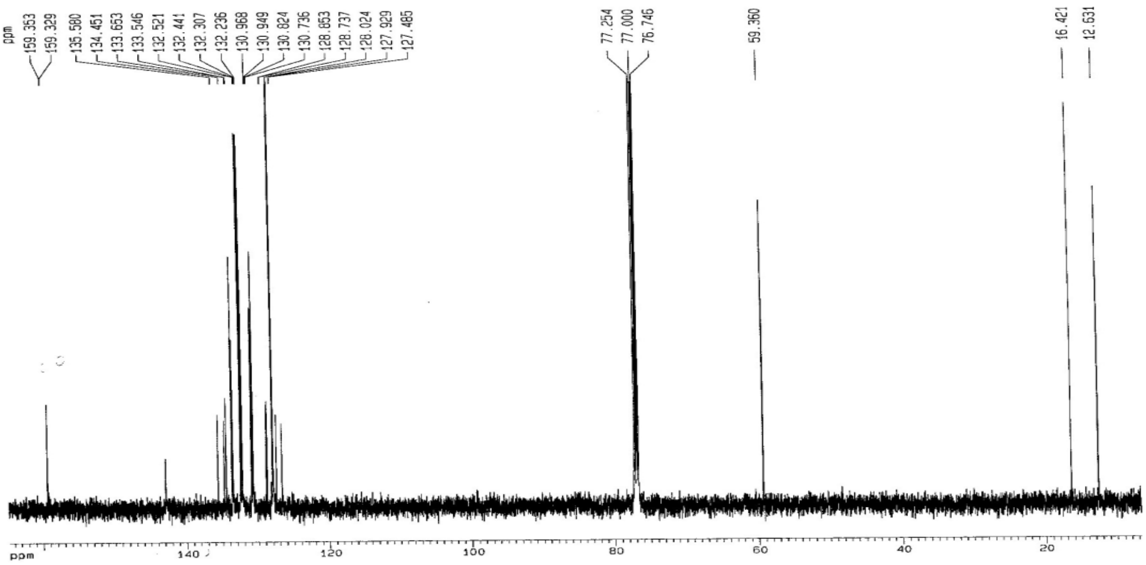
ppm
159.353
159.329
135.580
134.451
133.653
133.546
132.521
132.441
132.307
132.236
130.968
130.949
130.824
130.736
128.853
128.737
128.024
127.929
127.485
77.254
77.000
76.745
59.360
16.421
12.631
ppm
140
120
100
80
60
40
20

molecules

(3',5-dimethoxy-2',4,4',6-tetramethylbiphenyl-2-yl)(diphenyl)phosphane oxide (12a)

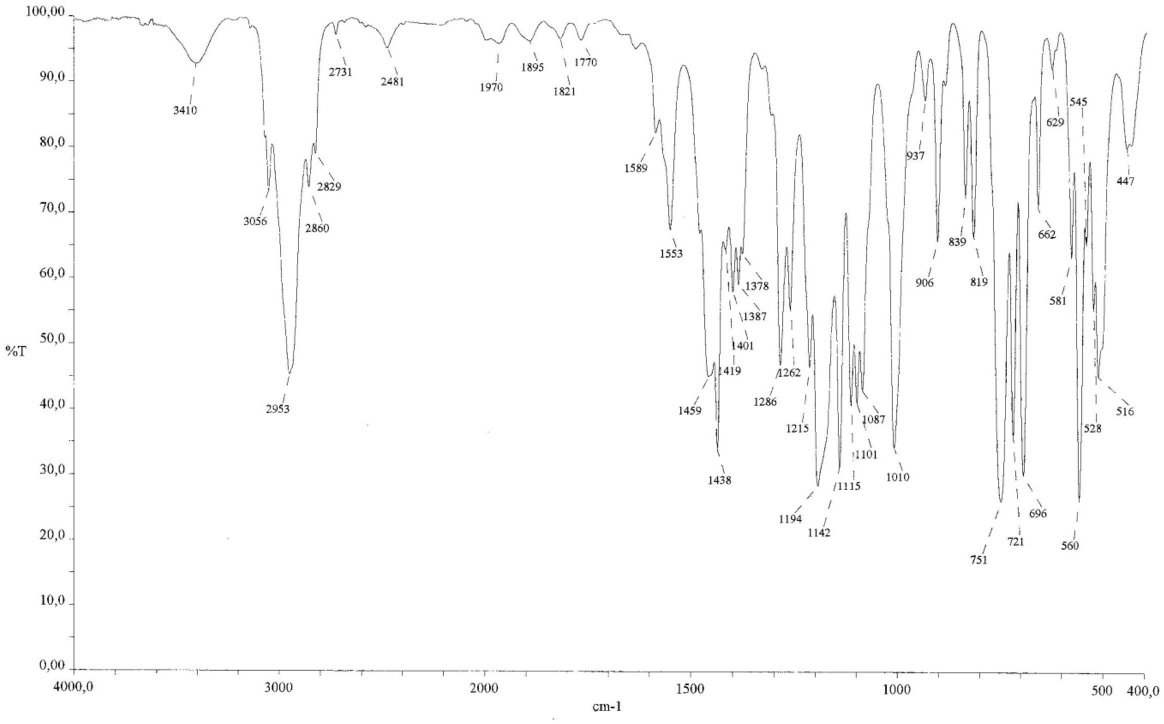

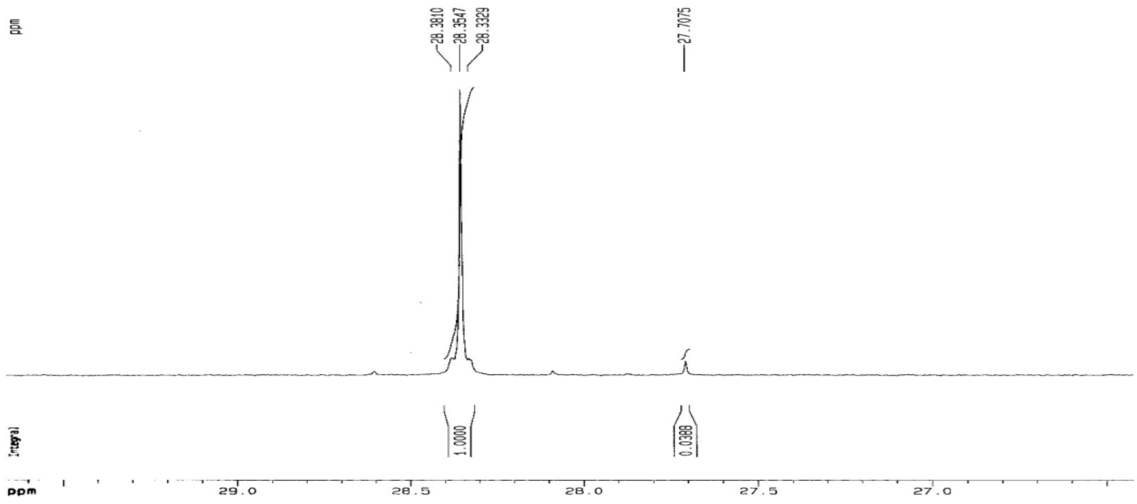

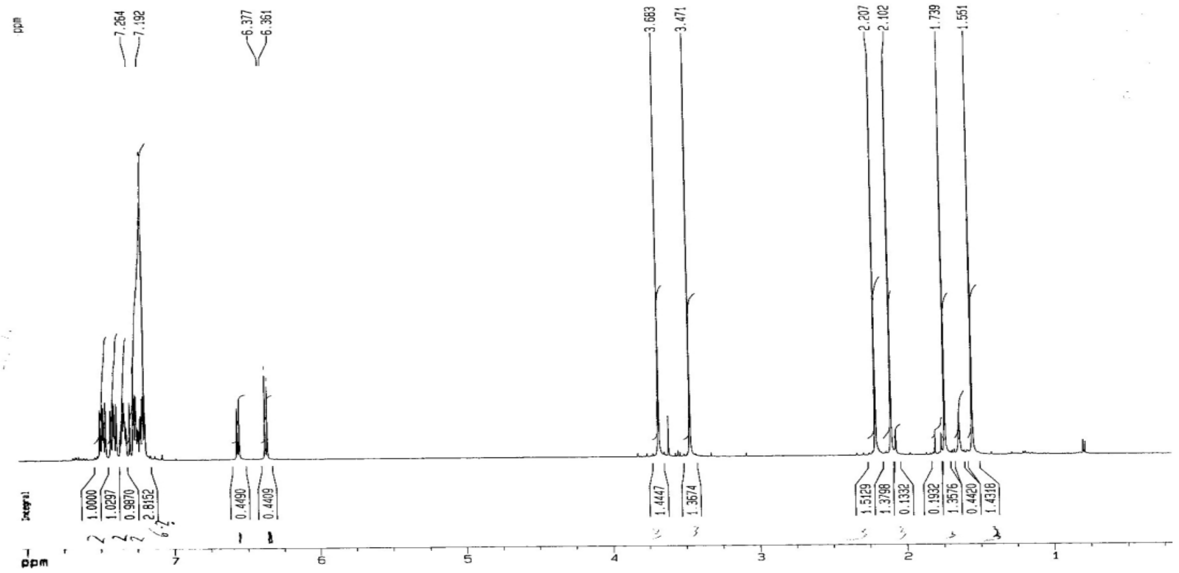
ppm
7.264
7.192
6.377
6.361
3.683
3.471
2.207
2.102
1.739
1.551
Integral
1.0000
1.0297
0.9870
2.8152
0.4490
0.4409
1.4447
1.3674
1.5129
1.3798
0.1332
0.1932
1.3576
0.4420
1.4318
ppm
7
6
5
4
3
2
1

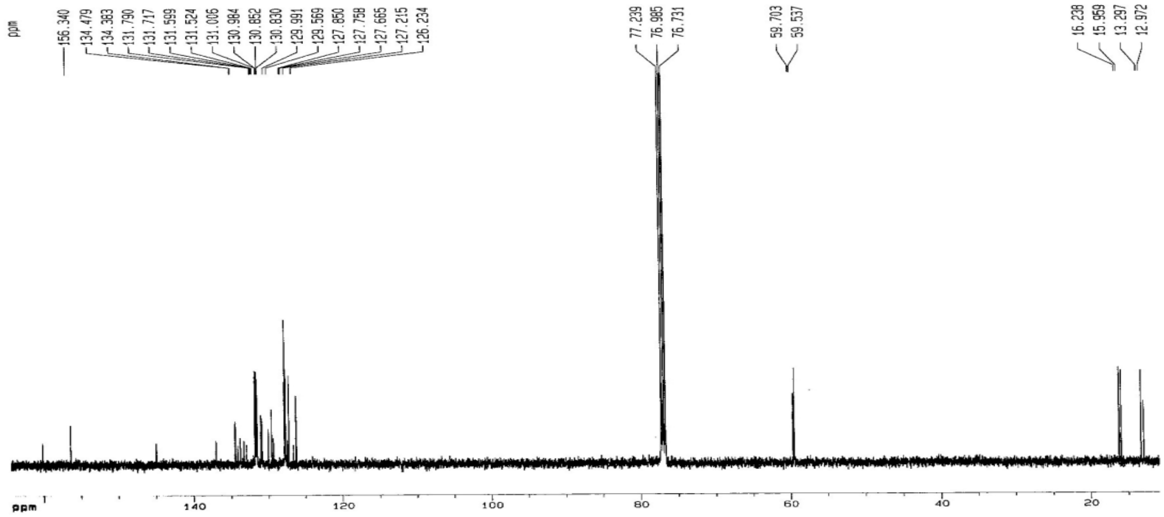
ppm
156.340
134.479
134.383
131.790
131.717
131.599
131.524
131.006
130.984
130.852
130.830
129.991
129.569
127.850
127.758
127.665
127.215
126.234
77.239
76.985
76.731
59.703
59.537
16.238
15.959
13.297
12.972
ppm
140
120
100
80
60
40
20

molecules

MDPI

6,6'-bis(diphenylphosphoryl)-2,2',4,4'-tetramethylbiphenyl-3,3'-diol (11f)

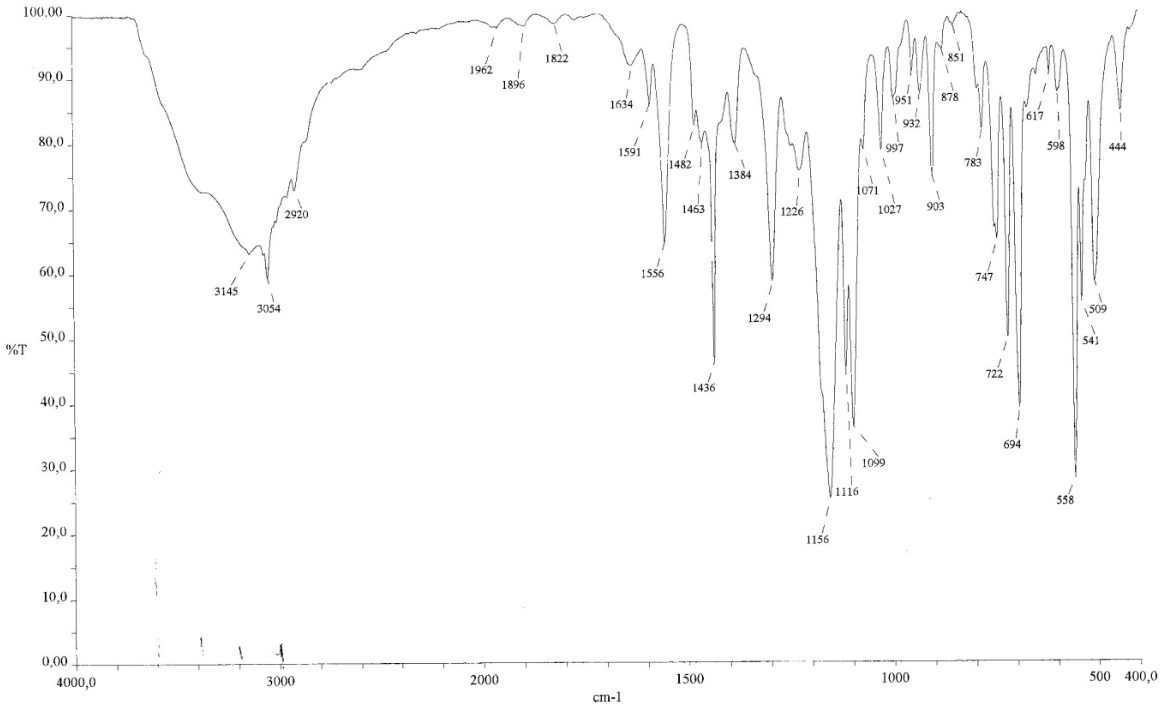

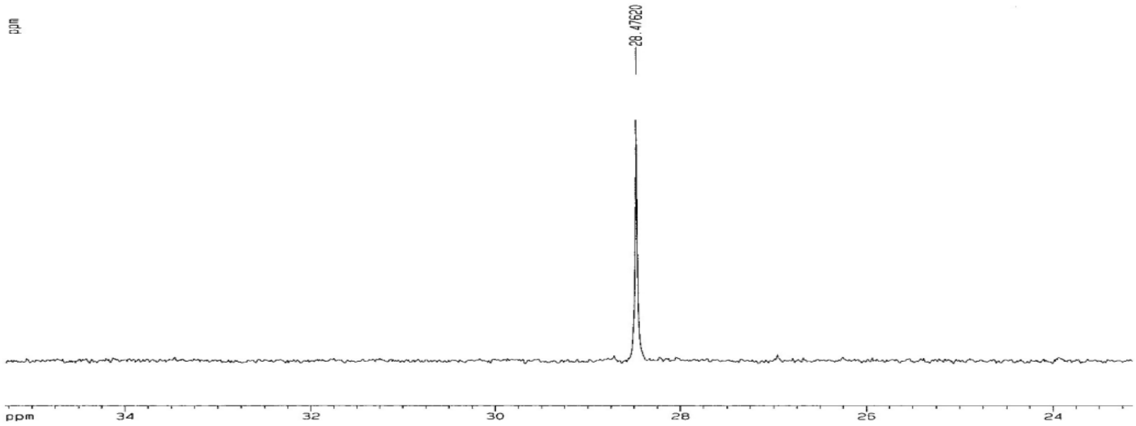

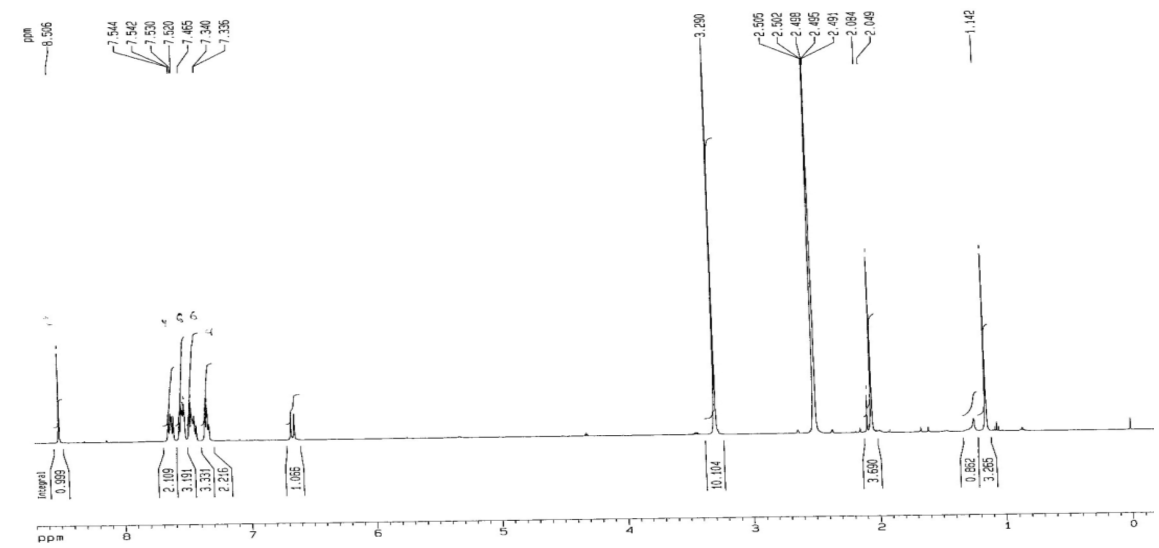

ppm
8.506
7.544
7.542
7.530
7.520
7.465
7.340
7.336
3.290
2.505
2.502
2.498
2.495
2.491
2.084
2.049
1.142
Integral
0.999
2.109
3.191
3.331
2.216
1.066
10.104
3.690
0.862
3.265
ppm
8
7
6
5
4
3
2
1
0

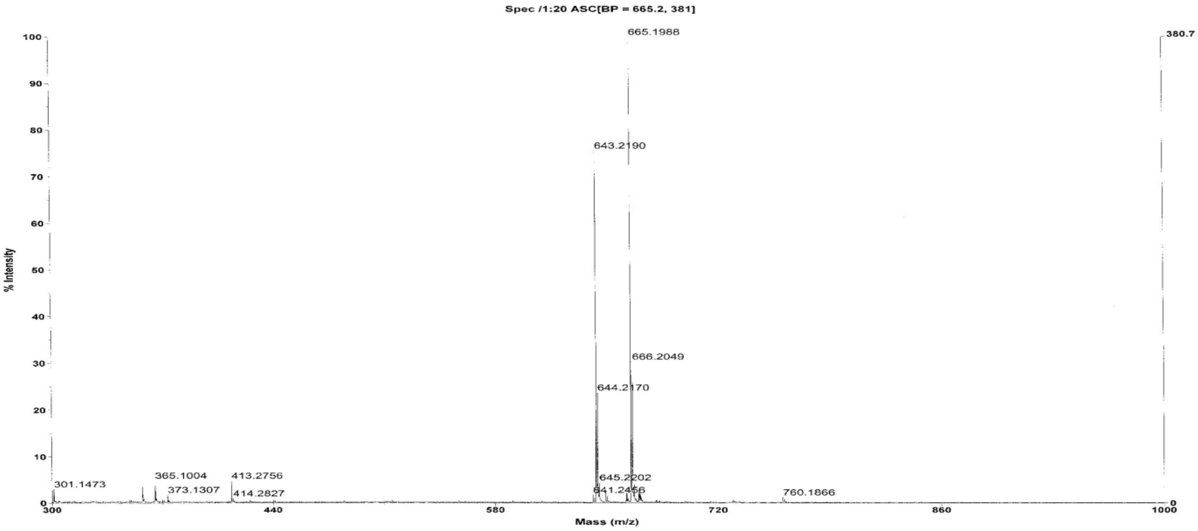

Spec /1:20 ASC[BP = 665.2, 381]
665.1988
380.7
643.2190
666.2049
644.2170
645.2202
641.2456
301.1473
365.1004
373.1307
413.2756
414.2827
760.1866
% Intensity
Mass (m/z)

molecules

MDPI

[5,5'-bis(benzyloxy)-4,4',6,6'-tetramethylbiphenyl-2,2'-diyl]bis(diphenylphosphane) dioxide (11g)

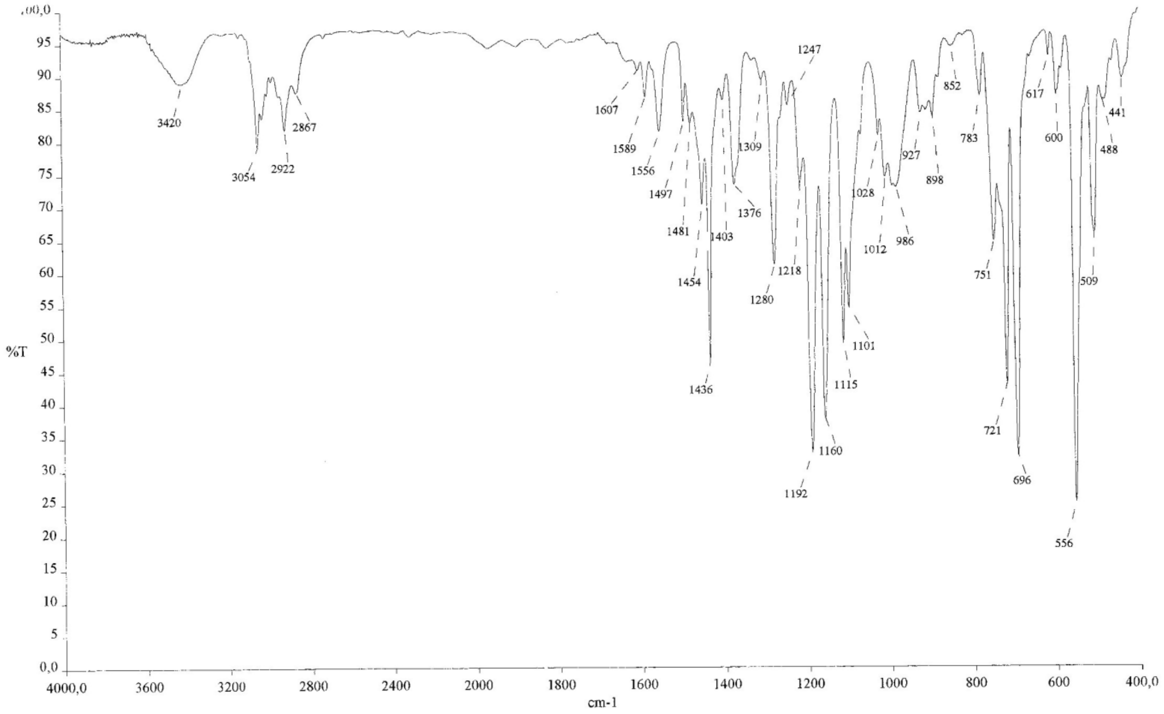

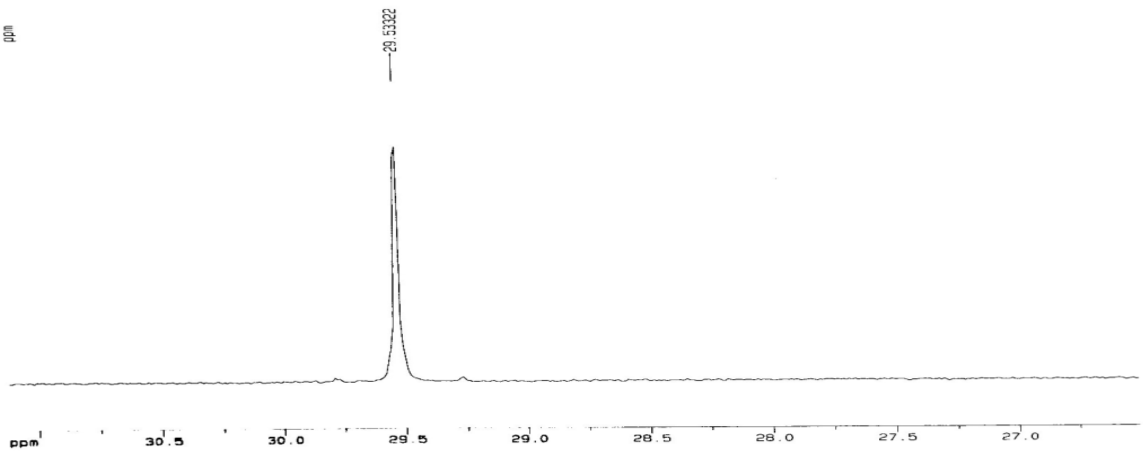

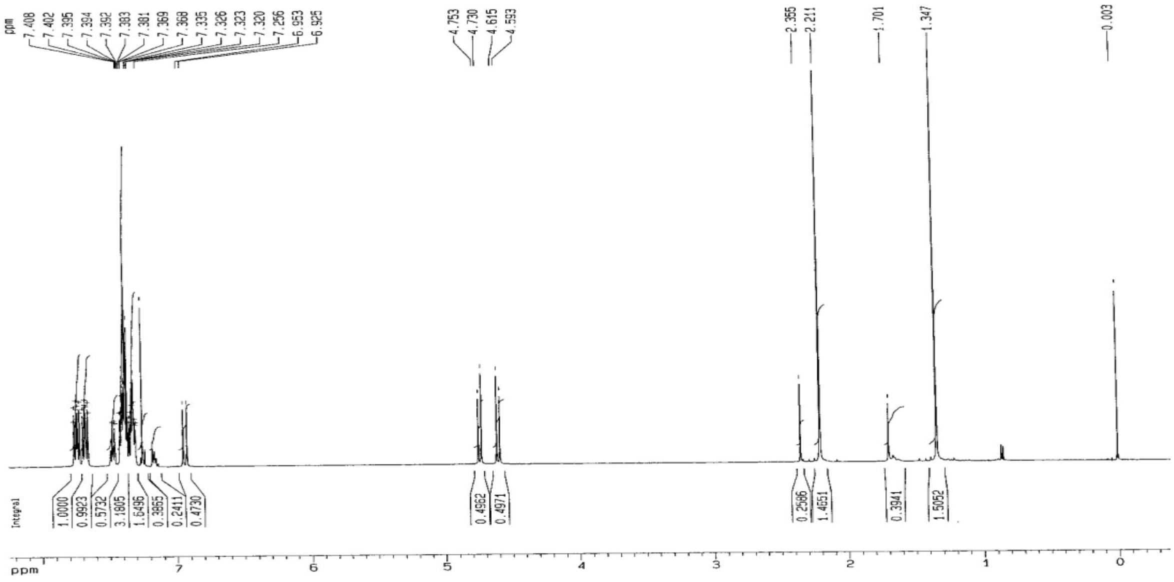
ppm
7.408
7.402
7.395
7.394
7.392
7.383
7.381
7.369
7.368
7.335
7.326
7.323
7.320
7.256
6.953
6.925
4.753
4.730
4.615
4.593
2.355
2.211
1.701
1.347
0.003
Integral
1.0000
0.9923
0.5732
3.1805
1.6496
0.3865
0.2411
0.4730
0.4962
0.4971
0.2596
1.4651
0.3941
1.5052
ppm
7
6
5
4
3
2
1
0

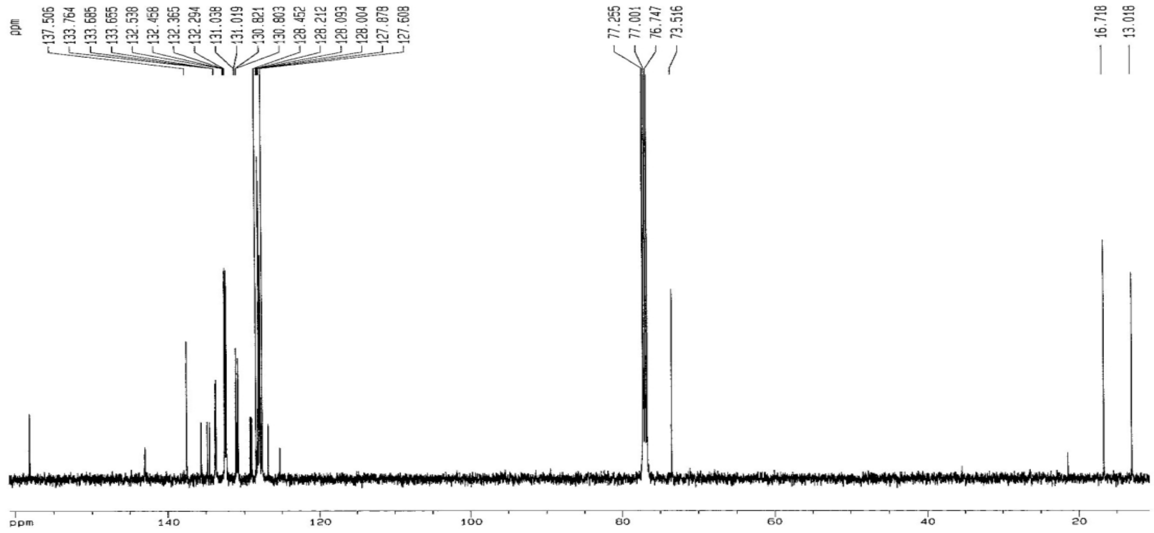
ppm
137.506
133.764
133.685
133.655
132.538
132.458
132.365
132.294
131.038
131.019
130.821
130.803
128.452
128.212
128.093
128.004
127.878
127.608
77.255
77.001
76.747
73.516
16.718
13.018
ppm
140
120
100
80
60
40
20

molecules

MDPI

(5,5'-dichloro-4,4',6,6'-tetramethylbiphenyl-2,2'-diyl)bis(diphenylphosphane) dioxide (11c)

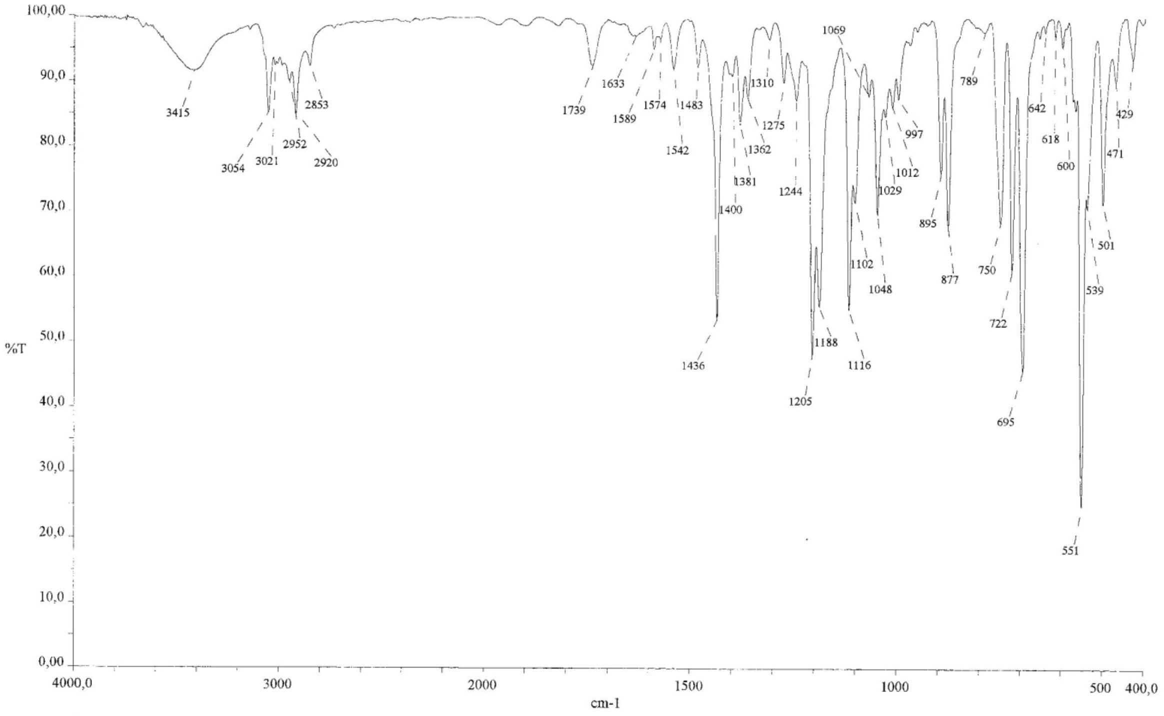

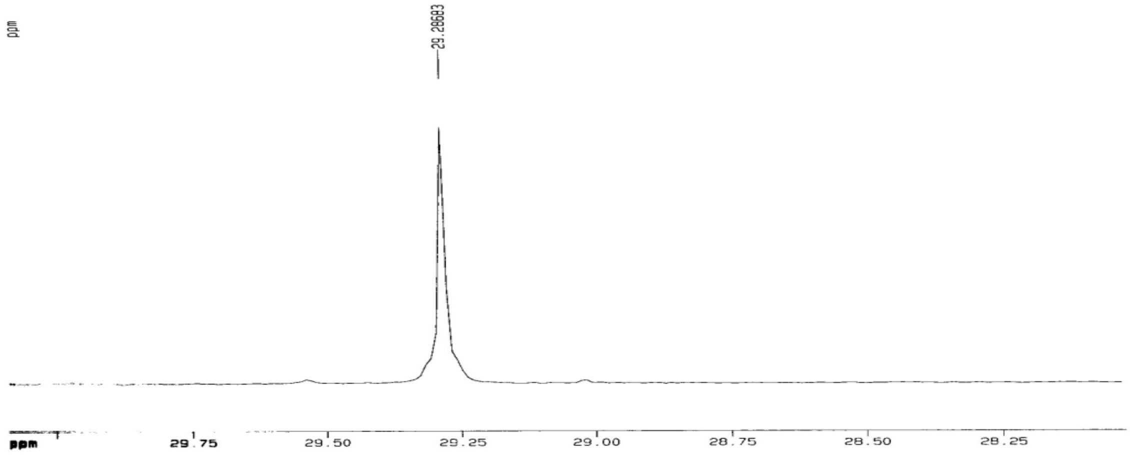

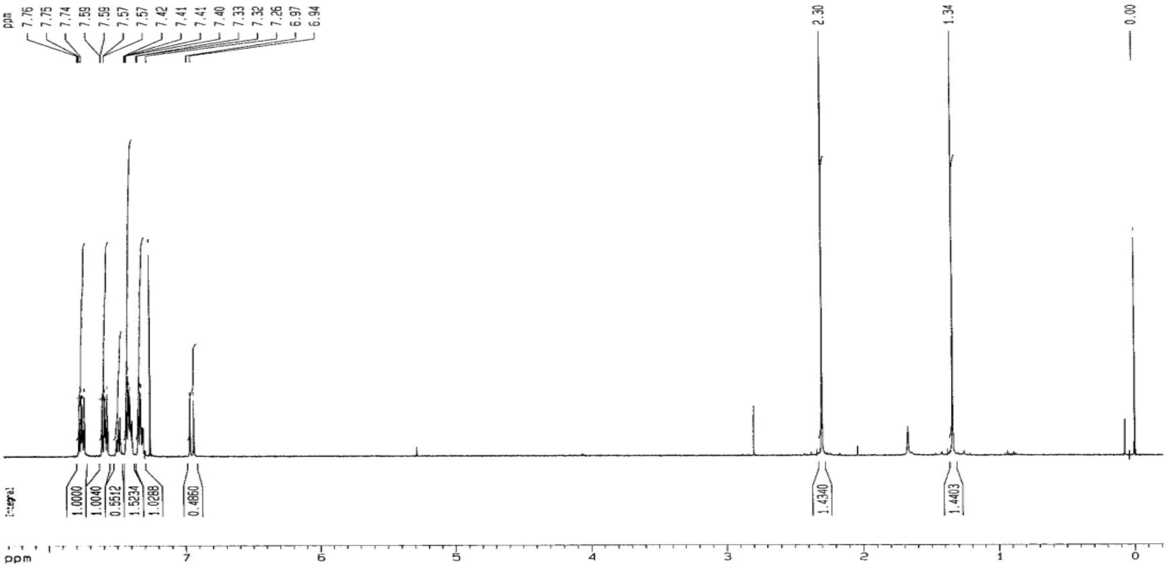
ppm
7.76 7.75 7.74 7.59 7.59 7.57 7.57 7.42 7.41 7.41 7.40 7.33 7.32 7.26 6.97 6.94
2.30
1.34
0.00
Integral
1.0000 1.0040 0.5512 1.5234 1.0288 0.4860
1.4340
1.4403
ppm 7 6 5 4 3 2 1 0

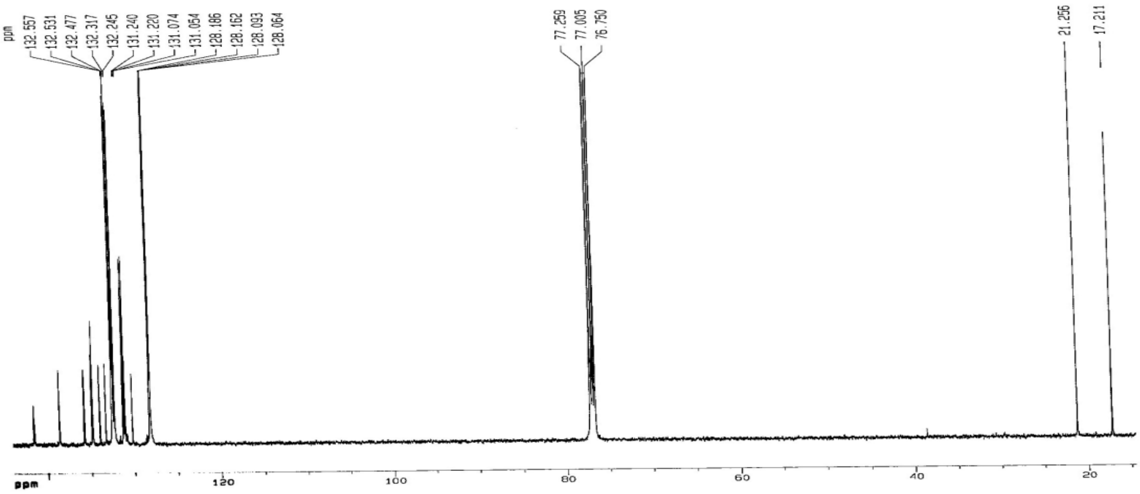
ppm
132.557 132.531 132.477 132.317 132.245 131.240 131.220 131.074 131.054 128.186 128.162 128.093 128.064
77.259 77.005 76.750
21.256
17.211
ppm 120 100 80 60 40 20

(3',5-dichloro-6'-iodo-2',4,4',6-tetramethylbiphenyl-2-yl)(diphenyl)phosphane oxide (13c)

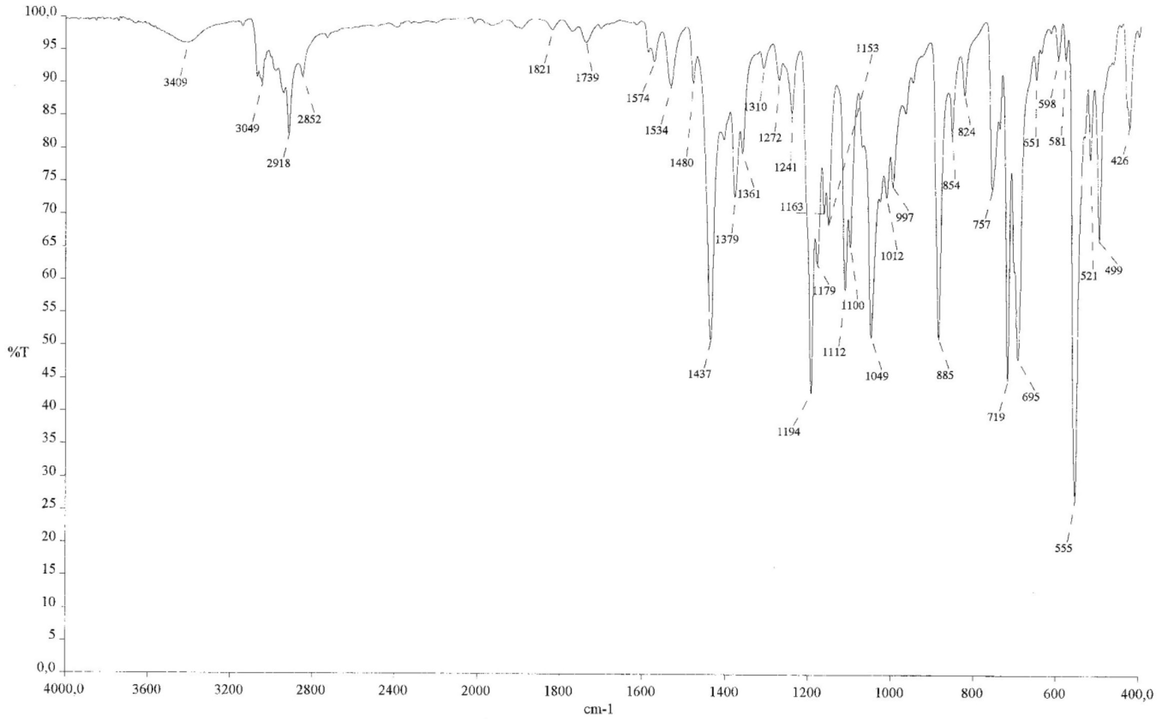

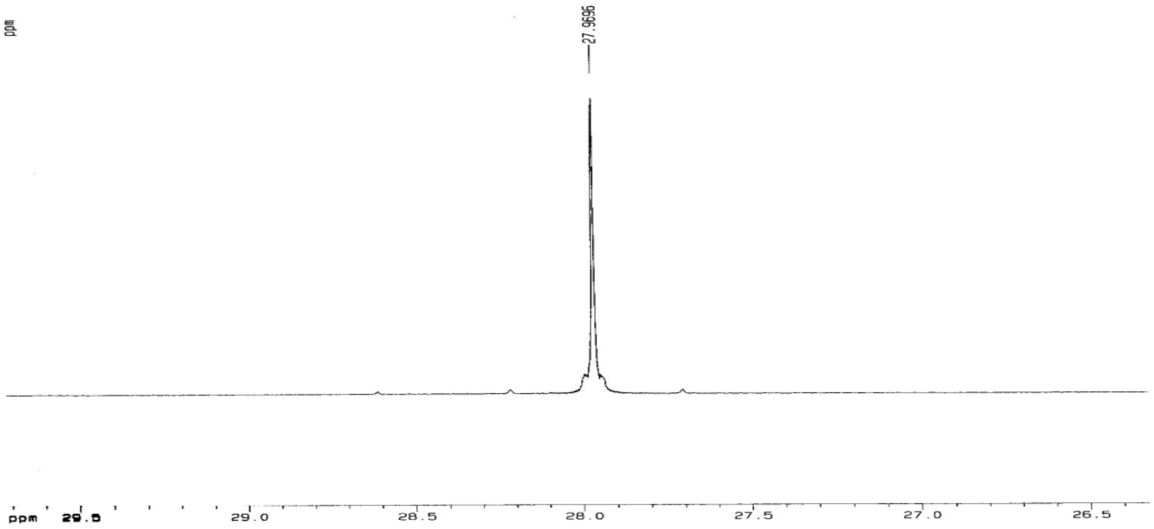

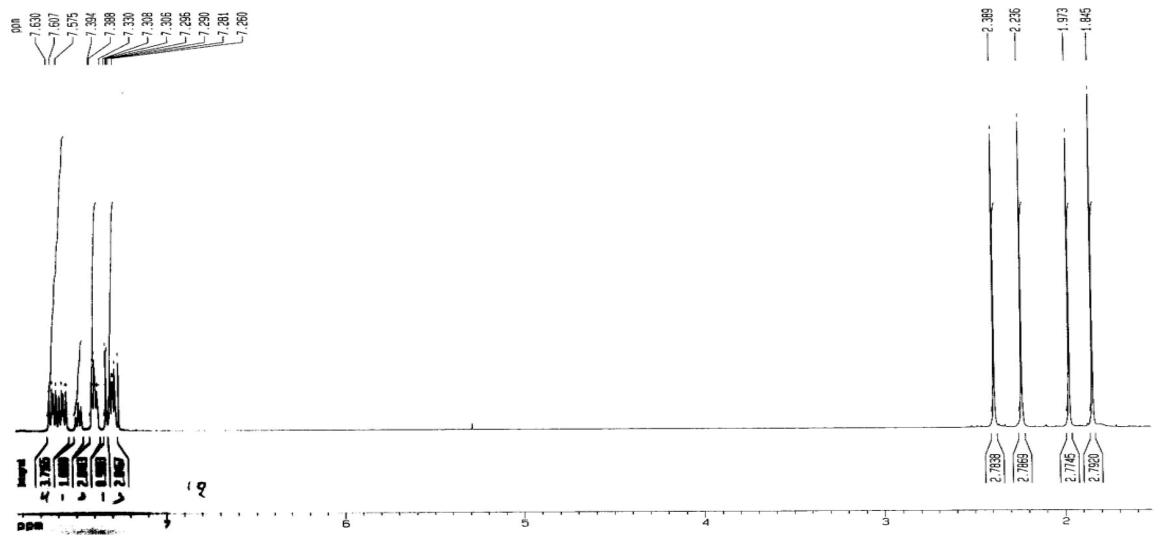
ppm
7.630
7.607
7.575
7.394
7.388
7.330
7.308
7.306
7.296
7.290
7.281
7.260
2.389
2.236
1.973
1.845
2.7838
2.7869
2.7745
2.7920
6
5
4
3
2

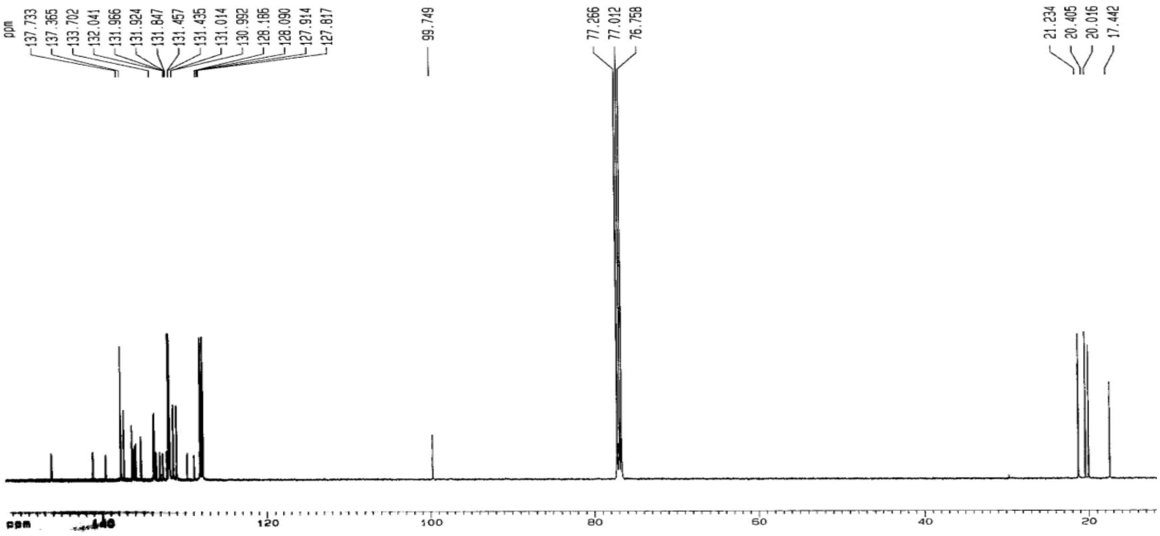
ppm
137.733
137.365
133.702
132.041
131.966
131.924
131.847
131.457
131.435
131.014
130.992
128.186
128.090
127.914
127.817
99.749
77.266
77.012
76.758
21.234
20.405
20.016
17.442
120
100
80
60
40
20

molecules

MDPI

(3',5-dichloro-2',4,4',6-tetramethylbiphenyl-2-yl)(diphenyl)phosphane oxide (12c)

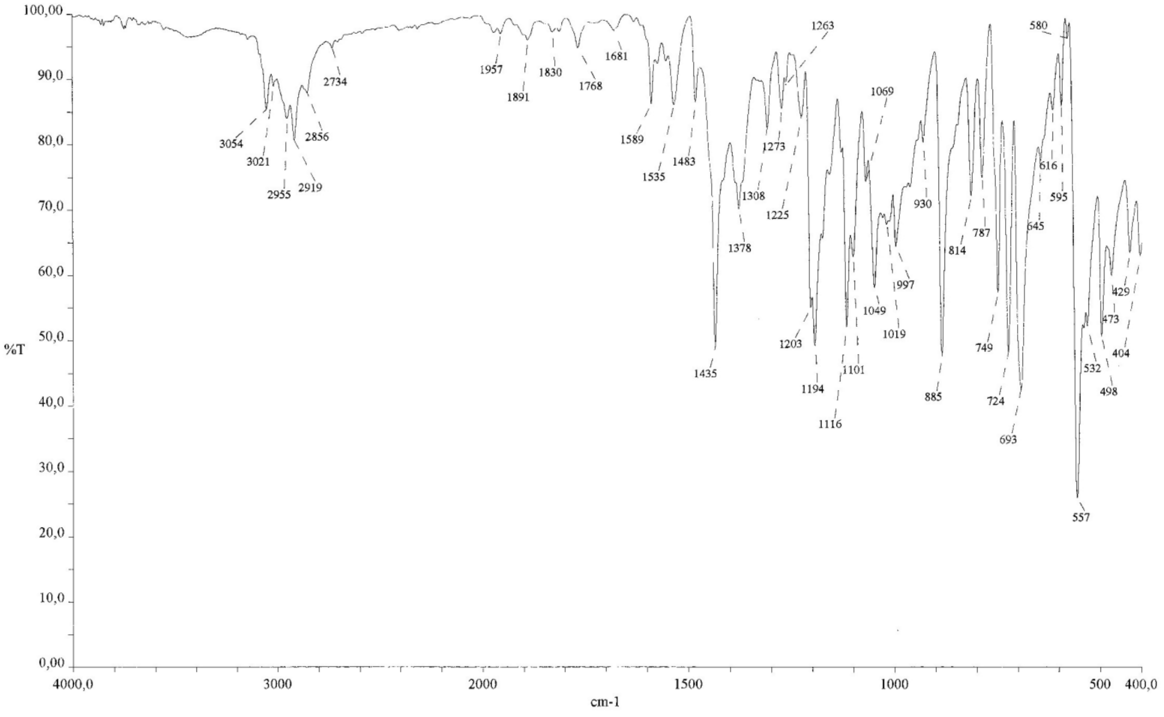

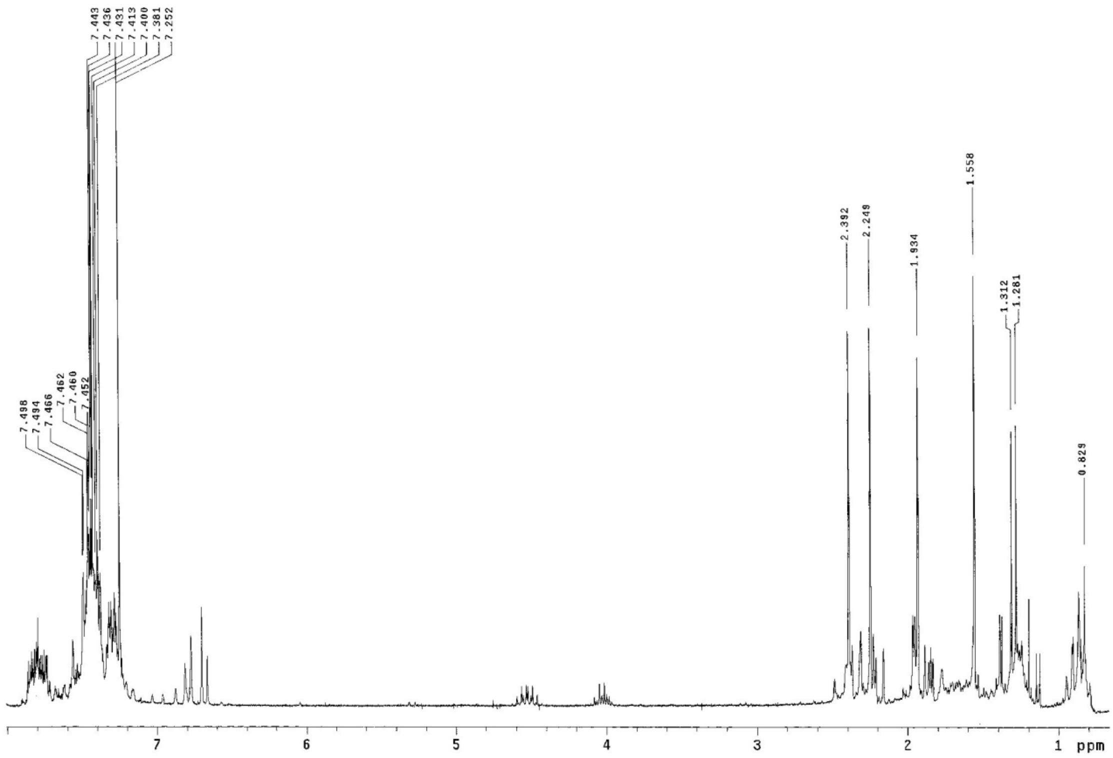

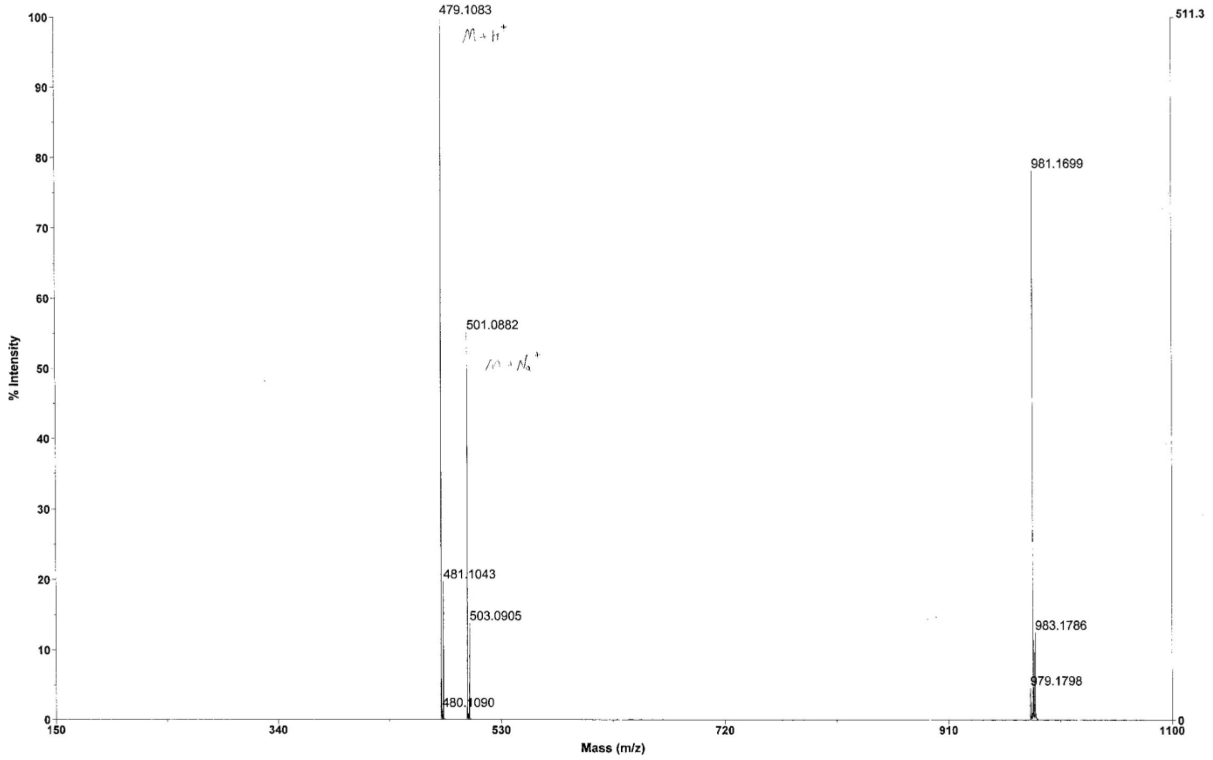

479.1083
M+H+
501.0882
M+Na+
481.1043
503.0905
480.1090
981.1699
983.1786
979.1798
511.3
% Intensity
Mass (m/z)
150
340
530
720
910
1100
100
90
80
70
60
50
40
30
20
10
0

(5,5'-dichloro-4,4',6,6'-tetramethylbiphenyl-2,2'-diyl)bis(diphenylphosphane) (4c)

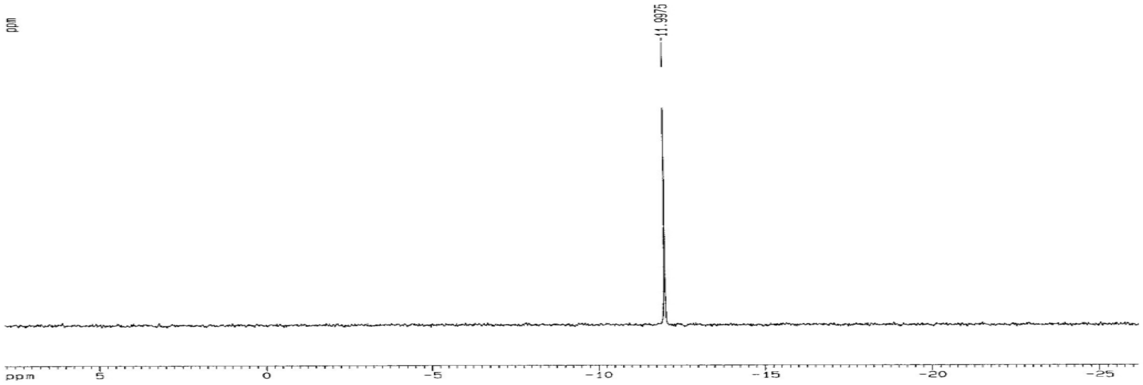

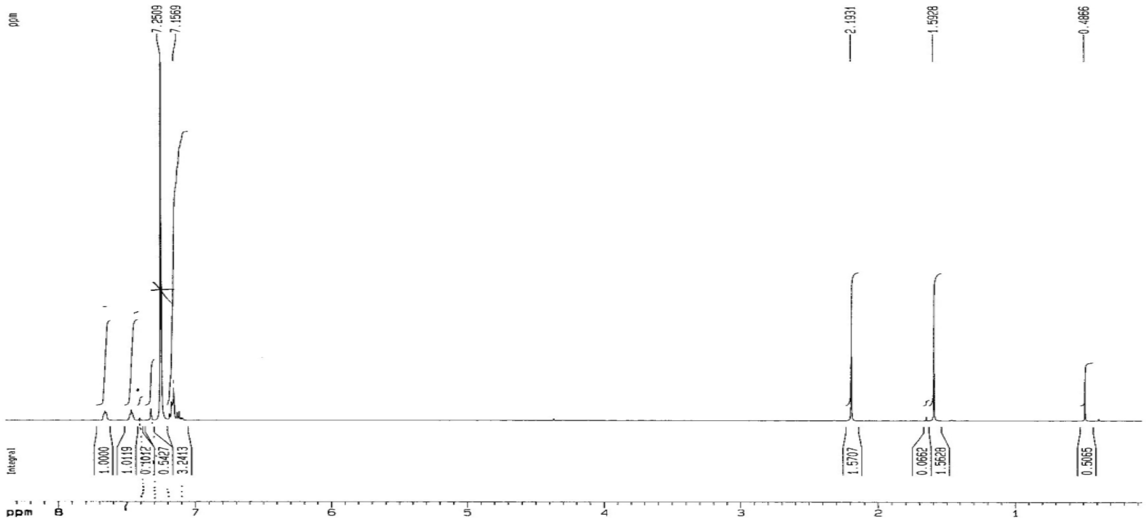

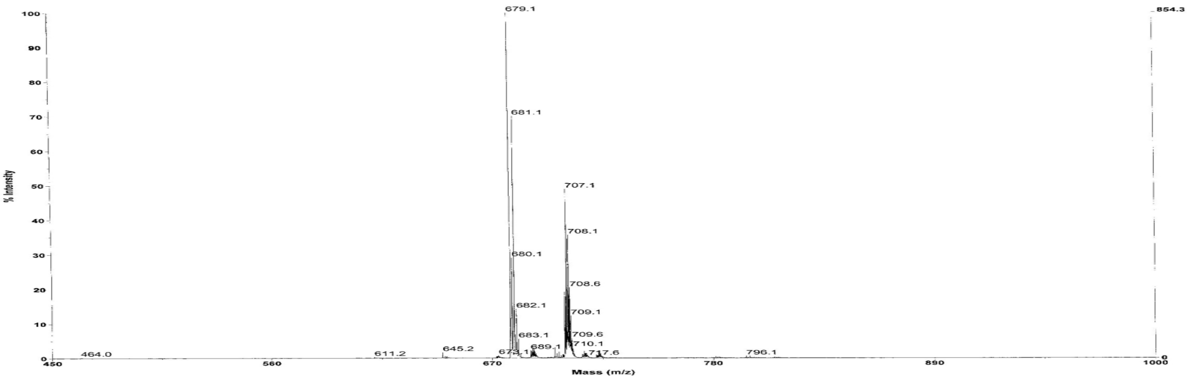

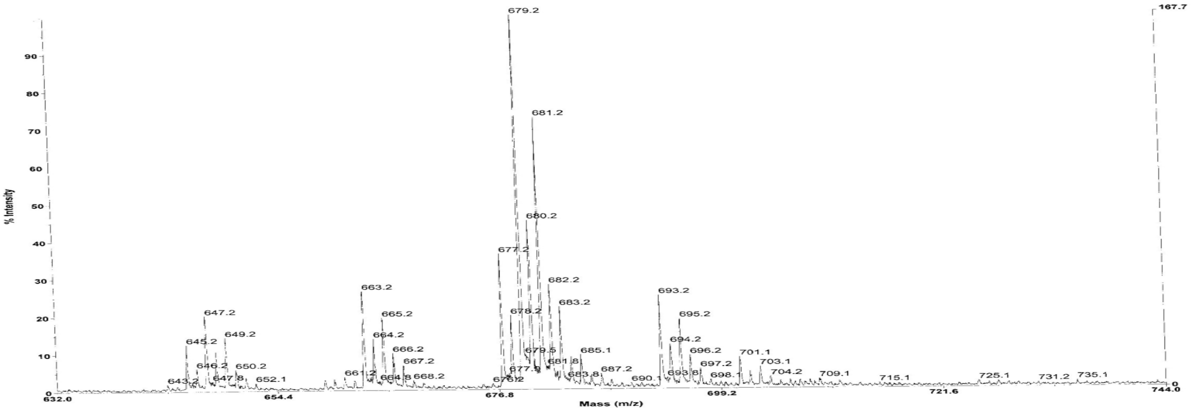

% Intensity
Mass (m/z)
0
10
20
30
40
50
60
70
80
90
632.0
654.4
676.8
699.2
721.6
744.0
167.7
679.2
681.2
680.2
677.2
682.2
683.2
678.2
679.5
685.1
663.2
665.2
664.2
666.2
667.2
668.2
661.2
647.2
649.2
645.2
646.2
650.2
652.1
643.2
693.2
695.2
694.2
696.2
697.2
698.1
701.1
703.1
704.2
709.1
715.1
725.1
731.2
735.1
687.2
690.1

molecules MDPI

6,6'-bis(diphenylphosphoryl)-N,N,N',N',2,2',4,4'-octamethylbiphenyl-3,3'-diamine (11b)

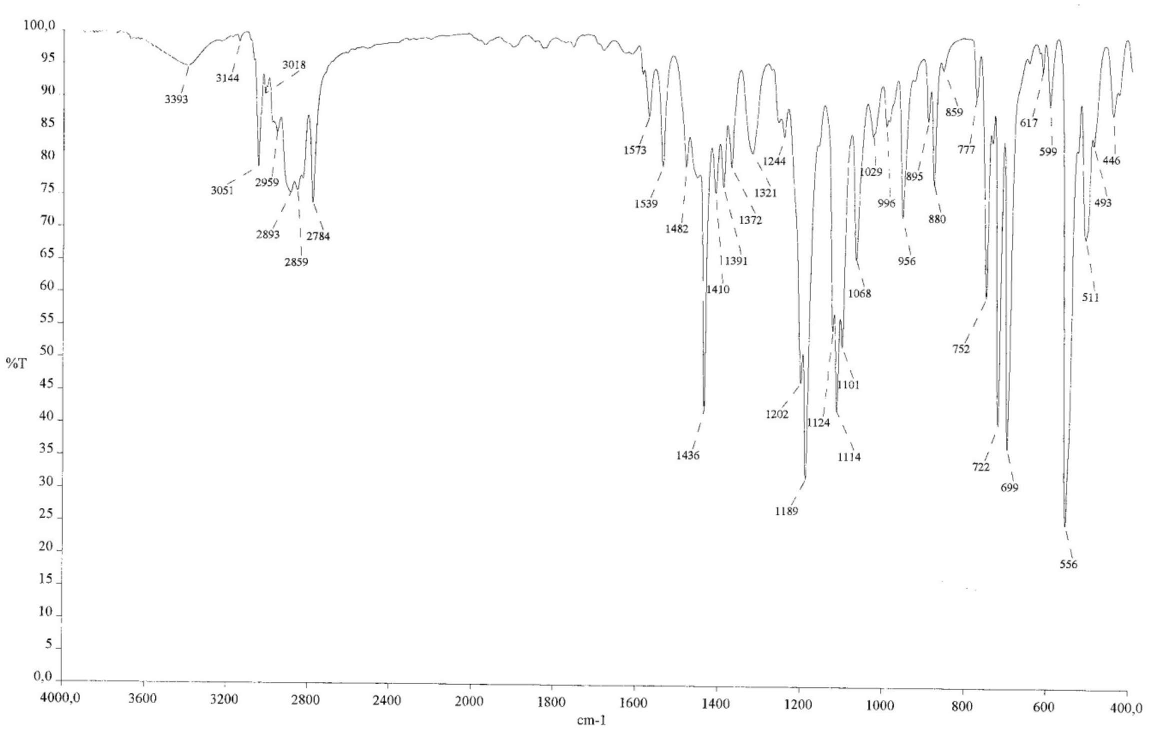

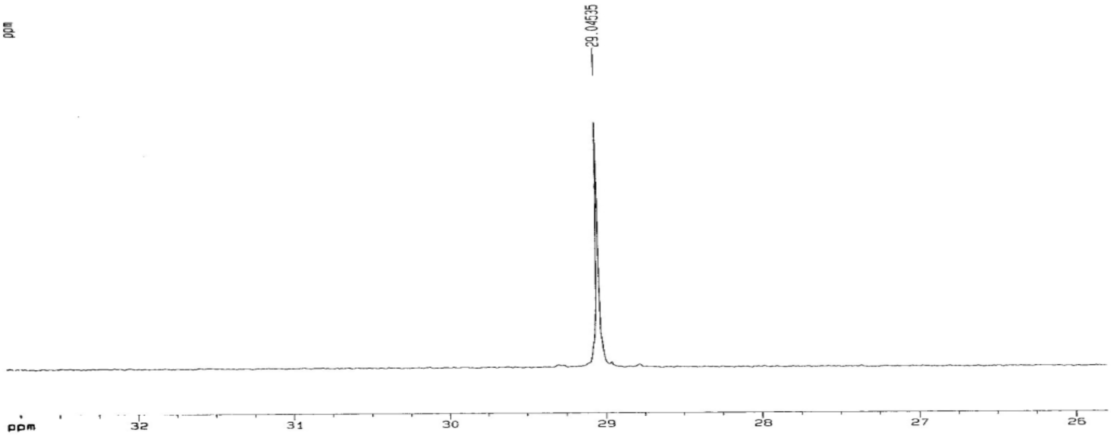

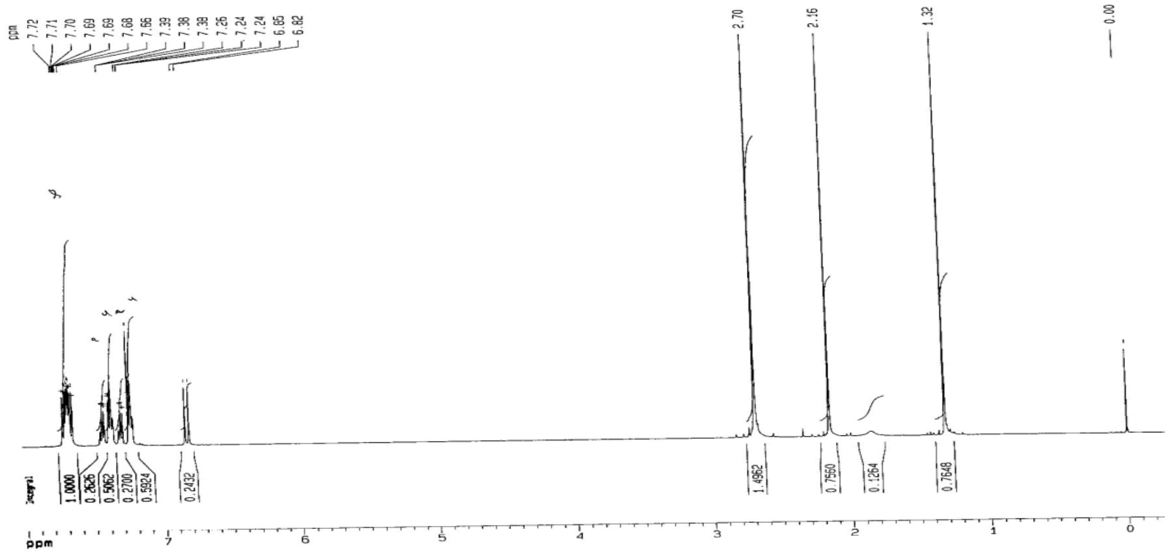
ppm
7.72
7.71
7.70
7.69
7.69
7.68
7.66
7.39
7.38
7.38
7.26
7.24
7.24
6.85
6.82
2.70
2.16
1.32
0.00
1.0000
0.2626
0.5062
0.2700
0.5924
0.2432
1.4962
0.7560
0.1264
0.7648
ppm
7
6
5
4
3
2
1
0

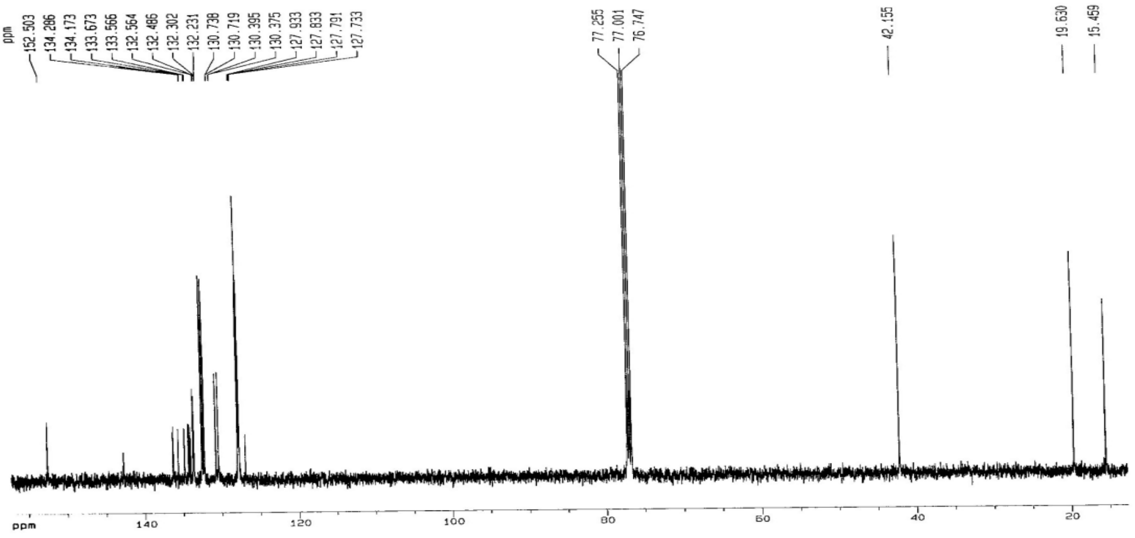
ppm
152.503
134.286
134.173
133.673
133.566
132.564
132.486
132.302
132.231
130.738
130.719
130.395
130.375
127.933
127.833
127.791
127.733
77.255
77.001
76.747
42.155
19.630
15.459
ppm
140
120
100
80
60
40
20

6-(diphenylphosphoryl)-N,N,N',N',2,2',4,4'-octamethylbiphenyl-3,3'-diamine (12b)

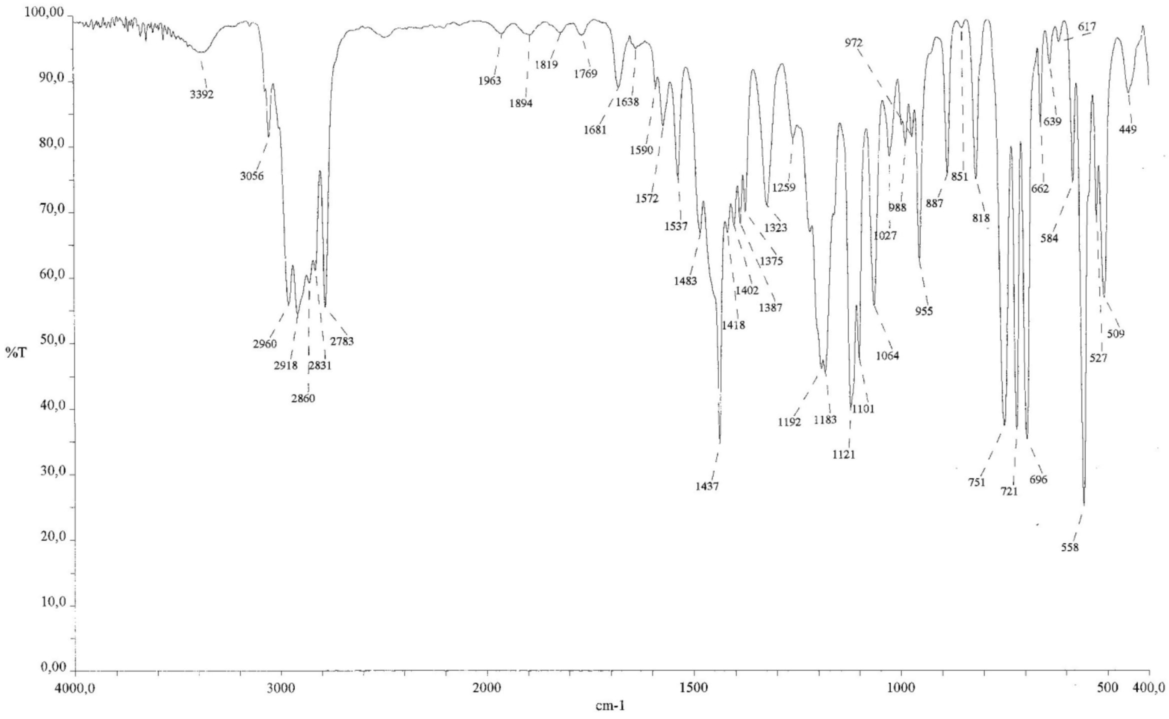

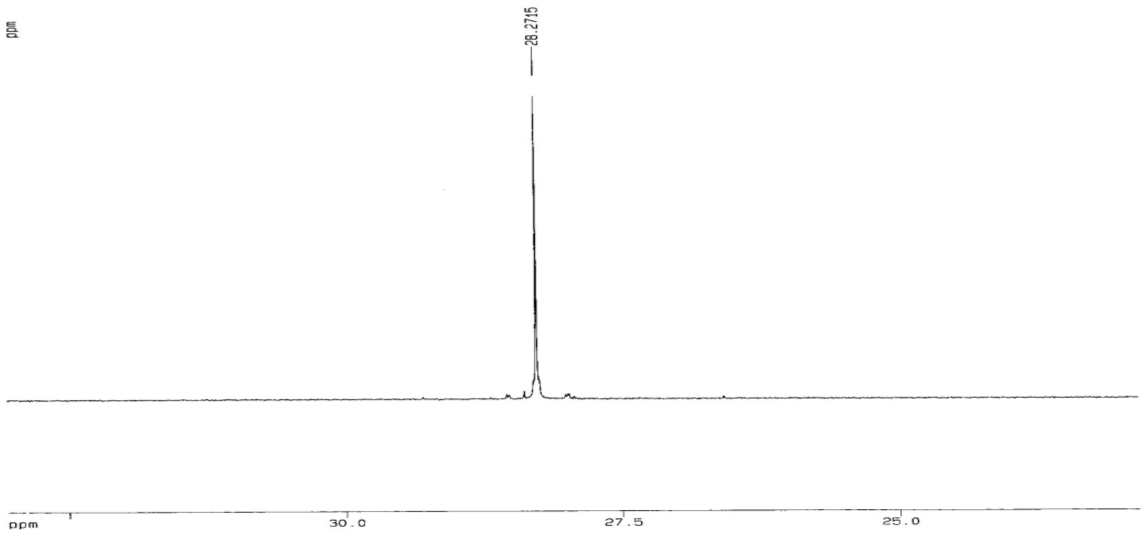

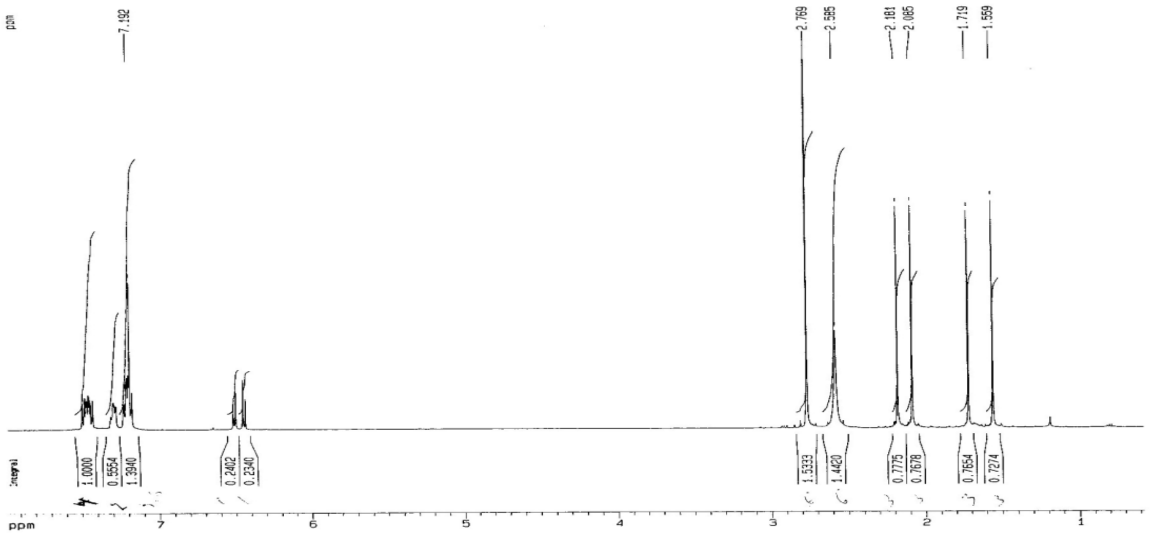
ppm
7.192
2.769
2.595
2.181
2.085
1.719
1.559
Integral
1.0000
0.5554
1.3940
0.2402
0.2340
1.5333
1.4420
0.7775
0.7678
0.7654
0.7274
ppm
7
6
5
4
3
2
1

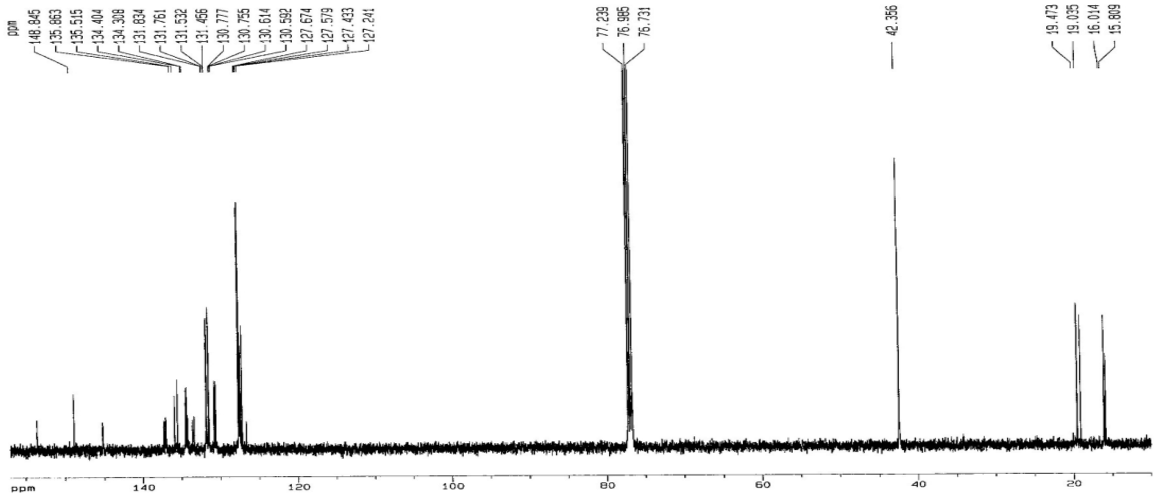
ppm
148.845
135.863
135.515
134.404
134.308
131.834
131.761
131.532
131.456
130.777
130.755
130.614
130.592
127.674
127.579
127.433
127.241
77.239
76.985
76.731
42.356
19.473
19.035
16.014
15.809
ppm
140
120
100
80
60
40
20

molecules

MDPI

6,6'-bis(diphenylphosphanyl)-N,N,N',N',2,2',4,4'-octamethylbiphenyl-3,3'-diamine (4b)

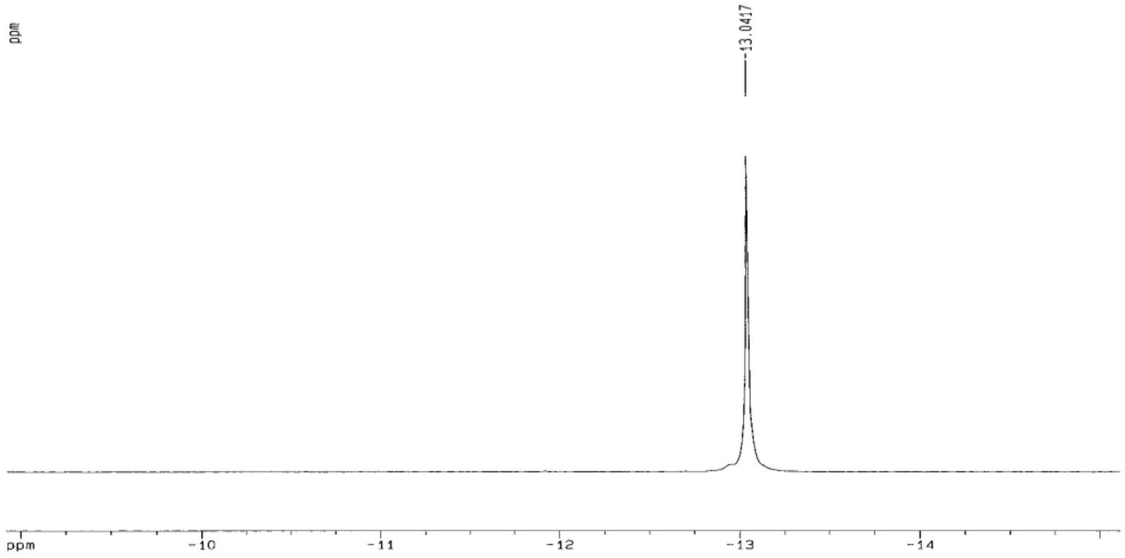

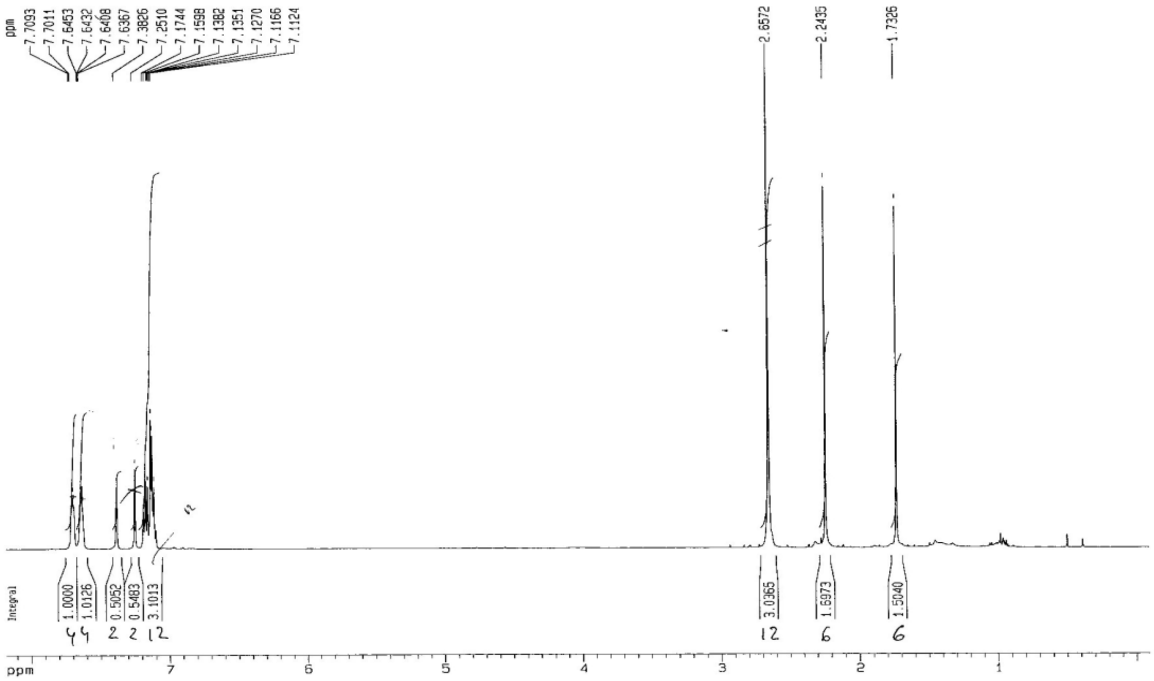

N,N,N',N'-tetrabutyl-6,6'-bis(diphenylphosphoryl)-2,2',4,4'-tetramethylbiphenyl-3,3'-diamine (11e)

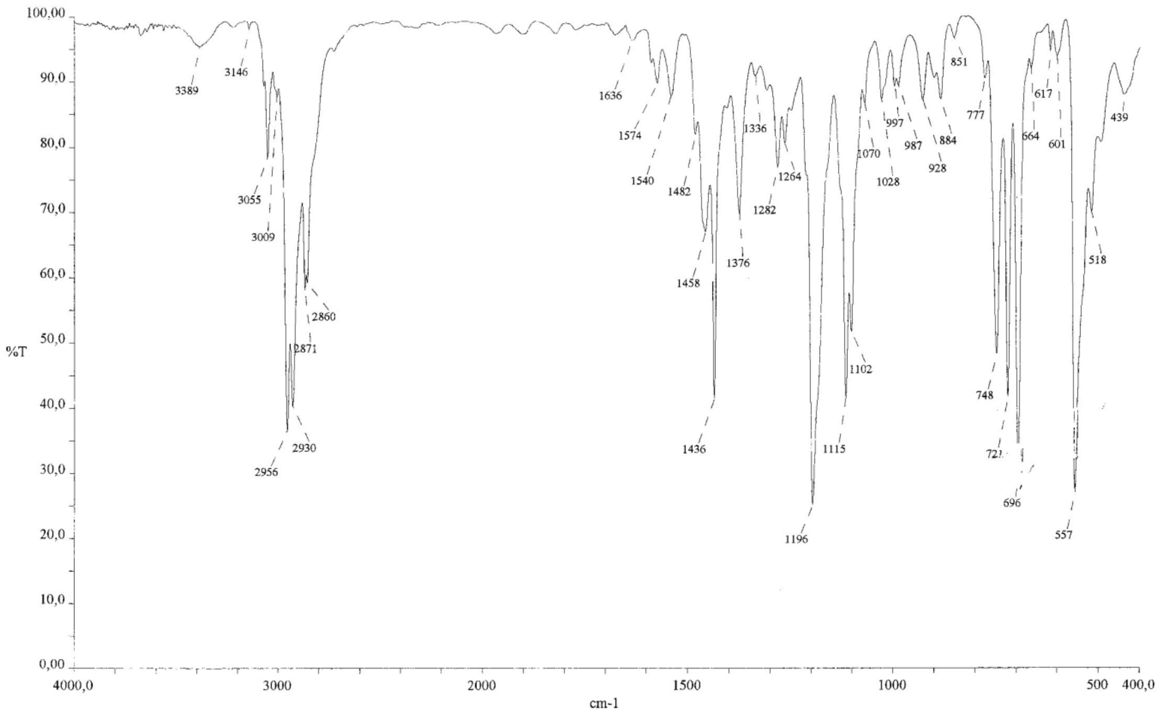

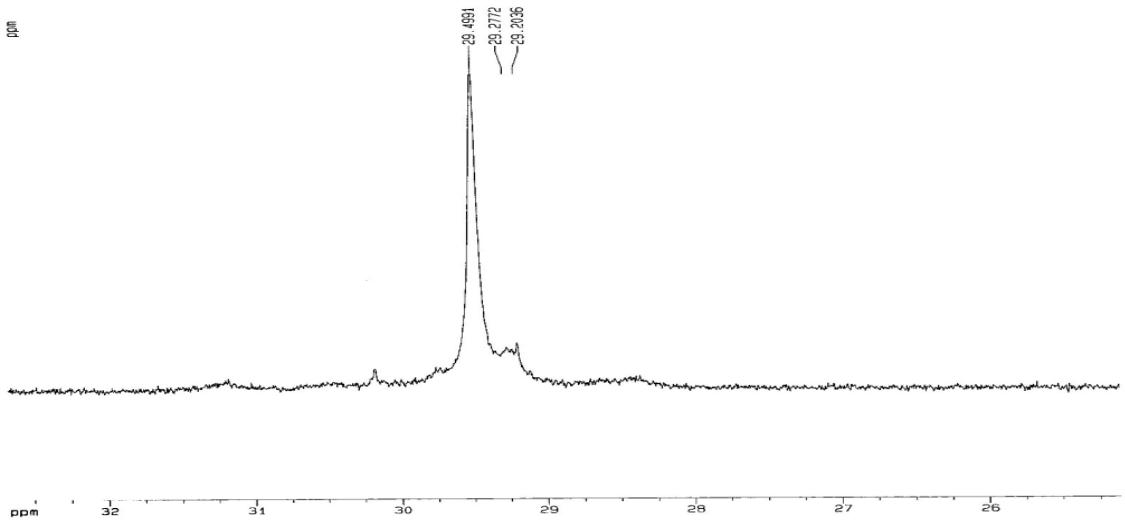

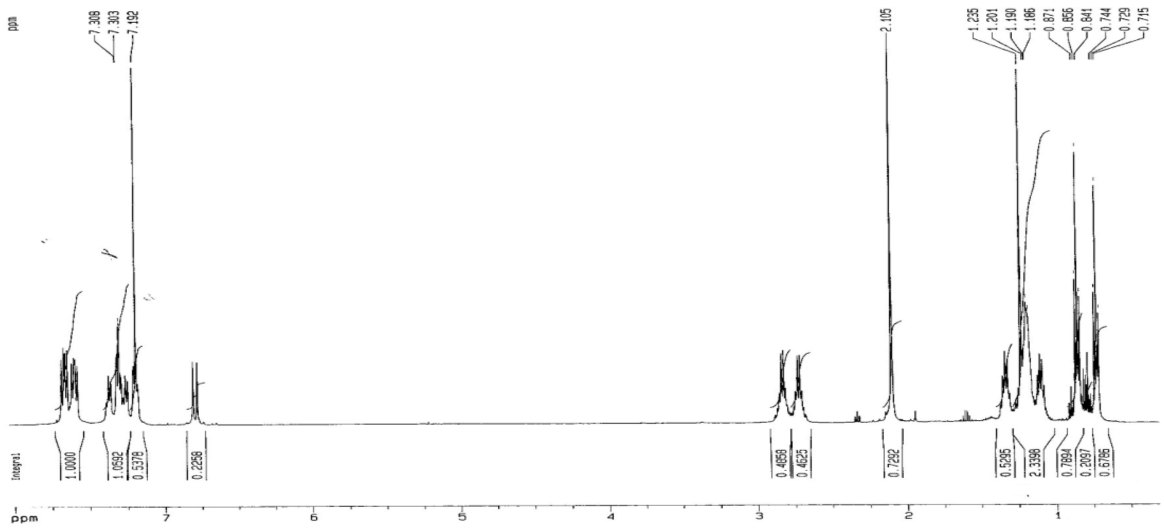
ppm
7.308
7.303
7.192
2.105
1.235
1.201
1.190
1.186
0.871
0.856
0.841
0.744
0.729
0.715
Integral
1.0000
1.0592
0.5378
0.2258
0.4656
0.4625
0.7292
0.5295
2.3398
0.7894
0.2097
0.6786
ppm
7
6
5
4
3
2
1

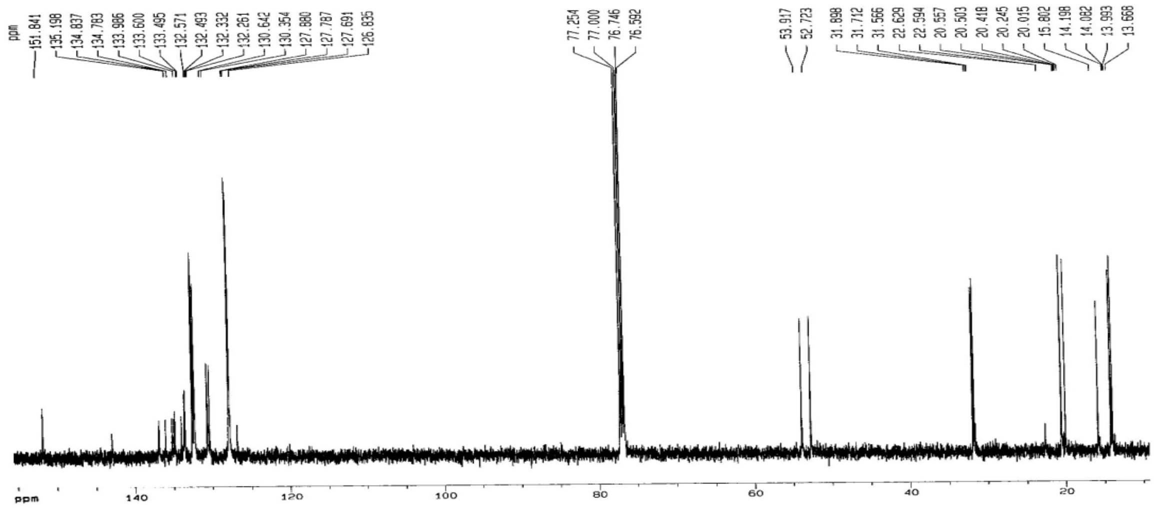
ppm
151.841
135.198
134.837
134.783
133.986
133.600
133.495
132.571
132.493
132.332
132.261
130.642
130.354
127.880
127.787
127.691
126.835
77.254
77.000
76.746
76.592
53.917
52.723
31.898
31.712
31.566
22.629
22.594
20.557
20.503
20.418
20.245
20.015
15.802
14.198
14.082
13.993
13.668
ppm
140
120
100
80
60
40
20

molecules

MDPI

(4,4',6,6'-tetramethylbiphenyl-2,2'-diyl)bis(diphenylphosphane) dioxide (11d)

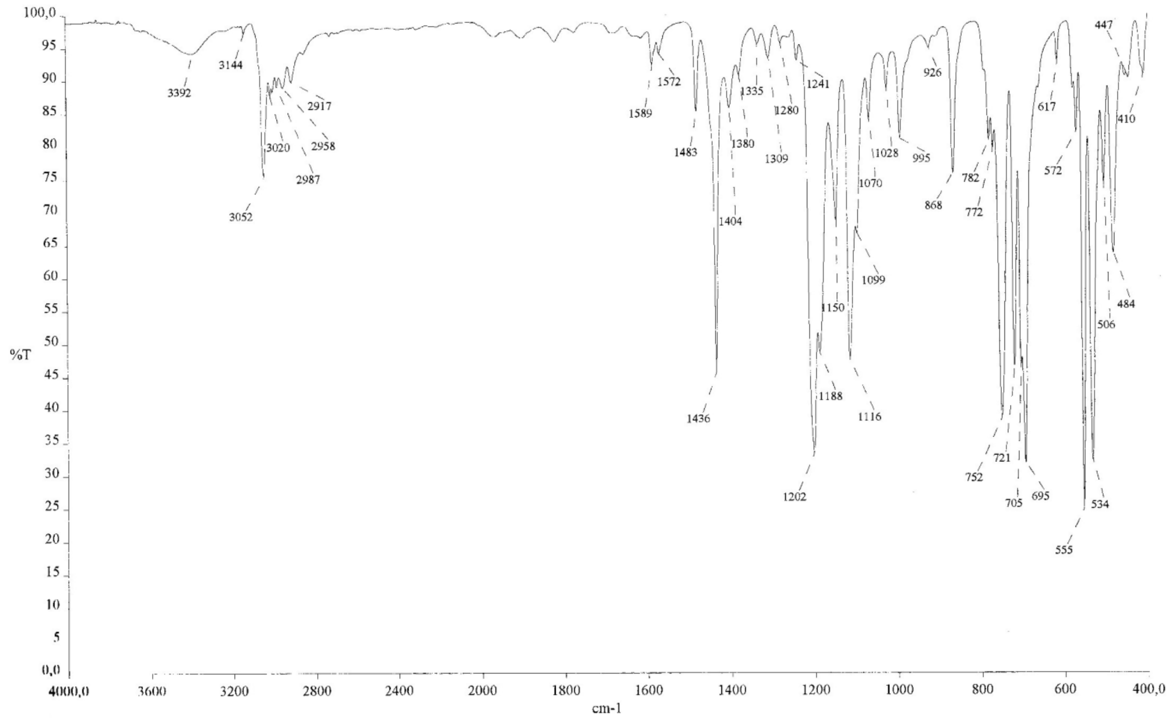

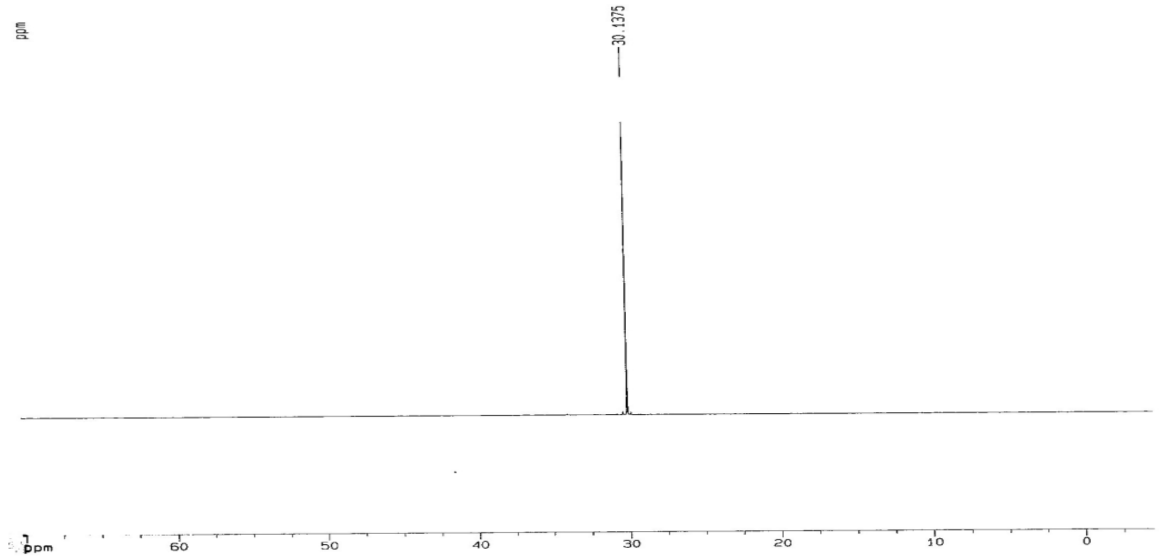

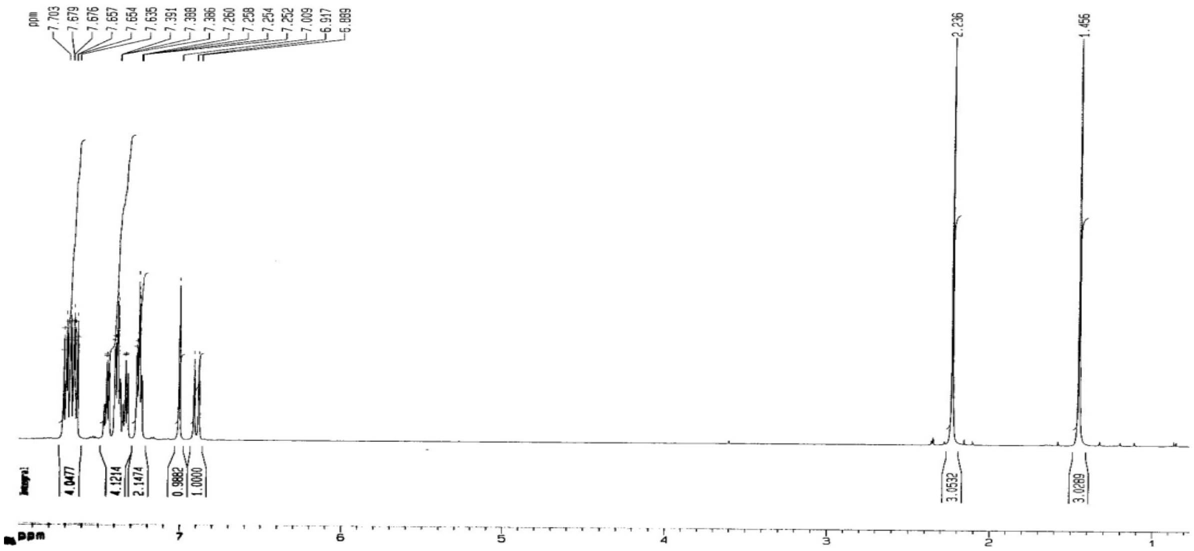

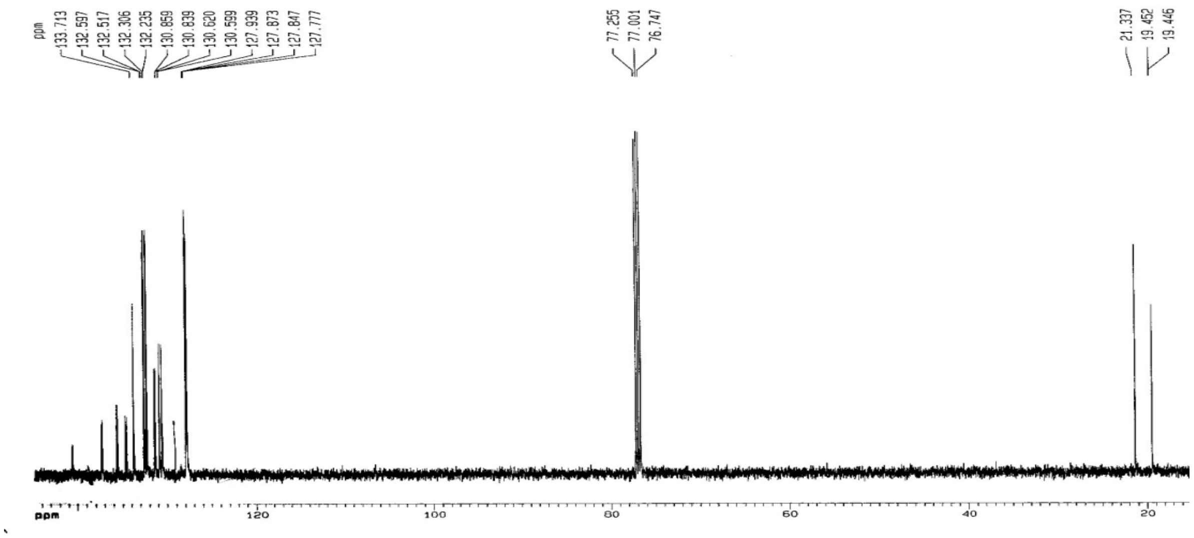

molecules

diphenyl(2',4,4',6-tetramethylbiphenyl-2-yl)phosphane oxide (12d)

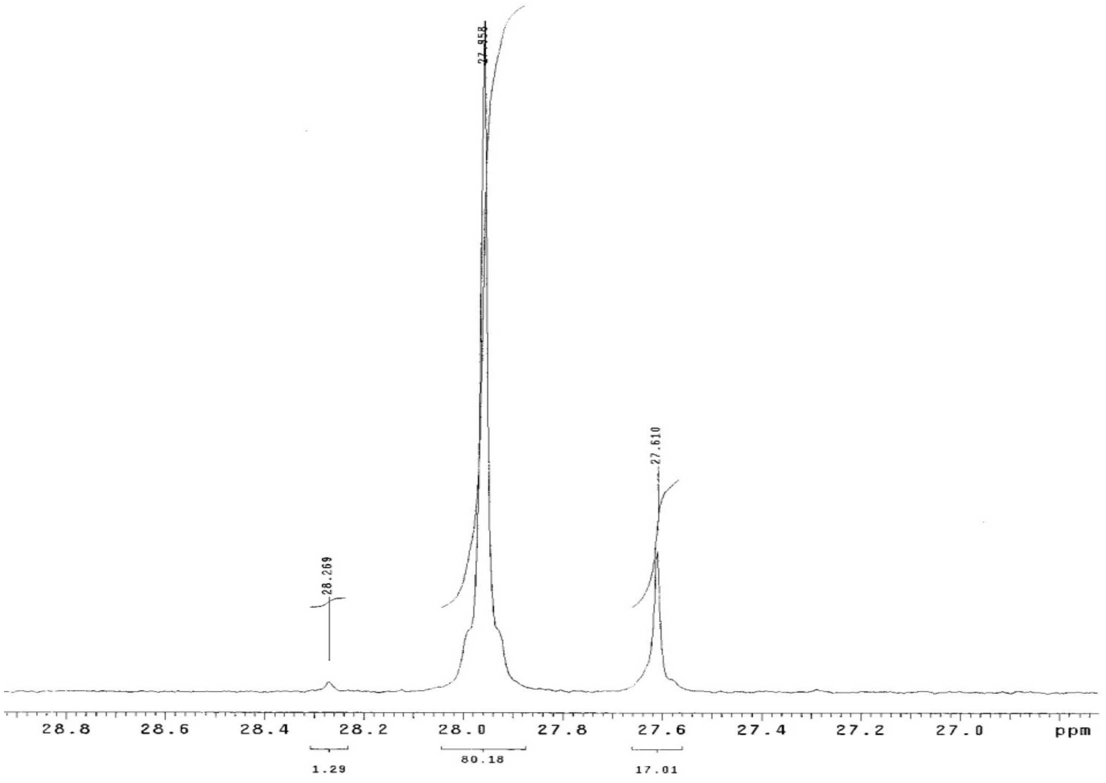

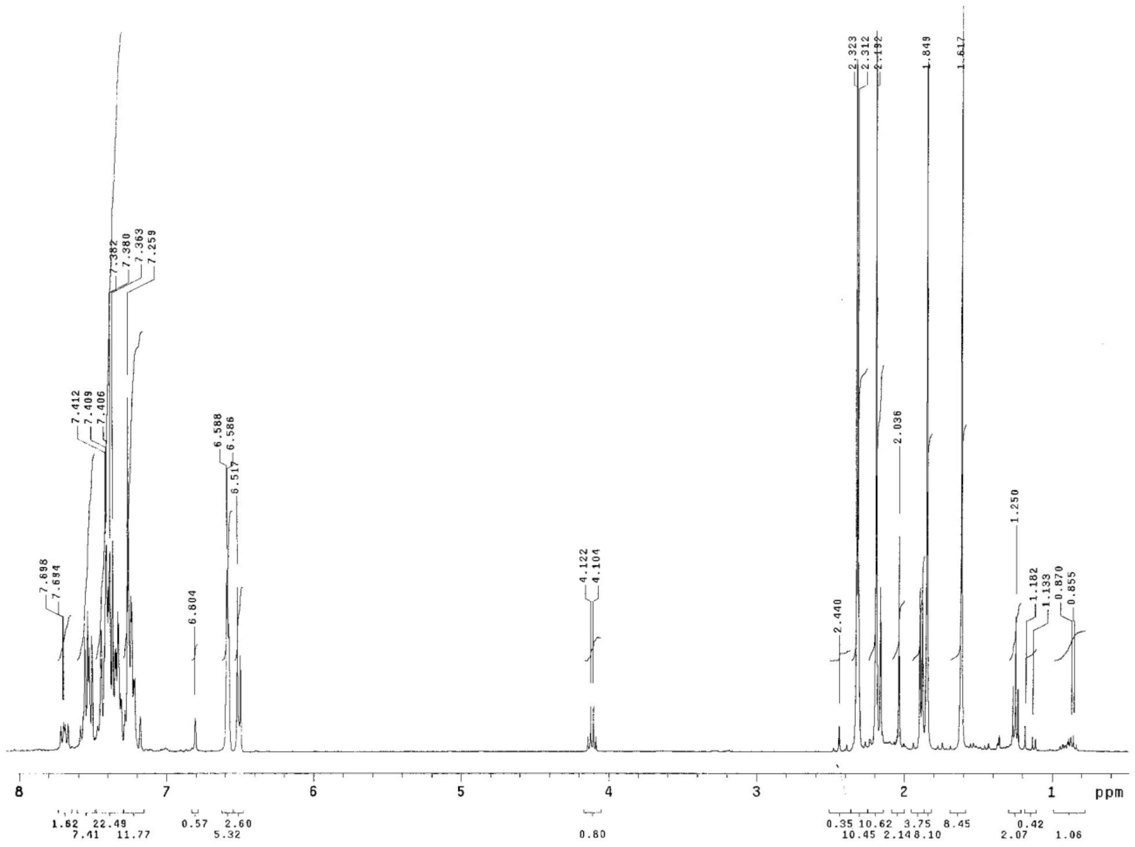

Molecules **2022**, *27*, 5504. https://doi.org/10.3390/molecules27175504
